# Functional and transcriptional profiling of microglial activation during the chronic phase of TBI identifies an age-related driver of poor outcome in old mice

**DOI:** 10.1007/s11357-022-00562-y

**Published:** 2022-04-22

**Authors:** Rodney M. Ritzel, Yun Li, Zhuofan Lei, Jordan Carter, Junyun He, Harry M. C. Choi, Niaz Khan, Hui Li, Samantha Allen, Marta M. Lipinski, Alan I. Faden, Junfang Wu

**Affiliations:** Department of Anesthesiology and Shock, Trauma and Anesthesiology Research Center, University of Maryland School of Medicine, Baltimore, MD 21201 USA

**Keywords:** Aging, Autophagy, Microglia, Neurodegeneration, Neuroinflammation, Traumatic brain injury

## Abstract

Elderly patients with traumatic brain injury (TBI) have greater mortality and poorer outcomes than younger individuals. The extent to which old age alters long-term recovery and chronic microglial activation after TBI is unknown, and evidence for therapeutic efficacy in aged mice is sorely lacking. The present study sought to identify potential inflammatory mechanisms underlying age-related outcomes late after TBI. Controlled cortical impact was used to induce moderate TBI in young and old male C57BL/6 mice. At 12 weeks post-injury, aged mice exhibited higher mortality, poorer functional outcomes, larger lesion volumes, and increased microglial activation. Transcriptomic analysis identified age- and TBI-specific gene changes consistent with a disease-associated microglial signature in the chronically injured brain, including those involved with complement, phagocytosis, and autophagy pathways. Dysregulation of phagocytic and autophagic function in microglia was accompanied by increased neuroinflammation in old mice. As proof-of-principle that these pathways have functional importance, we administered an autophagic enhancer, trehalose, in drinking water continuously for 8 weeks after TBI. Old mice treated with trehalose showed enhanced functional recovery and reduced microglial activation late after TBI compared to the sucrose control group. Our data indicate that microglia undergo chronic changes in autophagic regulation with both normal aging and TBI that are associated with poorer functional outcome. Enhancing autophagy may therefore be a promising clinical therapeutic strategy for TBI, especially in older patients.

**Supplementary Information:**

The online version contains supplementary material available at 10.1007/s11357-022-00562-y.

## Introduction

Neurodegenerative disease in the elderly is a major healthcare challenge and growing economic burden. Traumatic brain injury (TBI), a leading cause of death among young people, is increasingly prevalent in the aging population. Millions of TBI survivors live with varying degrees of disability and have an increased risk for developing neurodegenerative disease such as Alzheimer’s disease (AD), Parkinson’s disease, and chronic traumatic encephalopathy [3, 23]. Elderly TBI patients have higher mortality and greater disability than younger individuals and are more likely to develop a neurodegenerative disorder [15, 17, 18]. For these reasons, older subjects are often excluded from clinical trials and treated less aggressively [19].

Our knowledge of TBI pathophysiology in aged animals is limited, and our understanding of the secondary injury mechanisms that affect long-term outcome remains poor. Whereas direct targeting of inflammatory pathways in old mice appears beneficial in experimental settings [31, 45], therapeutic intervention of other proposed injury mechanisms, such as autophagy [54], has not been addressed in older animals. Both normal aging and the chronic phase of TBI are associated with a basal elevation in microglial activation and neuroinflammation. Chronic activation of microglia has been linked to alterations in cellular functions essential for survival [33]. The emergence of a disease-associated microglial (DAM) gene signature is viewed as a universal sensor of neurodegeneration [10]. This signature has recently been identified in experimental TBI [70], however has yet to be confirmed in old mice and at chronic timepoints (> 30 days) after injury. Moreover, validation at the level of cellular function is currently lacking. Among the DAM-related gene pathways, autophagy is a process that degrades and recycles cytoplasmic proteins and organelles; and it plays a critical role in inflammation, the homeostasis of microglia, and plaque development in AD [5, 13]. Autophagic mechanisms have been found to be impaired with age and during acute brain injury [40, 71]. However, whether aging and TBI synergistically interact to disrupt autophagic function in microglia, aggravate neuroinflammation, and hamper long-term neurological recovery is still unclear.

Despite recent funding and research efforts towards understanding age-related neurodegenerative disease, the secondary injury mechanisms that account for the age-related disparity in TBI outcomes remain understudied. Given the limited number of pre-clinical studies examining injury mechanisms and related outcomes in aged animal models of experimental TBI [26, 67], the endogenous recovery mechanisms impaired in older age have yet to be identified, including whether these pathways may remain amenable to therapeutic modulation. Here, we examine the hypothesis that age-related defects in autophagic function can exacerbate chronic microglial activation, neurodegeneration, and long-term functional recovery in old mice which can be partially rescued by enhancing autophagic function.

## Methods

### Animals and TBI model

All surgical procedures and animal experiments were performed according to the protocols approved by the University of Maryland School of Medicine Institutional Animal Care and Use Committee (IACUC). Experiments were conducted using young adult male C57BL/6 mice (10–12 weeks old) or aged male mice (18 months old) from Charles River. After being fully anesthetized with isoflurane, mice were subjected to either controlled cortical injury (CCI) or sham surgery [57, 58]. Briefly, a midline incision of approximately 10 mm in length was made over the skull, with the skin and fascia retracted, and a 4-mm craniotomy was made on the central aspect of the left parietal bone. An injury of moderate severity was induced by a TBI-0310 Head Impactor (Precision Systems and Instrumentation) with a 3.5-mm diameter tip followed by impact velocity of 3.9 m/s and a displacement depth of 1.2 mm. After surgery, all mice were assigned to one of four groups based on surgery (sham or TBI) and treatment (trehalose or sucrose control) according to a randomized block experimental design. The surgical procedures were performed by the same investigator and all behavioral tests were carried out with the same equipment by blinded experimenters.

### Experimental design

#### Study 1

**Fig. 1:**
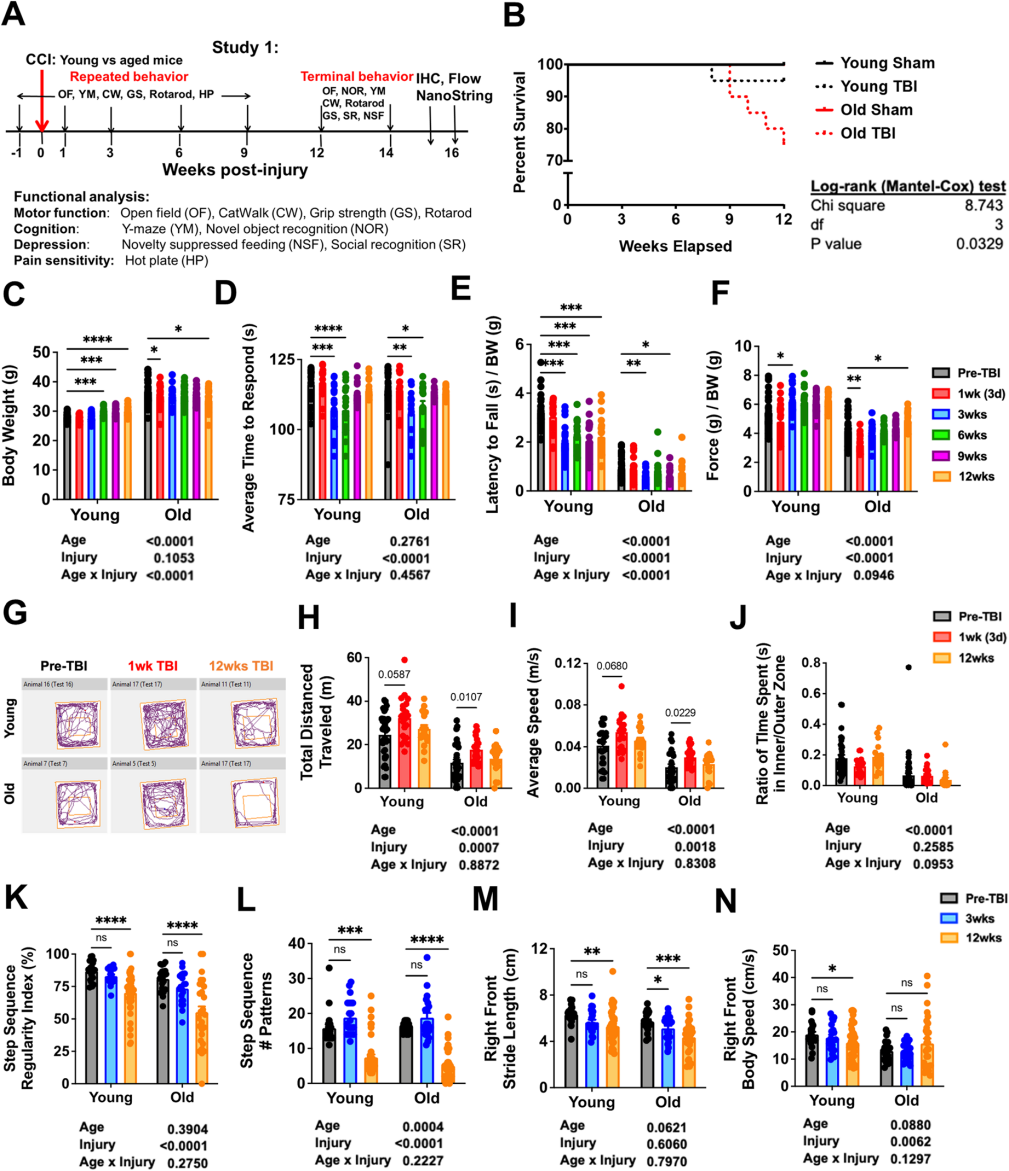
Effects of age on long-term survival and motor recovery after TBI. **A** Schematic diagram illustrating timepoints by weeks pre- or post-injury and of repeated or terminal behavior experiments conducted throughout study 1. Baseline behavior testing aimed at assessment of motor, cognition, and pain sensitivity was performed at 1 week before injury, followed by a moderate controlled cortical impact (CCI) injury on the left cerebral cortex of mice randomly designated to the injury groups. Repeated behavior tests were performed on 1, 3, 6, and 9 weeks post-injury. Terminal behavior for motor, cognitive, and depression-like behavior were carried out between 12 and 14 weeks post-injury before euthanizing the mice for immunohistochemistry, flow cytometry, and NanoString analysis. **B** Survival plot of young and old mice in weeks elapsed for study 1. Compared to old sham (*N* = 12) and young sham (*N* = 13), both TBI injury groups (*N* = 20/group) show decreased survival rates, with old TBI group having the lowest survival rate of all mice. Kaplan–Meier survival curves were analyzed using the log-rank Mantel-Cox test. **C** Graph depicting body weight data of young and old mice at baseline and follow-up measurements post-injury. **D** Graph depicting latency time to withdrawal in hot plate test for thermal sensitization. Both age groups displayed thermal hyperalgesia between 3 and 6 weeks post-injury, but no age effect was observed. **E** Graph depicting rotarod data from baseline to 12 weeks post-injury. **F** Graph depicting grip strength measurements from baseline to 12 weeks post-injury. **G** Representative graph of spontaneous movement by young and old mice in the open field test at baseline, 3 weeks, and 12 weeks after TBI. **H**–**J** Spontaneous locomotor activity in an open field apparatus was recorded and analyzed with use of the AnyMaze animal behavior system. Injury effects could be observed in two parameters: total distance travelled **(H)** and average speed **(I)**. Age effects could be observed in all three parameters depicted. **K**–**N** Graphs depicting parameters of CatWalk gait analysis tested at baseline, 3 and 12 weeks post-injury. Two-way ANOVA followed by Tukey’s post hoc test. ****p < 0.0001, ***p < 0.001, ** *p* < 0.01, * *p* < 0.05

To investigate whether age affects neurological function and pathological changes after TBI, young or aged C57BL/6 male mice were subjected to either moderate CCI or sham surgery (Fig. 1A). All mice underwent a battery of neurobehavioral tasks which consisted of open field (OF), catwalk (CW), grip strength (GS), and rotarod for assessment of motor function; Y-maze (YM) and novel object recognition (NOR) for cognitive function; novelty suppressed feeding (NSF) and social recognition (SR) for depressive-like behaviors; and hot plate for pain sensitivity. The OF, YM, CW, GS, rotarod, and HP tests were performed starting at 4 days before CCI for establishment of functional performance baseline between young and old mice and repeatedly at 1 week, 3, 6, and 9 weeks after CCI. At 12 weeks post-injury, a final round of behavior experiments was conducted to assess motor and cognitive function, in addition to depressive-like behaviors. After completion of all behavioral tests, ipsilateral brain tissue was collected at 16 weeks post-injury and processed for NanoString analysis, flow cytometry assays, and histological outcome measures.

#### Study 2

To assess whether autophagy enhancer mitigates age-exacerbated functional recovery after TBI, aged C57BL/6 mice were fed trehalose or sucrose as control starting on the date of the injury until the time of sacrifice. Based on prior studies [51, 52, 74] and our pilot data, a single dose of 5% trehalose (Sigma-Aldrich, Cat# T9449) or 5% sucrose was injected subcutaneously into each mouse immediately after CCI surgery. Five percent trehalose or sucrose was also administered into their drinking water for the first 7 days followed by continuous administration of trehalose or sucrose solution at 2.5% of concentration in their drinking water. Trehalose and sucrose stocks were made fresh on a weekly basis using autoclaved water provided by our animal facility. Previous reports using this concentration demonstrated enhancement in autophagic function associated with neuroprotection in aging and disease [30, 39, 41]. These studies reported no effect on body weight or obvious adverse effects in WT mice [34, 62, 68, 76]. The OF, YM, CW, GS, and rotarod tests were performed starting at 4 days before CCI and repeatedly at 2 and 4 weeks after CCI. At 6 weeks post-injury, a final round of behavior experiments was conducted to fully assess the effects of trehalose on functional recovery after TBI. After completion of all behavioral tests, ipsilateral brain tissue was collected at 9 weeks post-injury and processed for flow cytometry assays.

### Neurological behavioral tests

The following behavior tests were performed with mice and group information blinded to the operators. To minimize stress and fatigue, each test was performed on a different day.

#### Motor function

##### Open field (OF) test

Spontaneous locomotor activity was examined in the OF apparatus [72]. Each test subject mouse was individually placed in a corner while facing towards the chamber wall of the apparatus (22.5 × 22.5 cm). The mice were allowed to freely explore the chambers for 10 min. Parameters on the distance travelled, average speed, time immobile, and percentage of time spent in the center of the chamber were recorded by the computer-based ANY-maze automated video-tracking system (Stoelting Co).

##### Gait analysis with catwalk XT (CW)

Analysis of gait and posture was performed with the catwalk XT automated system as mentioned in our previous publications (Noldus; RRID:SCR_004074) [43, 57, 58]. Acquisition of data took place in a darkened room with red light. A single researcher blinded to the grouping of each mouse was tasked with handling the subjects. The catwalk apparatus records print position, gait postures, and weight distribution through its green illuminated walkway. A minimum of 3 valid runs, complete crossings with no turns or scaling of sidewalls, were obtained for each tested mouse. Runs that did not comply to the preset standards were excluded from the final analysis.

##### Rotarod test

The accelerating rotarod was used to assess locomotor function and coordination at baseline and various timepoints post-injury [11]. The mouse was placed on a rotarod device (IITC Life Science, Inc.), and their latency to falling off the accelerating rotarod was recorded. The acceleration settings for the device were 4 to 40 rpm over 90 s, with each trial lasting for a maximum of 300 s. Individual scores from three trials were averaged and evaluated relative to their baseline latencies.

##### Grip strength (GS) test

Grip strength was measured using a digital grip strength meter (Bioseb BP, In Vivo Research Instruments, France), as previously described [27]. Forelimb grip strength was measured from the mouse using both the ipsilateral and contralateral forepaws together. The mouse was held by its tail, the forelimbs were placed on the grasping metal wire grid, and the mouse gripped the wire grid attached to the force transducer. Once the grip was secured, the animal was slowly pulled away from the bar. The maximal average force exerted on the grip strength meter by both forepaws was averaged from 10 trials per day for each mouse at each timepoint.

#### Cognitive function

##### Y-maze (YM) test

YM was used to assess the hippocampus-dependent spatial working memory of mice as described previously [57, 58]. The percentage of spontaneous alterations was calculated using the following equation: total alternations × 100/(total arm entries—2). If a mouse had a percentage of alternation above 50% (the chance level for randomly choosing the unfamiliar arm), it was indicative of a functional spatial memory.

##### Novel object recognition (NOR)

For testing non-hippocampal mediated memory, mice underwent NOR according to procedures described in previous studies [57, 58]. Mice were tested in an open field apparatus after a 5-min habituation period on the first day. The time spent with two identical objects was recorded on the second day of testing, and one of the familiar objects was switched out with a novel object on the third day. Testing stopped after each mouse went through a sum total of 30-s exploration time. Since mice would inherently prefer to explore novel objects, a preference for the novel object with an exploration time of more than 15 s was considered as having intact learning and memory skills.

#### Depressive-like behaviors

##### Novelty-suppressed feeding (NSF) test

The NSF was performed on subject mice to assess a rodent’s aversion to eating in a novel environment. After fasting for 24 h, mice were placed in an open field apparatus designed to model the introduction of a novel environment. Several food pellets were placed in the center of the apparatus and latency time for each mouse to reach the food and bite into it was recorded. If the mouse failed to eat within the maximum time of 10 min, a latency time of 600 s was recorded for this subject.

##### Social recognition (SR) test

This task is based on rodent’s innate tendency to investigate a novel congener over a familiar one [46, 57, 58]. Using a three-chambered rectangular apparatus made of Plexiglas with each one at equal size (20 × 40 × 23 cm). An opening between the walls allows for free access to each chamber, which contains two identical wire mesh cup containers. Before testing, each mouse was single housed overnight. On the first day, the tested mice were placed in the apparatus with two empty cups for a 10-min habituation period. On the second day, a stranger mouse was introduced and randomly placed inside one of the empty cups in either the left- or right-side chamber while the other cup was left empty. The tested mouse started from the middle chamber and allowed to freely explore all three chambers for an exploration period of 10 min. Afterwards, a second unfamiliar, stranger was placed inside the previously empty cup. The test subject was once again allowed to freely explore all three chambers for a period of 10 min. Exploration time that the subject mice spent with each cup versus stranger mouse was recorded. Since a socially functional mouse would naturally seek out unfamiliar mice for interaction, the test subject was considered capable of social recognition if index for novel mouse scored higher than 50%.

#### Thermal stimulation

##### Hot plate (HP) test

To test pain sensitivity of the hindpaws, mice were placed on the contact probe of computerized thermal stimulator on an Incremental Hot/Cold Plate Analgesia Meter (PE34, IITC Life Science, Woodland Hills, CA). The temperature was increased from 30 to 50 °C with the incremental rate at 10 °C per minute. When the tested mouse licked either one of its hindpaws, the test was stopped, and the threshold temperature was recorded. The test was conducted twice with the interval of 3 h [73].

### RNA extraction and qPCR

Following completion of all behavioral tests, mice were first euthanized with Euthasol (0.1 mL/mouse) and then transcardially perfused with 40 mL ice-cold saline. RNA samples were obtained from the ipsilateral cerebral cortex surrounding the injury site and the ipsilateral hippocampus. Total RNA was extracted from flash frozen tissue samples using a cordless motorized homogenizer with RNAse-free pellet pestles (FisherBrand) followed by the miRNeasy Mini Kit (Qiagen, Cat# 74104). Complementary DNA (cDNA) was synthesized with the Verso cDNA RT kit (Thermo Scientific, Cat# AB1453B). Both kits were used according to the manufacturer’s instructions included in the kit box. Quantitative PCR for all target RNAs (see Supplemental Table S1) was performed with the TaqMan Gene Expression assay kit (Applied Biosystems). Each sample was run in duplicates with 3 stages of 40 cycles, 2 min at 50 °C, 10 s at 95 °C (denaturing step) followed by a final transcription step of 1 min at 60 °C. Gene expression was normalized by the transcription counts of *GAPDH* and final relative expression levels were calculated with the 2^–ΔΔCt^ method [36, 37].

### NanoString neuroinflammation panel analysis

Isolated RNA was eluted in a 40 µL volume and tested using an Agilent 2100 Bioanalyzer to ensure it met specifications for purity (RNA integrity number ≥ 9) and concentration (≥ 12.5 ng/µl). Total RNA (20 ng/ul) was run on a NanoString nCounter system using the Mouse Neuroinflammation v1.0 panel (NanoString Technologies, Seattle, WA) to profile RNA transcript counts for 757 genes and 13 housekeeping genes [36, 37]. The transcription counts were normalized prior to downstream analysis and pairwise differential expression analysis with the NanoString nSolver software Version 4.0. All statistical analysis of NanoString data was performed in R language software RStudio Version 1.2.5033. Principal component analysis (PCA) was performed with the command “prcomp” and a Euclidean distance measurement method was used for clustering. Three-dimensional plotting of the PCA was performed with the pca.3d package in RStudio. All pairwise comparisons of “A vs. B” should be interpreted as “A relative to B” in the text and figures. Volcano plots depicting the fold change and *p* value of the genes were performed with the EnhancedVolcano package in R. Differentially expressed genes with a raw *p* value of equal to or less than 0.01 were further grouped into subsets based on their annotations provided by NanoString and plotted into heatmaps with the ComplexHeatmap package in R.

### Lesion volume, immunohistochemistry (IHC), and quantification

A subset of tested mice was perfused intracardially with normal saline followed by 4% paraformaldehyde. The brain was extracted and embedded in Tissue-Tek OCT compound (Sakura, Cat# 4583). Serial sections of 20-μm and 60-μm thicknesses were placed on Superfrost Plus slides (ThermoFisher, Cat# 4951PLUS). Lesion volume was measured on 60-μm coronal sections that were stained with cresyl violet (FD NeuroTechnologies, Cat# PS102-02). Quantification of the lesion volume was performed with the Stereoinvestigator software (MBF Biosciences). By outlining the missing tissue on the injured hemisphere, the software was able to estimate lesion volume with the Cavalieri method at a grid spacing of 0.1 mm [57].

Immunofluorescence imaging was performed on 20-μm coronal brain sections at around − 1.70 to 1.90-mm from bregma using standard immunostaining protocol, as described previously [37, 72]. Briefly, sections were blocked with 5% goat or guinea pig serum containing 0.3% Triton X-100. After incubation with primary and secondary antibodies (see Table S1), sections were counterstained with 4′,6-diamidino-2-phenylinodole (DAPI, Sigma-Aldrich, Cat# MBD0015) and mounted with glass coverslips using an anti-fade Hydromount solution (National Diagnostics, Cat# HS106100ML). Images from the perilesional cortex (*n* = 6 sections/location/mouse for 5–6 mice/group) were acquired using a fluorescent Nikon Ti-E inverted microscope, at 20 × (CFI Plan APO VC 20X NA 0.75 WD 1 mm) magnification, and the background of each image was subtracted using background ROI [35, 38]. The number of NeuN^+^, LC3^+^, p62^+^, and Iba-1^+^ cells was normalized to the total imaged area (mm^2^) using the NIH Image J software (1.43; NIH). Myelin images were acquired using a Leica TCS SP5 II Tunable Spectral Confocal microscope system (Leica Microsystems, Bannockburn, IL). All IHC staining experiments were performed with appropriate positive control tissue, as well as primary/secondary only negative controls.

### Flow cytometry and *ex vivo* functional assays

Mice were perfused with 40 mL of cold saline, and the ipsilateral (i.e., craniotomy-side) hemisphere was isolated [37, 57]. The olfactory bulb and cerebellum were removed, brains were halved along the interhemispheric fissure, and the ipsilateral hemisphere was placed separately in complete Roswell Park Memorial Institute (RPMI) 1640 (Invitrogen, Cat# 22400105) medium and mechanically and enzymatically digested in collagenase/dispase (1 mg/ml, Roche Diagnostics, Cat# 10269638,001), papain (5U/ml, Worthington Biochemical, Cat# LS003119), 0.5 M EDTA (1:1000, Invitrogen, Cat# 15575020), and DNAse I (10 mg/ml, Roche Diagnostics, Cat# 10104159001) for 1 h at 37 °C on a shaking incubator (200 rpm). The cell suspension was washed twice with RPMI, filtered through a 70-μm filter, and RPMI was added to a final volume of 5 mL/hemisphere and kept on ice. Cells were then transferred into FACS tubes and washed with FACS buffer. Cells were then incubated with TruStain FcX Block (Biolegend, Cat# 101320), for 10 min on ice, and stained for the following surface antigens (see Table S1): CD45-eF450 (eBioscience, Cat# 48–0451-82), CD11b-APCeF780 (eBioscience, Cat# 47–0112-82), Ly6C-AF700 (Biolegend, Cat# 128024), and MHCI-PECy7 (Biolegend, Cat# 114616). The fixable viability dye Zombie Aqua was used for live/dead discrimination (Biolegend, Cat# 423102). Cells were then washed in FACS buffer, fixed in 2% paraformaldehyde for 10 min, and washed once more prior to adding 500 ul FACS buffer. Intracellular staining for Ki67-PECy7 (Biolegend, Cat# 652426), PCNA-AF647 (Biolegend, Cat# 307912), CD68-PerCPCy5.5 (Biolegend, Cat# 137010), NeuN-PE (Millipore Sigma, Cat# FCMAB317PE), Vglut1-APC (StressMarq, Cat# SMC-394D-APC), Lamp1-PerCPCy5.5 (Biolegend, Cat# 121626), Lamp2-PE (Biolegend, Cat# 108506), Sqstm1/p62-AF647 (Novus Biologicals, Cat# NBP1-42822AF647), ATG5-AF647 (Biolegend, Cat# 847410), ATG7-AF700 (R&D Systems, Cat# FAB6608N), Ubiquitin-AF647 (Biolegend, Cat# 838710), H3-AF647 (Cell Signaling Technology, Cat# 12230S), Acetylated (Ac) Lysine (Lys)-PECy7 (Biolegend, Cat# 623408), H3-Ac-Lys9-AF488 (Cell Signaling Technology, Cat# 9683S), H3-Ac-Lys18-AF488 (Cell Signaling Technology, Cat# 73508S), H3-Ac-Lys27-AF647 (Cell Signaling Technology, Cat# 39030S), H3-Ac-Lys36-AF647 (Cell Signaling Technology, Cat# 84061S), Phospho(ser149)-H2A.X-PECy7 (Biolegend, Cat# 613420), p16-APC (StressMarq, Cat# SPC-1280D-APC), and p21-AF488 (RND-NBP2-43697AF488) was performed using Cytofix/Cytoperm Fixation/Permeabilization Kit (BD Biosciences, Cat# 554714) according to manufacturer’s instructions and as described previously [11]. Cytokine staining for TNF-PECy7 (Biolegend, Cat# 506324, and IL-1β-PerCPeF710 (eBioscience, Cat# 46–7114-82) was performed after 3-h incubation with Brefeldin A (Biolegend, Cat# 420601) at 37 °C followed by fixation/permeabilization.

The following commercially available dyes (see Table S1) were used for ex vivo cell staining according to the manufacturer’s instructions: H2dcfda (DCF, ThermoFisher, Cat# D399), LysoTracker Deep Red (ThermoFisher, Cat# L12492), Cyto-ID Autophagy Detection Kit (Enzo Life Sciences, Cat# ENZ-51031-K200), BODIPY 493/503 (ThermoFisher, Cat# D3922), Lipi-Blue (Dojindo, Cat# LD01-10), FluoroMyelin Red (ThermoFisher, Cat# F34652), FerroOrange (Dojindo, Cat# F374-12), ProteoStat Aggresome Detection Kit (Enzo Life Sciences, Cat# ENZ-51035-K100), MitoSpy Red CMXRos (Biolegend, Cat# 424802), Glucose Uptake Assay Kit (Cayman Chemical Company, Cat# 600470), BioTracker ATP-Red (Millipore Sigma, Cat# SCT045), BODIPY-Pepstatin A (ThermoFisher, Cat# P12271), and LysoLive™ Lysosomal Acid Lipase Assay Kit (Abcam, Cat# ab253380).

Data were acquired on a BD LSRFortessa cytometer using FACSDiva 6.0 (BD Biosciences) and analyzed using FlowJo (Treestar Inc.). At least 5–10 million events were collected for each sample. CountBright Absolute Counting Beads (ThermoFisher, Cat# C36950) were used to estimate cell counts per the manufacturer’s instructions. Data were expressed as counts/hemisphere. Leukocytes were first gated using a splenocyte reference (SSC-A vs FSC-A). Singlets were gated (FSC-H vs FSC-W), and live cells were gated based on Zombie Aqua exclusion (SSC-A vs Zombie Aqua-Bv510). Resident microglia were identified as the CD45^int^ CD11b^+^Ly6C^−^ population, whereas peripheral leukocytes were identified as CD45^hi^CD11b^+^ myeloid cells or CD45^hi^CD11b^−^ lymphocytes. Cell type–matched fluorescence minus one (FMO) controls were used to determine the positivity of each antibody and indicator dye [55].

### Statistical analysis

All quantitative data are plotted as mean ± standard error of mean. Statistical analysis was performed with Sigmaplot Version 12 (Systat software) or Graphpad Prism Version 4 for Windows (Graphpad Software, Inc). Kaplan–Meier survival curves were analyzed using the log-rank Mantel-Cox test. When comparing between two individual samples/groups, statistical significance was evaluated with the Student’s unpaired *t*-tests (detailed in figure legends). Comparisons within each surgery group were analyzed with 2-way ANOVA group analysis followed by multiple comparisons Dunnett’s or Tukey’s post-hoc test. For non-parametric data, the Mann Whitney test was used. A *p* value of ≤ 0.05 was considered as statistically significant.

## Results

### Old age decreases long-term survival and gait performance after TBI

To determine whether old age impacts long-term recovery after TBI, we assessed young and old male C57BL/6 mice using a battery of behavioral tests over the course of a 12-week period (Fig. 1A). Old mice showed significantly higher mortality beginning at 9 weeks as determined by the Mantel-Cox test (Fig. 1B). Body weights gradually increased in young mice and decreased in old mice in a normal age-related fashion; however, TBI-induced weight loss was evident early during the first week in the older group (Fig. 1C). To rule out any confounding effects in motor performance caused by differences in pain sensitivity, we subjected mice to the hot plate test. Both age groups displayed similar increases in pain sensitivity to heat stimuli between 3 and 6 weeks after injury (Fig. 1D).

Motor function was measured using the open field (OF) for spontaneous locomotor activity, catwalk for gait analysis, rotarod for balance and stamina, and grip testing for forelimb strength. Baseline (i.e., pre-TBI) motor function was significantly reduced with age in all tests. A significant main effect of injury was seen in rotarod performance, especially in young mice which showed decreased latency to fall at every timepoint after TBI (Fig. 1E). Forelimb grip strength was significantly reduced during the first week for both age groups but rebounded back to baseline or higher by 3 weeks (Fig. 1F). Together these data show that the effects of normal aging are more pronounced than TBI. Motor recovery was evident in both age groups for OF behavior and grip strength, while long-term deficits were seen in gait dynamics and rotarod performance.

OF results showed an injury effect in the total distance traveled and mean speed, marked by an early increase in each metric during the first week (Fig. 1G-I). This finding could not be attributed to increased anxiety-like behavior as no change in inner/outer zone preference was seen for either age (Fig. 1J). Gait analysis showed significant and lasting changes in all four limbs for all mice following injury (Supplementary Fig. S1), but given the unilateral nature of the injury, we focused on the contralateral or right-side limb dynamics. Our results revealed long-term deficits in step sequence regularity and pattern for both age groups at 12 weeks post-injury (Fig. 1K-L). Stride length was impeded early at 3 weeks in old mice and persisted up to 12 weeks (Fig. 1M). Age-related alterations in body speed were evident; however, only young mice exhibited significantly decreased body speed late after TBI (Fig. 1N). These findings suggest that the effects of old age on motor recovery are unique and complex, rather than incrementally and universally worsened relative to their younger counterparts.

### Old age exacerbates long-term cognitive dysfunction after TBI

**Fig. 2:**
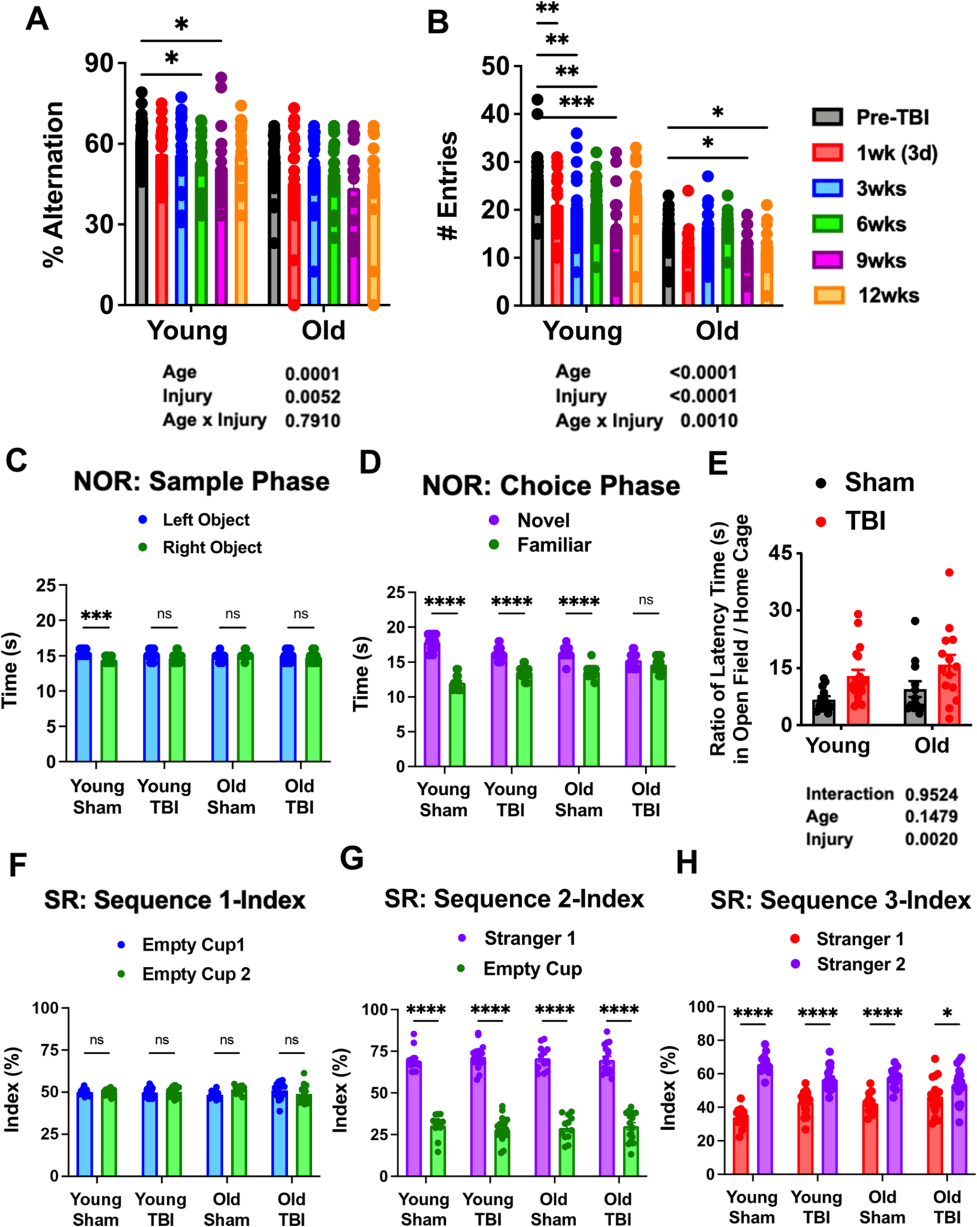
Effects of age on long-term cognition, depression, and social behavior. **A**–**B** Graph depicting percentage of alternation and total arm entry numbers for young and aged mice in baseline and repeated behavior tests. Significant effects of age and injury could be observed in both parameters observed. **C** During the sample phase of the NOR task, no differences between groups were seen in exploration time between left and right-side objects. **D** Graph depicting time spent exploring the novel versus familiar object during the choice phase of the NOR experiment. All groups except Old TBI showed significant preference for novel object over familiar object. **E** Ratio of latency time to food pellet in open field versus home cage showed injury effects for both age groups, but no aged effects were observed. **F**–**H** Graphs depicting preference index of each sequence for the social recognition experiment are shown. *N* = 13 (young sham), 12 (old sham), 19 (young TBI), and 17 (old TBI). Two-way ANOVA followed by Tukey’s post hoc test. ****p < 0.0001, ***p < 0.001, ** *p* < 0.01, * *p* < 0.05

To investigate the role of age on cognitive decline following TBI, we examined mice using Y-maze and novel object recognition (NOR) testing, to assess short-term spatial working memory and long-term recognition memory, respectively. Significant main effects of age and injury were seen in the percentage of spontaneous alternations in the Y-maze test (Fig. 2A); however, no difference was seen between pre-TBI and 12 weeks post-injury for either group. An injury-induced decrease in the number of arm entries was also seen in both age groups, with aged mice showing relatively fewer arm entries in general (Fig. 2B). NOR testing showed no clear preference in time spent between left and right objects during the sample phase, except for the young sham group (Fig. 2C). During the choice phase, a statistical preference was found for the novel object in all groups, except for the old TBI group which displayed late impairment in recognition memory (Fig. 2D). These findings indicate that old age is associated with a more pronounced loss of recognition rather than spatial memory during the chronic phase of TBI.

To assess depressive-like phenotype, we subjected mice to novelty suppressed feeding (NSF) and social interaction testing late after TBI. Given the high degree of mortality seen in the aged TBI group, we chose tests that were less stressful than those that typically measure behavioral despair. In the NSF test, a significant effect of injury, but not age, was seen in the ratio of time required for food-deprived mice to travel to the center zone of a novel versus familiar environment to obtain food (Fig. 2E). We then evaluated social behavior in chronically injured mice. No preference in time spent between left and right cups was seen in any group (Fig. 2F). However, when one cup was replaced with a stranger mouse, all groups spent significantly more time exploring the mouse (Fig. 2G). Moreover, when the other empty cup was replaced with a second stranger mouse, all groups showed a preference for the novel mouse (Fig. 2H). While an effect of age was found in time spent exploring the second stranger mouse (purple bars) in sham mice by Tukey’s post hoc test for multiple comparisons (*p* < 0.05), no statistical differences were seen between the injury groups. These data suggest that despite modest differences at baseline, depression-like behavior is prevalent in all age groups following TBI.

### Old age increases lesion volume, white matter loss, and microglial activation at 16 weeks following TBI

**Fig. 3:**
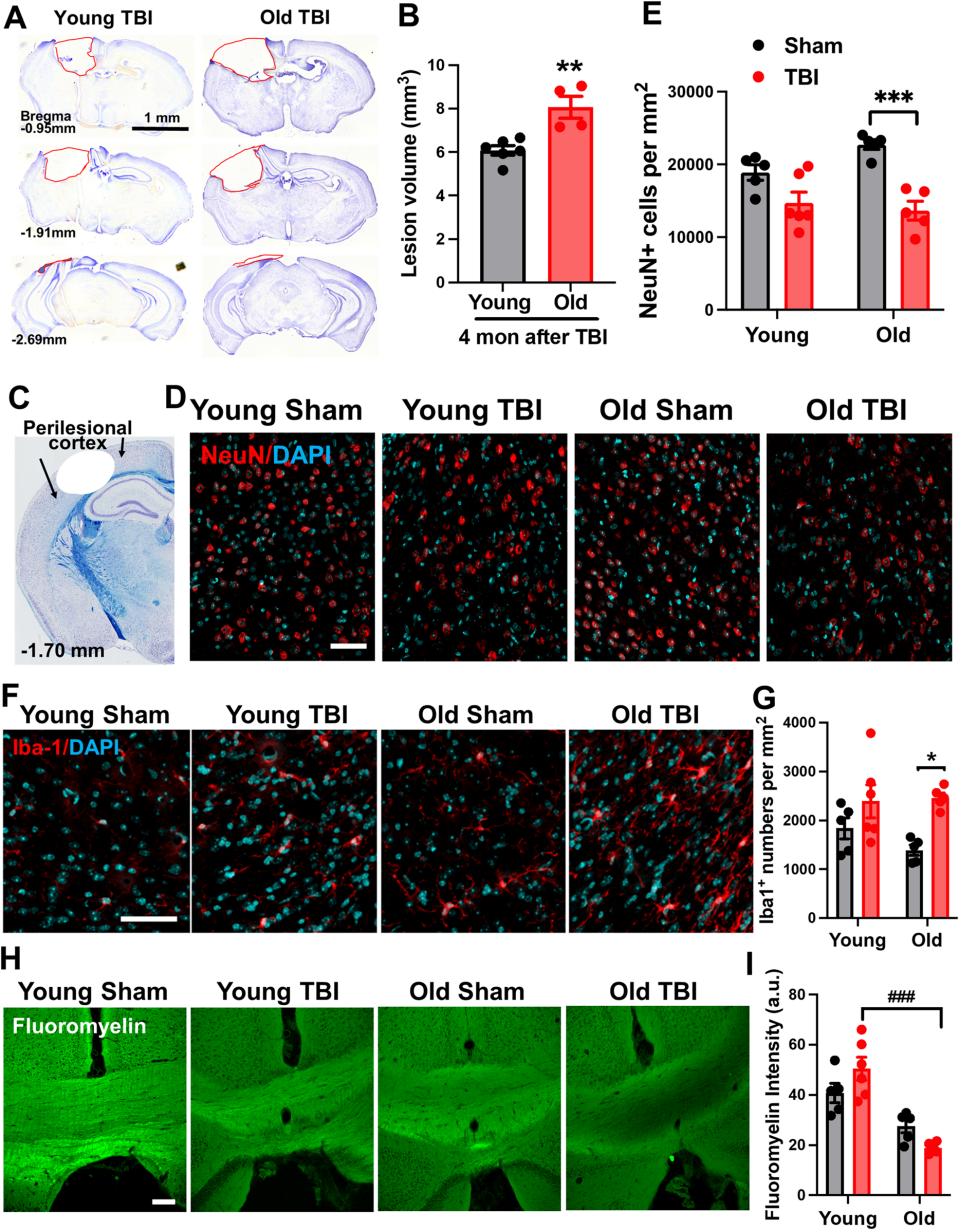
Aged mice exhibit exacerbated tissue damage, higher neuronal loss, and exaggerated white matter degradation after injury. **A** Representative images from lesion site of old TBI and young TBI mice at 16 weeks after injury. **B** Lesion volume was quantified based on the Cavalieri method of unbiased stereology using Stereologer 2000 software. *N* = 6 (young TBI) and 4 (old TBI). Unpaired *t* test, *p* < 0.01. **C** A diagram of a coronal section with arrows encompassing the perilesional cortex, adjacent to the impact site. **D** Neuronal count of NeuN-positive cells within the ipsilateral cortex showed significant neuronal loss in old TBI mice compared to old sham group. **E** Representative images of NeuN and DAPI. **F**–**G** Representative images and quantified Iba-1-positive cells. **H–I** Representative images and quantification data for FluoroMyelin staining in the medial corpus collosum. *N* = 5 (young sham), 5 (old sham), 6 (young TBI), and 5 (old TBI). Two-way ANOVA followed by Tukey’s post-hoc test. ***p < 0.001, *p < 0.05, vs old sham; ### *p* < 0.001, vs young TBI

We determined the effect of old age on chronic neurodegeneration after TBI. Quantification of cresyl violet–stained tissue sections revealed significantly larger lesion volumes in old mice compared to their younger counterparts (Fig. 3A-B). IHC of the perilesional cortex confirmed there were fewer NeuN-positive neurons in old mice at 16 weeks post-injury (Fig. 3C-E). Concomitantly, the number of Iba1-positive microglia was found to be statistically increased in the old TBI cortex, but not young, as determined by two-way ANOVA with Tukey’s multiple comparison’s test (Fig. 3F-G). Age-related pathological abnormalities were also seen in myelination of the medial corpus callosum as evidenced by FluoroMyelin stain intensity (Fig. 3H-I). Together, we demonstrate a positive association between microglia/macrophage number, severity of neuronal degeneration, and white matter degradation after chronic TBI, which is exacerbated in older mice.

### Gene pathways associated with microglia activation, phagocytosis, and autophagy are elevated with age in both the injured cortex and hippocampus

**Fig. 4:**
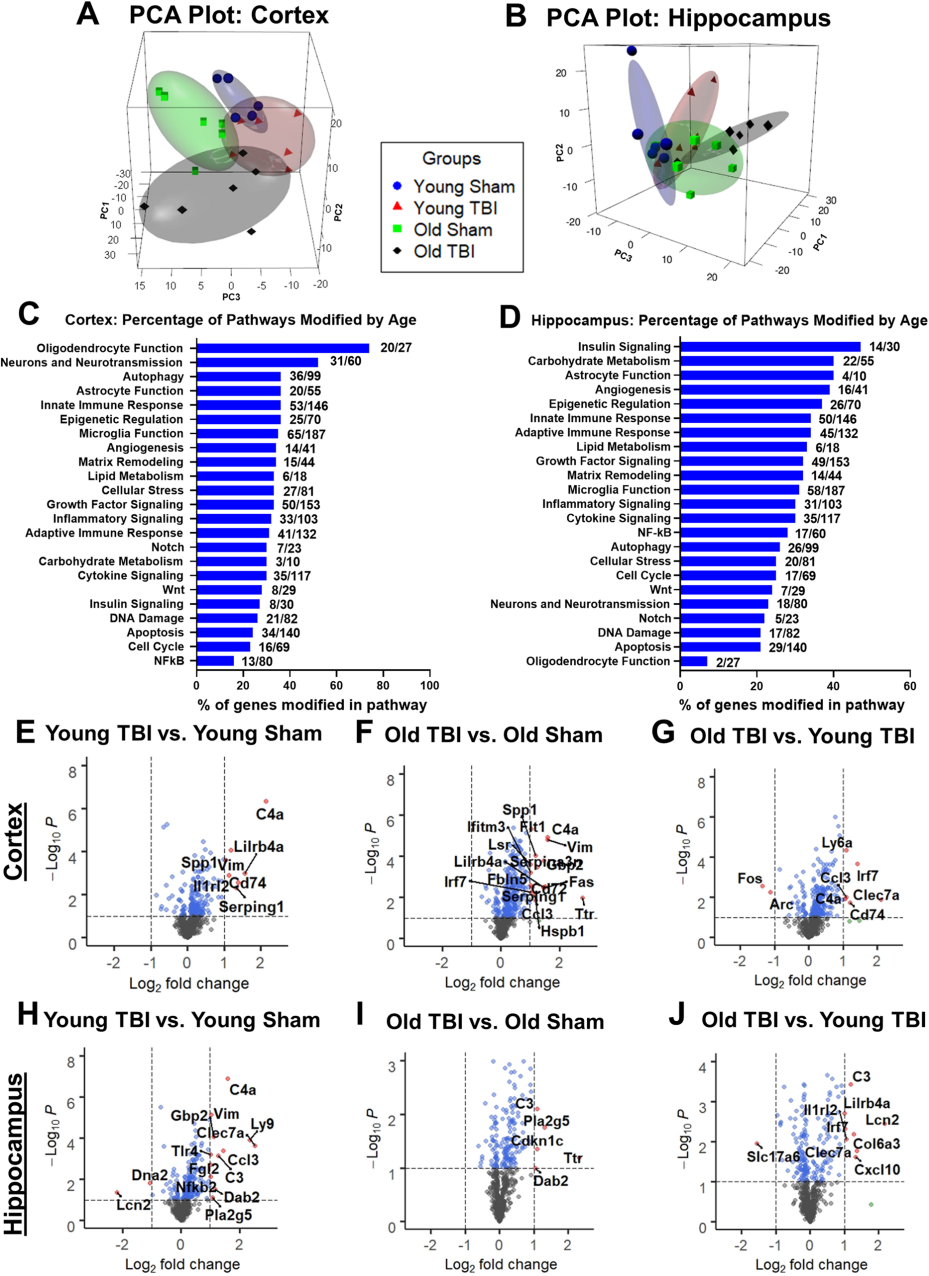
NanoString analysis of ipsilateral cerebral cortex and hippocampus at 16 weeks following TBI.** A **Principal component analysis (PCA) plot of the transcription levels of neuroinflammatory genes in the cortex. PCA was performed using all normalized gene counts from the NanoString neuroinflammation panel. The four sample groups were young sham (blue sphere), young TBI (red tetrahedron), old sham (green cube), and old TBI (black octahedron). The three main principal components of variation were captured on the x-, y-, and z-axis, respectively, showing a clear separation of clusters between the four groups. **B **PCA plot of ipsilateral hippocampus tissue samples.** C**–**D **Pathway analysis of transcriptome levels in the cortex and hippocampus based on gene annotations given by NanoString reveal high percentage of genes related to oligodendrocyte function being modified by age in the cortex, while the hippocampus showed insulin signaling as the most highly modified pathway. Volcano plot of differentially expressed genes in the **E**–**G **cortex and **H**–**J **hippocampus, demonstrating specific genes with log2 (fold change) larger than 1 and statistical significance of log10(*P*) higher than 1. *N* = 6 mice/group

To address age-related differences in the transcriptional response to TBI during the chronic phase, ipsilateral cortical and hippocampal tissues were sampled from young and old injured mice at 16 weeks post-injury. The neuroinflammation panel tested a total of 757 genes within three themes of immunity and inflammation, neurobiology and neuropathology, and metabolism and stress [36, 37]. The average transcription counts for each group after normalization with housekeeping genes were compiled in Supplementary Tables S2 and S3. The specific genes examined were listed as probe names and accession numbers. PCA of all normalized gene counts in the cortex and hippocampus revealed a distinct separation of samples into individual groups across the first two principal components (Fig. 4A–B). In the cortex, the first component accounted for 20.2% the variation across samples, the second principal component accounted for 12.9% of the variation, and a third component depicted on the z-axis accounted for 10.1% of the separation (Fig. 4A). To further analyze the connection between aging, TBI, and the behaviors reflective of cognitive decline and depression that we observed in tested mice, hippocampal tissue was also analyzed. In the hippocampus, the first component accounted for 19.2% of the variation, while the second component accounted for 15.3% and the third component accounted for 9.7% of the variation (Fig. 4B). In both regions, the old TBI samples clustered further away on the x-axis compared to the young TBI and two sham groups, perhaps reflecting the higher number of inflammatory genes that were increased in the brain of old mice at 16 weeks post-TBI.

Four pairwise comparisons of perilesional cortices and hippocampi were performed and outlined in Supplementary Fig. S2: *(1) old sham vs. young sham (set 1); (2) young TBI vs. young sham (set 2); (3) old TBI vs. old sham (set 3);* and *(4) old TBI vs. young TBI (set 4)*. TBI resulted in increased expression of genes in both young and old mice after injury, while age-related differences were seen in both baseline (old sham vs. young sham) and TBI conditions (old TBI vs. young TBI). At baseline, 130 genes (17.1%) were differentially expressed (DE) between young and old sham groups, indicating that age affected the homeostatic regulation of genes related to the inflammatory process in a fundamental way (Supplementary Fig. S2A). In the hippocampus, 121 genes were found to be DE with age (Supplementary Fig. S2B). The number of genes found to be downregulated was greater in the cortex than hippocampus *when comparing between old TBI vs. old sham, and old TBI vs. young TBI*. Venn diagrams illustrate the number of DE genes that overlap between the four different pairwise comparisons (Supplementary Fig. S2C–D). With further analysis, we were able to identify 83 genes in the cortex that were uniquely related to age and do not overlap with any genes from *young TBI vs. young sham* and *old TBI vs. old sham* (Supplementary Fig. S2C). In addition, a total of 130 genes were found to be related to TBI injury effects (Supplementary Fig. S2C). In the hippocampus, a total of 74 genes were downregulated and 47 genes were upregulated when comparing samples in old sham to young sham (Supplementary Fig. S2B). At 16 weeks after injury, comparison of young TBI vs. young sham showed as many as 126 genes were downregulated while only 21 genes were upregulated. Similarly, the comparison between old TBI vs. old sham showed a total of 110 DE genes, with 83 being downregulated in the injury group while a mere 27 genes were upregulated. As demonstrated in the adjacent Venn diagrams, we identified 100 age-related genes and 101 injury-related genes (Supplementary Fig. S2D).

**Fig. 5:**
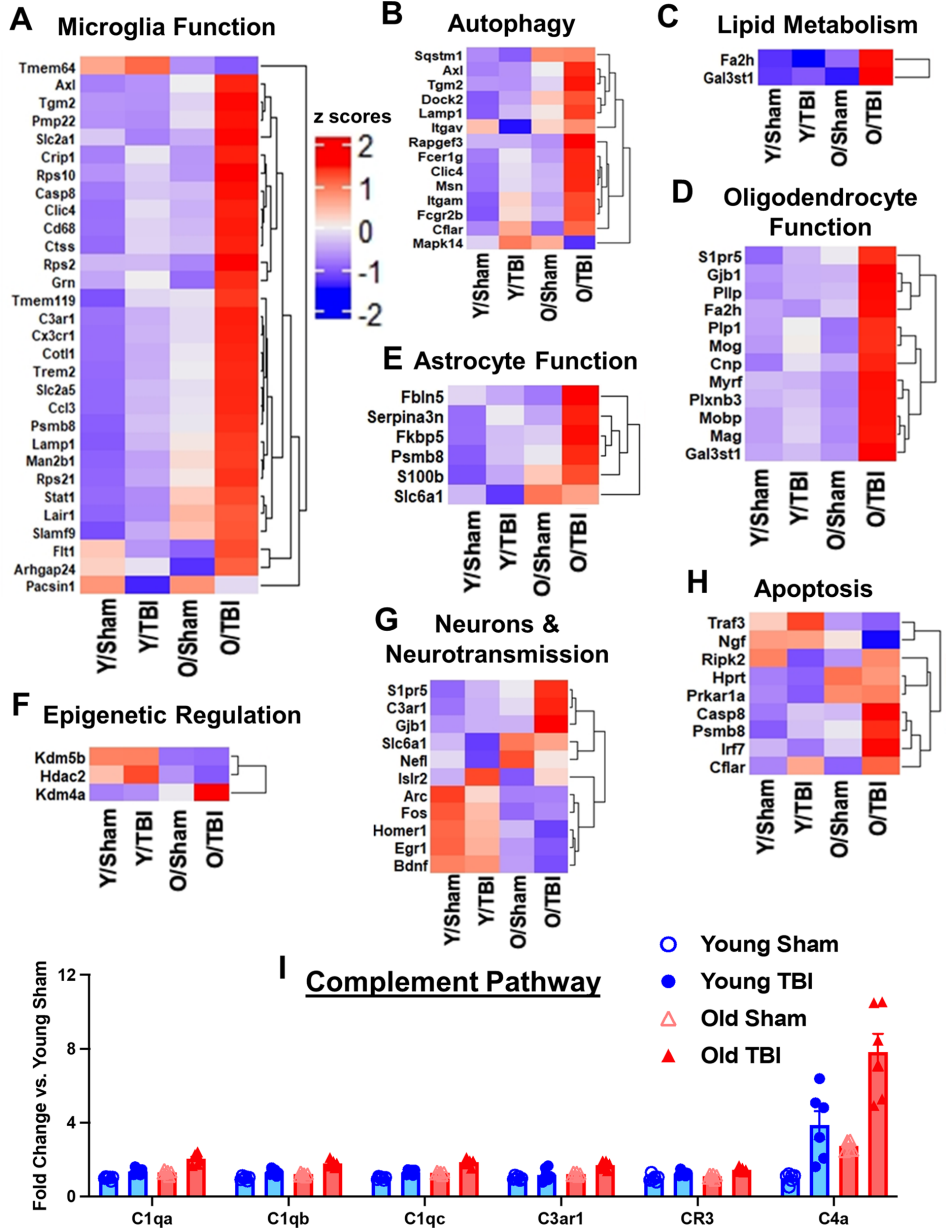
Age significantly alters neuroinflammatory profile of the injured cerebral cortex at the transcriptome level. **A–H** Heatmap of differentially expressed genes (*p* < 0.01) related to microglia function (**A**), autophagy (**B**), lipid metabolism (**C**), oligodendrocyte function (**D**), astrocyte function (**E**), epigenetic regulation (**F**), neurons and neurotransmission (**G**), and apoptosis (**H**) that were altered in differential expression analysis between old TBI and young TBI. Color coding was based on z-score scaling. **I** Bar graph of genes within the complement pathway that showed differential expression. *N* = 6 mice/group

**Fig. 6:**
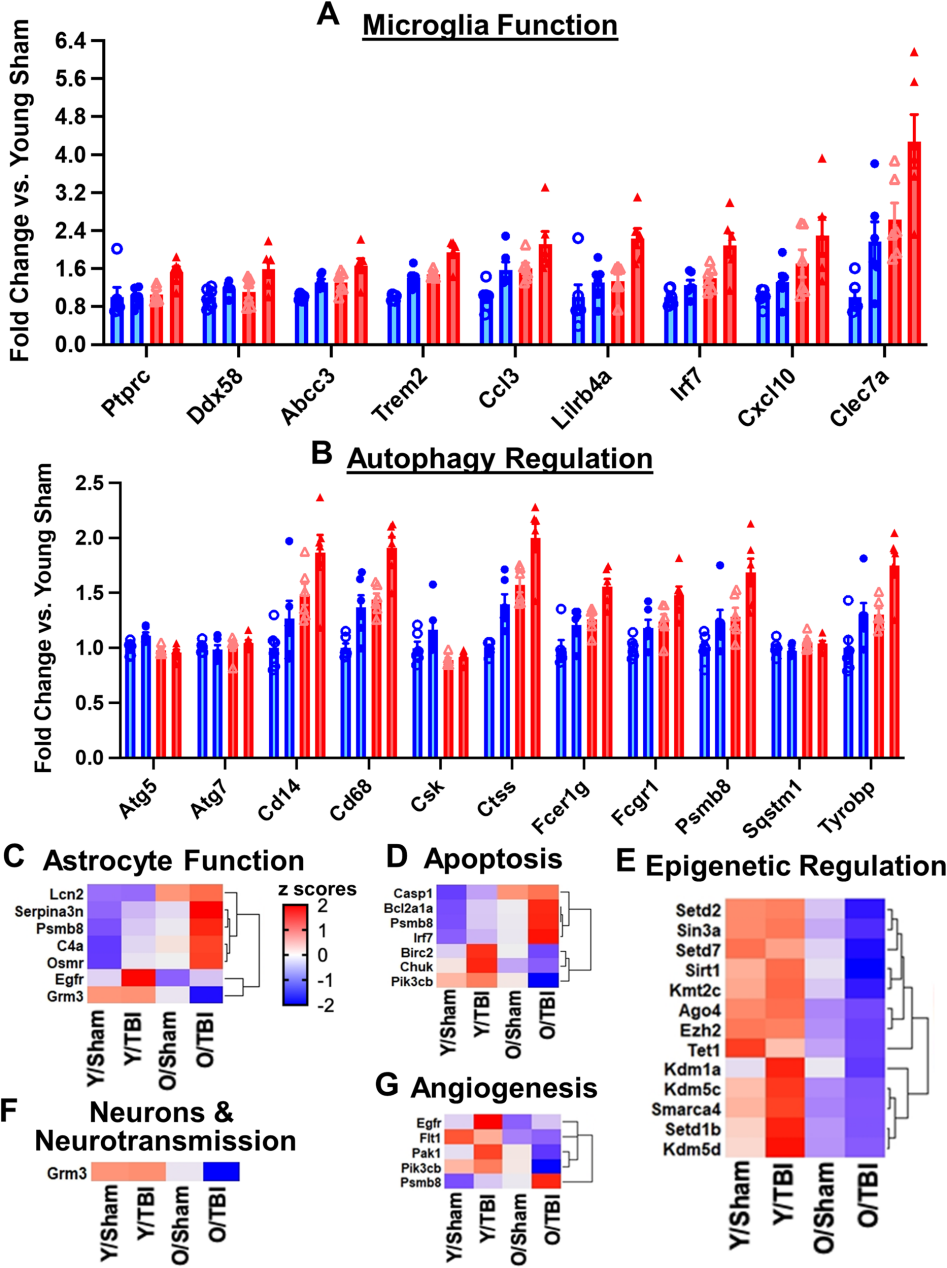
Age-related transcriptomic changes in neuroinflammatory genes of the hippocampus at 16w post-injury. **A–B** Bar graph of genes related to microglia function (**A**) and autophagy (**B**) that showed differential expression in pairwise comparisons between old TBI and young TBI. **C–G** Heatmap of differentially expressed genes (*p* < 0.01) related to astrocyte function (**C**), apoptosis (**D**), epigenetic regulation (**E**), neurons and neurotransmission (**F**), and angiogenesis (**G**) when comparing between old TBI and young TBI, i.e., genes only altered in *Set 4*. Color coding was based on z-score scaling. *N* = 6 mice/group

An initial analysis of the DE genes in each set showed several genes with large fold changes in the cortex, as illustrated in a volcano plot for each gene set (Fig. 4E–J). To further investigate the effects of normal aging on transcriptional activation, we compared the percentage of genes modified by age (union of DE genes from old sham vs. young sham and old TBI vs. young TBI) for each pathway annotation. All pathways were affected with advanced age, but genes that facilitate oligodendrocyte function, neurons and neurotransmission, autophagy, astrocyte function, innate immune response, epigenetic regulation, and microglia function were far more likely to be activated in the cortex of old sham mice compared to young sham control. Interestingly, in the hippocampus region, genes related to insulin signaling, carbohydrate metabolism, astrocyte function, angiogenesis, and epigenetic regulation were activated more significantly after injury (Fig. 4C–D). Pathway analysis of DE genes in the cortex is depicted as heatmaps (Fig. 5). A heatmap of DE genes (*p* < 0.01) implicated in each notated pathway generally show an age-related upregulation in the chronic phase of TBI (Fig. 5A–H). The complement pathway, which has been previously implicated in age-related neurodegenerative disease and synaptic maintenance, was found to be increased both with age and injury (Fig. 5I). Genes associated with microglia function and autophagy regulation were also upregulated with age and/or injury in the hippocampus (Fig. 6A–B). A heatmap of DE genes with a raw *p* value of less than 0.01 implicated in astrocyte function, apoptosis, epigenetic regulation, neurons and neurotransmission, and angiogenesis showed a more mixed expression pattern after injury (Fig. 6C–G). More specifically, the age- and chronic injury–related upregulation of *Axl*, *CD14*, *CD68*, *CD74*, *Ctss*, *Clec7a*, *Trem2*, and *Tyrobp* genes in both the cortex and hippocampus is consistent with that seen in the multiple models of age-related neurodegenerative diseases and is representative of a disease-activated microglia (DAM) signature that reflects a pathological state of the CNS [28].

As an additional measure for examining transcriptional changes, we validated several genes that showed significant changes via real-time quantitative PCR (Supplementary Figs. S3 and S4). For example, the gene *Clec7a* that encodes a glycoprotein linked to the modulation of the natural killer gene complex region and connected to the innate immune response functions. Real-time qPCR validation of this gene revealed significantly higher expression levels of *Clec7a* in the cerebral cortex of old TBI mice compared to the old sham group, but barely any change in the young TBI vs. young sham group. Taken together, our results show a diverse transcriptional response of inflammatory genes in old mice compared to young mice after TBI, with several genes showing differential expression between the old TBI and young TBI groups.

**Fig. 7:**
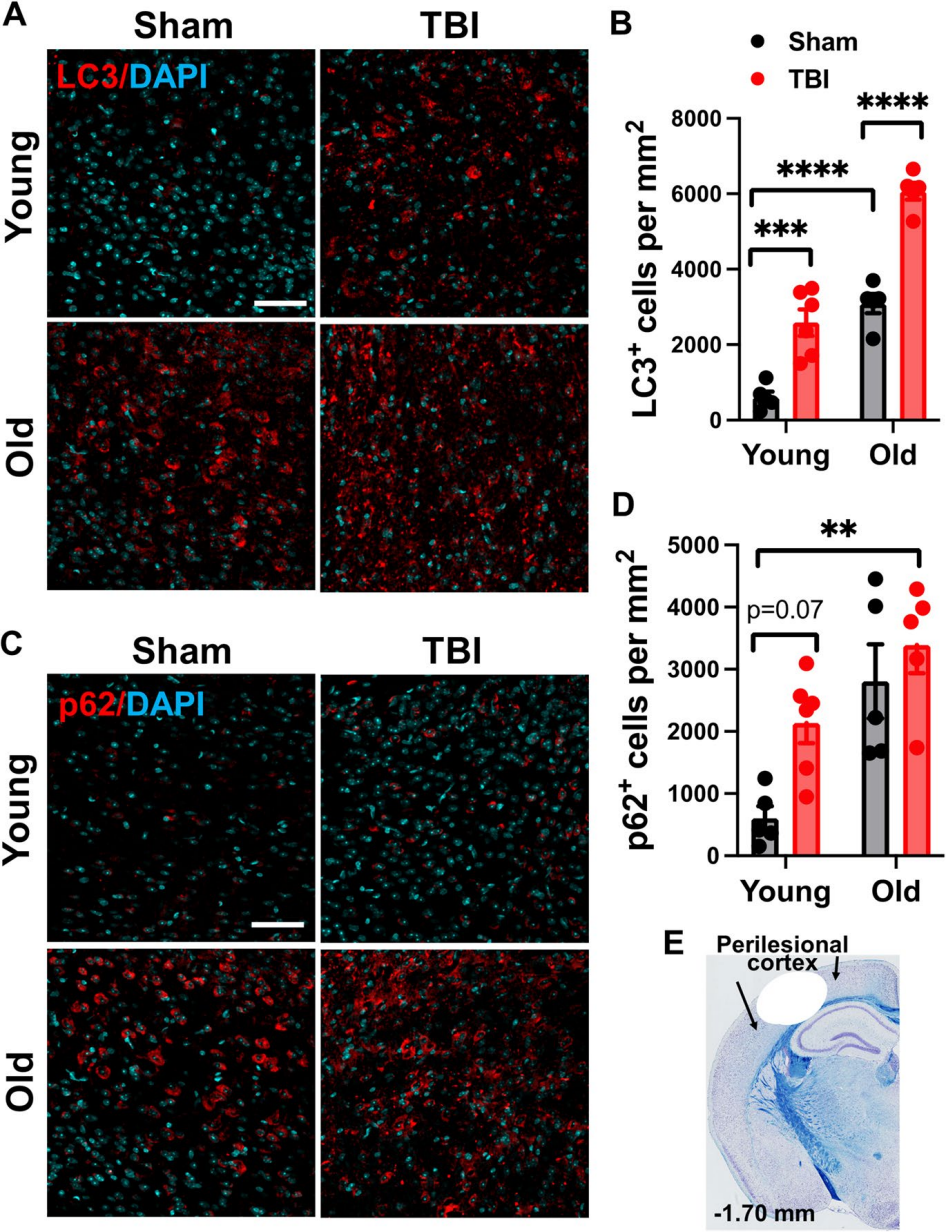
The aged TBI brain displays increased expression of autophagy markers during the chronic phase of injury. **A**–**B** Representative images and quantified data of LC3-positive cells in the ipsilateral cerebral cortex at 16 weeks post-injury. **C**–**D** Representative images and quantification of p62/SQSTM1-positive cells in the ipsilateral cerebral cortex at 16 weeks post-injury. **E** A diagram of a coronal section with arrows encompassing the region of interest (i.e., perilesional cortex), adjacent to the impact site. *N* = 5 (young sham), 5 (old sham), 6 (young TBI), and 5 (old TBI). Two-way ANOVA followed by Tukey’s post hoc test. ****p < 0.0001, ***p < 0.001, **p < 0.01

To better illustrate the relationship between neuropathology and expression of autophagic markers, we confirmed immuno-reactivity of LC3 and p62 at the protein level in the perilesional cortex (Fig. 7A–E). Significant effects of age- and TBI were seen in both the number of LC3-positive and p62-positive cells at 16 weeks post-injury, consistent with our NanoString findings. In sum, these data suggest autophagic changes in the aged and injured brain may precipitate chronic microglial activation and neuronal degeneration.

### Old age increases infiltration of lymphocytes and exaggerates microglial responses to TBI

**Fig. 8:**
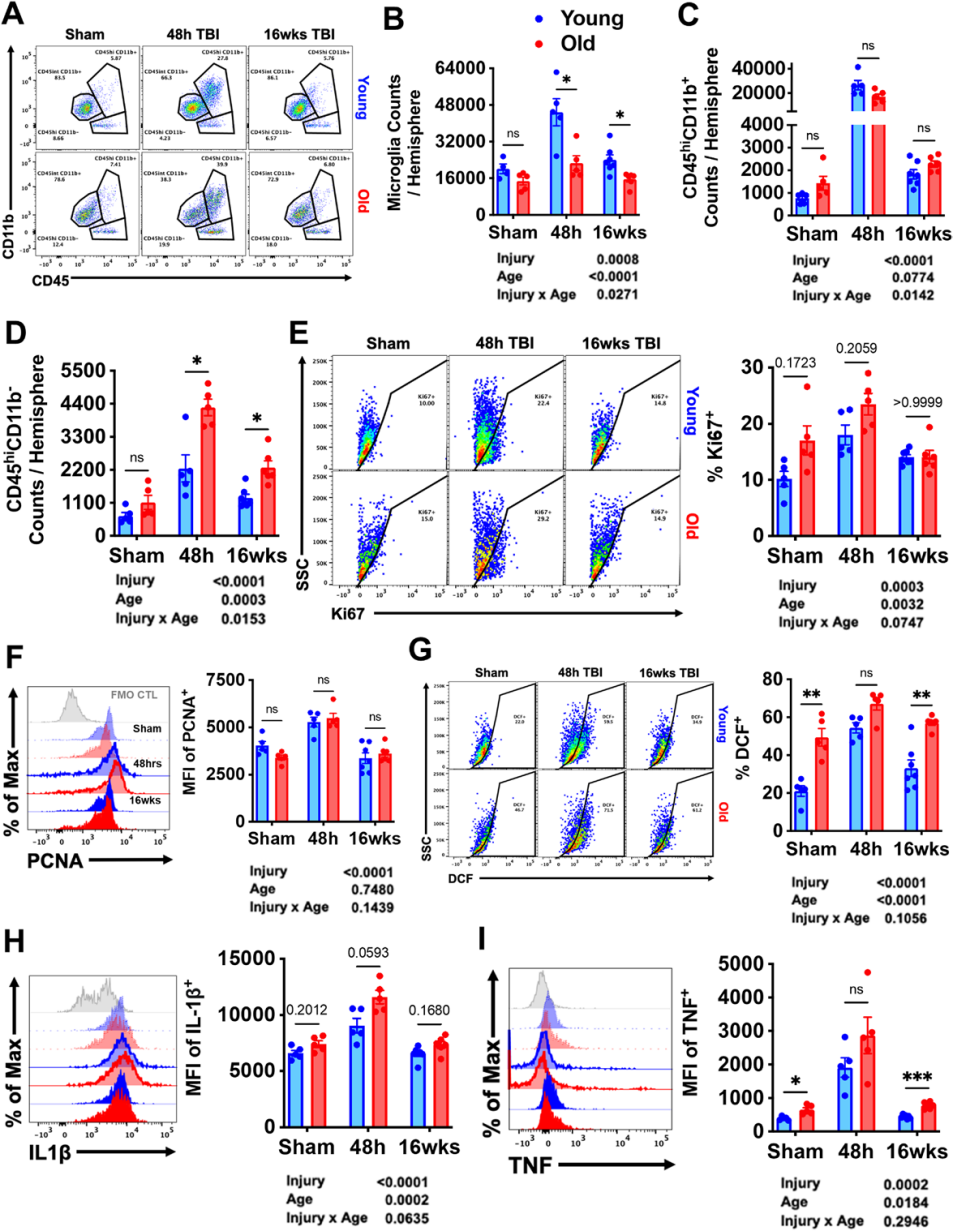
The effects of old age on the central immune response to TBI. **A** A representative dot plot of leukocyte populations in the brain at 48 h and 16 weeks after TBI. Quantification of CD45^int^CD11b^+^ microglia **(B)**, CD45^hi^CD11b^+^ myeloid cells **(C)**, and CD45^hi^CD11b^−^ putative lymphocyte **(D)** cell counts are shown. Microglial proliferation as measured using cell cycle markers. A main effect of injury was seen in Ki67 **(E)** and PCNA **(F)** expression, driven largely by the increase at 48 h after TBI. Reactive oxygen species (ROS) and oxidative stress were measured using DCF. Main effects of age and injury were seen in microglial ROS levels **(G)**. Pro-inflammatory cytokine production was assessed. The relative expression level of IL-1β **(H)** and TNF **(I)** in microglia is shown. For all histograms, gray = FMO control, blue = young, red = old, sham = no outline/no fill, 48 h TBI = bold outline/no fill, and 16w TBI = bold outline/bold fill. *N* = 5–7/group. Data were analyzed using 2-way ANOVA group analysis with Tukey’s test for multiple comparisons. ***p < 0.001, **p < 0.01, *p < 0.05

Next, we investigated the cellular inflammatory response at early (48 h) and late (16 weeks) timepoints after TBI. Brain leukocyte identification and characterization was performed using flow cytometry. Old age was associated with fewer microglia (CD45^int^CD11b^+^Ly6C^−^) and less robust microglial accumulation in terms of absolute number (Fig. 8A–B). Normal age-related reductions in microglia number have been reported previously by our group and others and likely reflects age-related dystrophy and proliferative senescence [77]. The number of infiltrating myeloid cells (CD45^hi^CD11b^+^), however, was comparable between young and old groups and showed two-way ANOVA main effects of injury and age, including a significant interaction between them (Fig. 8C). Delayed infiltration and accumulation of putative lymphocyte populations (CD45^hi^CD11b^−^) was dramatically in old mice at all timepoints post-injury (Fig. 8D). These findings highlight the profound effects of aging on the central and peripheral immune response to TBI and suggests that blood–brain barrier integrity may be uniquely compromised in the aged brain long after injury.

To address the rapidly shifting dynamics of microglia proliferation that occurs during the first week of injury, we examined two commonly used nuclear protein markers of cellular proliferation, Ki67 and proliferating cell nuclear antigen (PCNA). Both markers were significantly upregulated in microglia at 48 h after TBI, but no statistical difference between injury groups was found (Fig. 8E–F). Despite being fewer in number, microglia in old sham mice showed significantly higher basal expression of Ki67, indicating active cell cycling. The percentage of Ki67-positive microglia remained elevated late after TBI in young but not old microglia as determined by Dunnett’s test (*p* = 0.05). These data do not indicate an age-related difference in the kinetic progression of microglial proliferation at 48 h; however, during the chronic stages of injury microglia from old TBI, mice showed reduced proliferative capacity relative to sham compared to those from the young TBI group.

We then examined the activation state of microglia based on light scatter properties and ex vivo functional analysis. Significant effects of age and injury were seen microglia size and granularity at all timepoints, as measured by forward and side scatter intensity, respectively (Supplementary Fig. S5A–B). Old microglia became markedly more granular after 48-h post-injury compared to young microglia, suggesting increased inflammatory activation. Reactive oxygen species (ROS) production is associated with oxidative stress which can exacerbate neurodegeneration [32, 57]. Old microglia had dramatically higher ROS production at baseline and at all timepoints post-injury, whereas young microglia exhibited a more robust increase in ROS relative to its respective sham control (Fig. 8G). The percentage of microglia producing detectable ROS remained elevated at 16 weeks in young mice as determined by Dunnett’s test. Lastly, we measured cytokine production in microglia which showed significant effects of both age and injury in expression level of IL1β and TNF protein (Fig. 8H–I). Both cytokines showed an age-related increase during the acute phase of TBI, which largely abated by 16 weeks (*p* < 0.05). Taken together, our data imply that old age primes microglia to become more reactive to acute TBI, consistent with previous work. Independent of any change in cell number, basic features of microglial activation during the chronic phase of TBI appear to be more subtle on a per cell basis.

### Microglial phagocytosis of neurons, expression of autophagy markers, and lipofuscin content are increased with age and injury

**Fig. 9:**
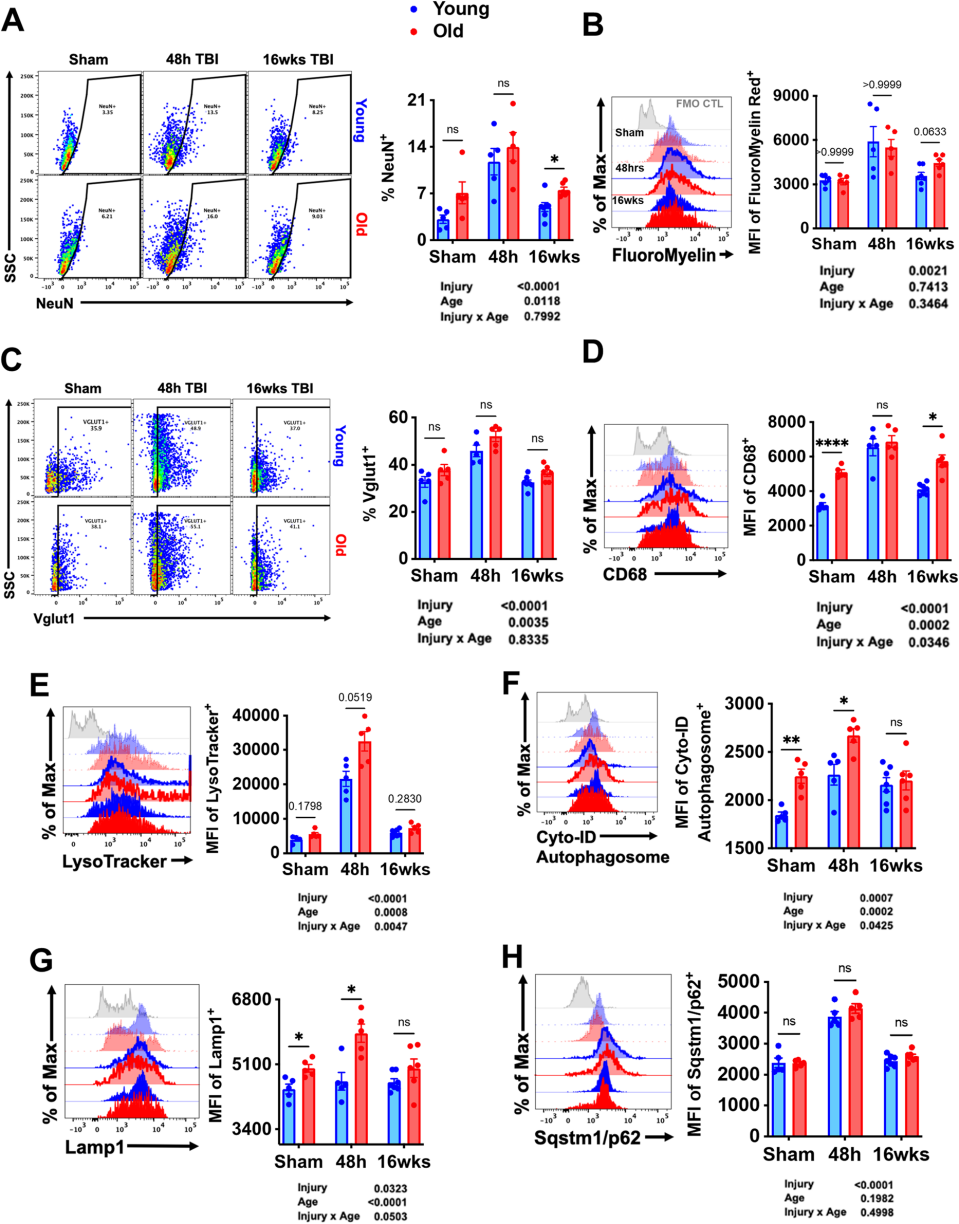
Microglial phagocytosis of neurons, expression of autophagy markers, and lipofuscin content is chronically increased with age and injury. Phagocytosis was assessed by intracellular detection of neuronal and myelin antigens. **A** The percentage of NeuN-positive microglia was significantly increased with age and injury. The mean fluorescence intensity of FluoroMyelin red staining was acutely increased in microglia from both age groups **(B)**. The presence of vesicular glutamate transporter 1 (vGlut1) **(C)** and the phagosome marker CD68 **(D**) were increased in microglia with both old age and TBI. Representative histograms illustrate the relative abundance of lysosomes and LC3II-positive autophagosomes in microglia as measured by LysoTracker **(E)** and Cyto-ID Autophagosome dyes **(F)**. For all histograms, gray = FMO control, blue = young, red = old, sham = no outline/no fill, 48 h TBI = bold outline/no fill, and 12w TBI = bold outline/bold fill. The mean fluorescence intensity of Lamp1 **(G)** and Sqstm1/p62 **(H)** for microglia are shown. *N* = 5–7/group. Data were analyzed using 2-way ANOVA group analysis with Tukey’s test for multiple comparisons. ****p < 0.0001, **p < 0.01, *p < 0.05

The emergence of a DAM signature led us to surmise that age-related deficits in autophagy function underlie chronic microglial activation and dysfunction following TBI. To better understand the temporal effects of TBI on microglia function, we performed an extensive characterization of phagocytosis and autophagy informed largely by our NanoString results indicating age-related increases in these specific pathways late after injury. Phagocytosis was measured by intracellular detection of neuronal antigens, myelin content, and the lysosomal/endosomal membrane marker CD68. Significant main effects of both age and injury were seen in the percentage of NeuN-positive microglia (Fig. 9A). The frequency of NeuN-positive microglia increased sharply at 48 h in both age groups and remained high at 16 weeks in old mice. Similar changes were seen in intracellular myelin content; however, while levels trended higher in old mice at 16 weeks (*p* = 0.06) it did not meet statistical significance in the two-way ANOVA multiple comparisons test (Fig. 9B). Intracellular detection of vesicular glutamate transporter 1 (Vglut1), a synaptic protein in neurons, was also increased acutely in microglia after TBI (Fig. 9C). A main effect of age was found, indicating a higher presence of this synaptic marker in old microglia. The phagocytosis marker CD68 was significantly higher in old microglia both at baseline and at 16 weeks but was elevated to a similar extent at 48 h in both age groups (Fig. 9D). These findings suggest that microglial phagocytosis of dead or dying neurons is robust in the early stages of TBI, and modestly increased with both age and time post-injury, consistent with our gene expression data.

Lysosome and autophagosome content in microglia were significantly increased with both age and injury and remained elevated for up to 16 weeks (Fig. 9E–G). Protein expression of Sqstm1/p62 was increased at 48 h, but no effect of age was seen (Fig. 9H). Defects in the autophagy pathway can result in the cytosolic and lysosomal accumulation of an autofluorescent amalgam of oxidized lipids, proteins, and metals known as lipofuscin. Main effects of age and injury were seen in intracellular lipid content, iron levels, peptide/protein aggregation, and autofluorescence (Supplementary Fig. S5C–F). Neutral lipid and iron content increased sharply at 48 h, consistent with the increase in phagocytic activity. Protein aggregation and autofluorescence were largely unperturbed at 48 h; however, both were found to be elevated above sham levels at 16 weeks in young microglia as determined by Dunnett’s test (*p* < 0.05 and *p* < 0.05, respectively). Collectively, these data imply that phagocytosis and autophagic function are exacerbated with age and TBI, consistent with our DAM gene signature. Although these processes are more active during the acute stages of injury, many markers, including the accumulation of lipofuscin material, show a sustained increase during the chronic phase.

### Age-related changes in microglial histone acetylation patterns, metabolic activity levels, and expression of senescence markers are altered after TBI

**Fig. 10:**
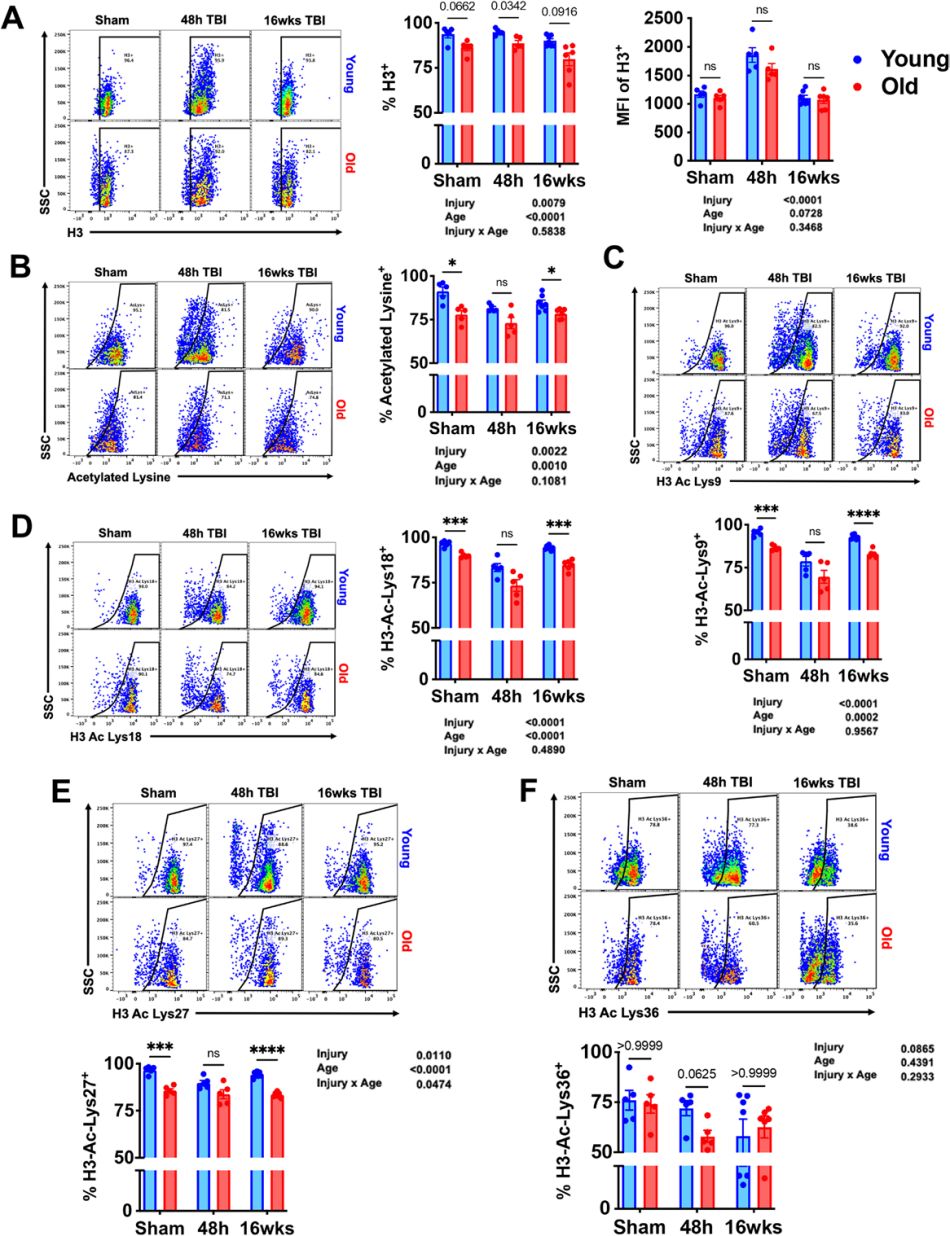
TBI exacerbates age-related alterations in histone acetylation of lysine residues in microglia. Flow cytometric analysis of epigenetic changes in microglia was performed. **A** The percentage of Histone 3 (H3)-positive microglia is shown in sham control and at 48 h and 16 weeks after TBI. The mean fluorescence intensity of H3 protein expression in microglia is also quantified. **B** Global lysine acetylation in microglia is illustrated in the representative dot plot. Acetylation frequencies of the H3 residues **C** lysine 9, **D** lysine 18, **E** lysine 27, and **F** lysine 36 were quantified. *N* = 5–7/group. Data were analyzed using 2-way ANOVA group analysis with Tukey’s test for multiple comparisons. ****p < 0.0001, ***p < 0.001, *p < 0.05

Given the widespread effects of aging on microglial function, we also evaluated changes in epigenetic regulation, metabolic activity, and senescence-like phenotype following TBI. Microglia activation is associated with increased histone deacetylase (HDAC) and sirtuin (SIRT) activity resulting in hypoacetylation of histones and non-histone proteins and increased expression of inflammatory markers [49]. Our results show an age-related loss of histone 3 (H3) within the general microglia population; however, H3 expression levels were dramatically increased at 48 h in both age groups (Fig. 10A). The percentage of microglia expressing acetylated lysine residues was significantly reduced with age and injury indirectly suggesting increased HDAC and SIRT activity (Fig. 10B). To further probe the impact of TBI on histone modification, we examined acetylation patterns on H3. Significant effects of age and injury, or injury alone, were seen in H3 hypoacetylation of the lysine residue at N-terminal positions 9, 18, 27, and 36 (Fig. 10C–F). These data suggest that aging and TBI result in the removal of acetyl groups from H3 which is known to increase chromatin condensation and decrease gene transcription.

**Fig. 11:**
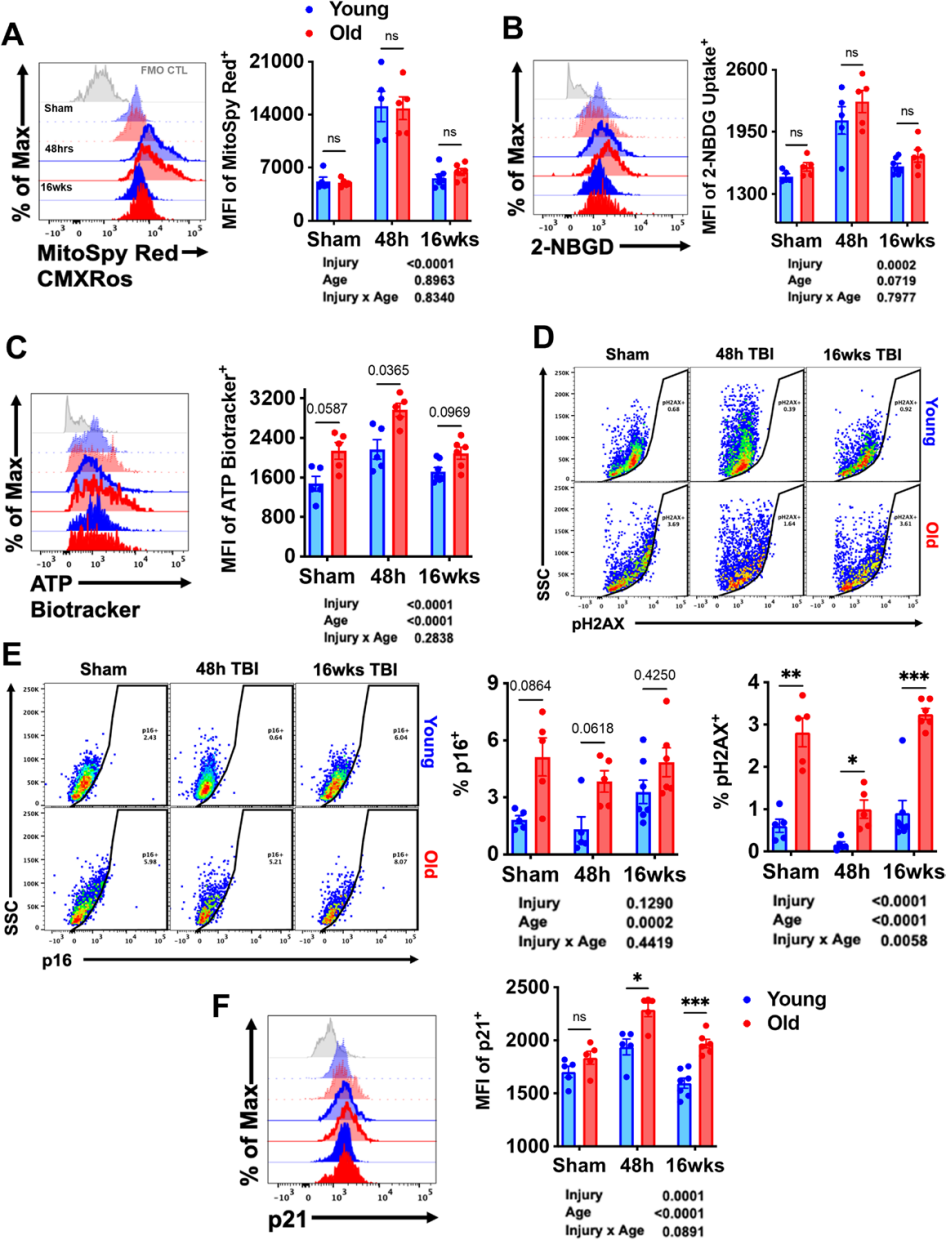
TBI exacerbates age-related alterations in metabolic activity and expression of senescence markers. Flow cytometric analysis of metabolic changes in microglia was performed. **A** Representative histogram depicts the relative mitochondrial membrane potential in microglia as measured using MitoSpy Red CMXRos. The mean fluorescence intensity of **B** glucose uptake and **C** ATP levels in microglia were assessed using 2-NBDG and ATP BioTracker, respectively. **D** A representative dot plot illustrating the percentage of microglia immunoreactive to the DNA damage sensor, phosphorylated (ser139) H2AX, is shown and quantified. The frequency of **E** p16-positive and **F** p21-positive microglia was quantified. For all histograms, gray = FMO control, blue = young, red = old, sham = no outline/no fill, 48 h TBI = bold outline/no fill, and 16w TBI = bold outline/bold fill. *N* = 5–7/group. Data were analyzed using 2-way ANOVA group analysis with Tukey’s test for multiple comparisons. ****p < 0.0001, ***p < 0.001, *p < 0.05

TBI acutely increased mitochondrial activity, glucose uptake, and ATP production in microglia (Fig. 11A–C). At 16 weeks post-injury, the cellular demand for glucose remained higher than sham control in young mice as determined by Dunnett’s test (*p* < 0.01). These findings suggest that TBI causes a metabolic crisis in microglia that is associated with a chronic increase in glucose uptake in young mice.

We have previously demonstrated that a small population of microglia upregulate markers of cellular senescence in an age- and TBI-dependent manner [56]. However, the sustained presence of a senescent-like phenotype has yet to be reported in microglia at chronic timepoints after injury. Expression of the DNA damage sensor, p-H2AX, showed a basal elevation with age (Fig. 11D). Interestingly, p-H2AX levels were significantly decreased at 48 h, likely due to a stress-mediated increase in DNA repair mechanisms. Although expression was further increased from 48 h to 16 weeks, levels did not surpass that seen in sham controls. Expression of the cell cycle arrest markers p16 and p21 were increased with normal aging (Fig. 11E–F). The percent of p16-positive microglia trended higher in young TBI mice at 16 weeks compared to sham, but significance was not achieved by two-way ANOVA multiple comparison test. It should be noted that the absolute number of p16-positive microglia may be significantly increased in the absence of a statistical change in percentage. In contrast, a robust increase in the relative expression of p21 was seen at 48 h in both age groups; however, levels were higher in old microglia at both timepoints post-injury (Fig. 11F). Together, these results indicate that aging interacts with TBI to modify key aspects of microglial homeostasis, including post-translational epigenetic regulation, metabolic function, and development of a senescence-like phenotype.

### Trehalose treatment enhances functional recovery in motor and cognitive tasks and decreases depressive-like behavior

**Fig. 12:**
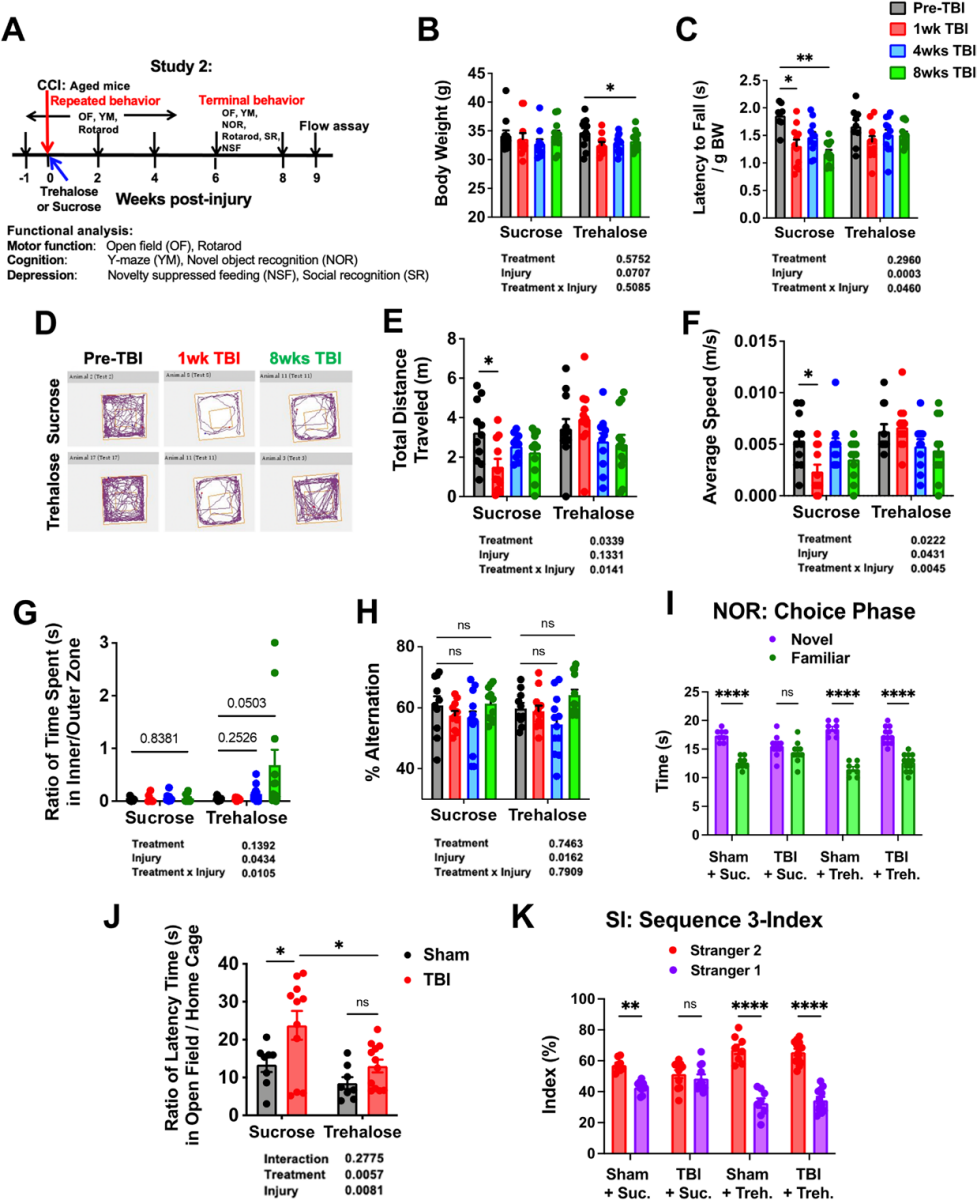
Trehalose treatment enhances functional recovery in motor and cognitive tasks and decreases depressive-like behavior. **A **Diagram illustrating timepoints by weeks pre- or post-injury and of repeated or terminal behavior experiments conducted on aged mice with trehalose or sucrose (i.e., vehicle control) treatment throughout study 2.** B **Graph depicting body weight data of aged mice that underwent continuous trehalose treatment or vehicle control at baseline and follow-up measurements up to 8 weeks post-injury. **C **Rotarod data shows decreased latency time to fall in aged mice consuming sucrose after injury; however, no injury effect could be observed in the trehalose group.** D–G **Spontaneous activity in the open field experiment showed no significant injury effects in the sucrose group for total distance travelled** (E) **and average speed **(F)**; however, no neurological deficits were observed for the trehalose group.** H **Y-maze experiment showed no treatment effect in the percentage of % alternations.** I **NOR task showed significant injury effects in the TBI sucrose group during the choice phase of NOR experiment.** J **The latency to reach food in the center of a novel arena was recorded for the NSF test. The trehalose TBI group showed significantly lower open field versus home cage ratio when compared to the sucrose TBI group. No difference was found in latency time in the home cage, confirming that the novel environment was a source of anxiety for TBI mice.** K **In sequence 3 of the social recognition experiment, the TBI sucrose group failed to show preference for the second, novel stranger mouse, which suggests neurological deficits in social interaction. *N* = 8 (sham sucrose), 8 (sham trehalose), 11 (TBI sucrose), and 12 (TBI trehalose). Two-way ANOVA followed by Tukey’s post hoc test. ****p < 0.0001, ** *p* < 0.01, * *p* < 0.05

For proof-of-principle that the identified gene pathways are functionally important to age-related TBI outcome, we treated old mice with the autophagic enhancer, trehalose. Old mice were administered trehalose or sucrose control in their drinking water beginning on d1 for up to 8 weeks after surgery. A battery of behavioral tests was performed to monitor changes in motor performance, cognitive function, and anxiety/depression (Fig. 12A). Trehalose treatment resulted in a delayed decrease in body weight (Fig. 12B). No statistical deficits were seen in rotarod performance at any timepoint in mice treated with trehalose, whereas mice treated with sucrose control exhibited lasting impairment for up to 8 weeks after TBI (Fig. 12C). OF activity was then assessed (Fig. 12D). Interestingly, sucrose-treated mice traveled significant shorter distances in the OF during the first week of injury (Fig. 12E). This was also accompanied by a decrease in speed, not seen in trehalose-treated mice (Fig. 12F). A statistical increase in time spent in the center zone of the OF late after injury was recorded in mice administered trehalose, suggesting that enhancing autophagy during TBI may result in delayed anxiolytic effects (Fig. 12G). These data suggest continued administration of trehalose may attenuate motor deficits in aged mice after TBI.

To address the effect of trehalose on long-term cognitive outcomes in old mice, we evaluated mice using the Y-maze and NOR tests. While both treatment groups showed a temporary reduction in the number of arm entries early after TBI, no therapeutic effects were seen (Fig. 12H, Supplementary Fig. S6A). However, trehalose-treated mice did show improvement in recognition memory at 8 weeks post-TBI compared to sucrose control (Fig. 12I, Supplementary Fig. S6B–C). Lastly, NSF and social interaction tests were employed to evaluate anxiety/depressive-like phenotype. Trehalose-treated mice displayed shorter latency times to reach food at the center of the OF compared to sucrose control, suggesting less depression-related behavior, consistent with our OF findings (Fig. 12J). Finally, social interaction testing showed that trehalose increased the amount of time spent interacting with a novel stranger mouse in the social discrimination phase (i.e., sequence 3) of the test (Fig. 12K, Supplementary Fig. S6D). Together, these findings show that continued trehalose treatment can preserve recognition memory and reduce anxiety/depression in old mice late after injury.

### Trehalose treatment reduces chronic brain inflammation and phagocytosis of neuronal synapses by modulating multiple steps of the autophagy pathway in microglia

**Fig. 13:**
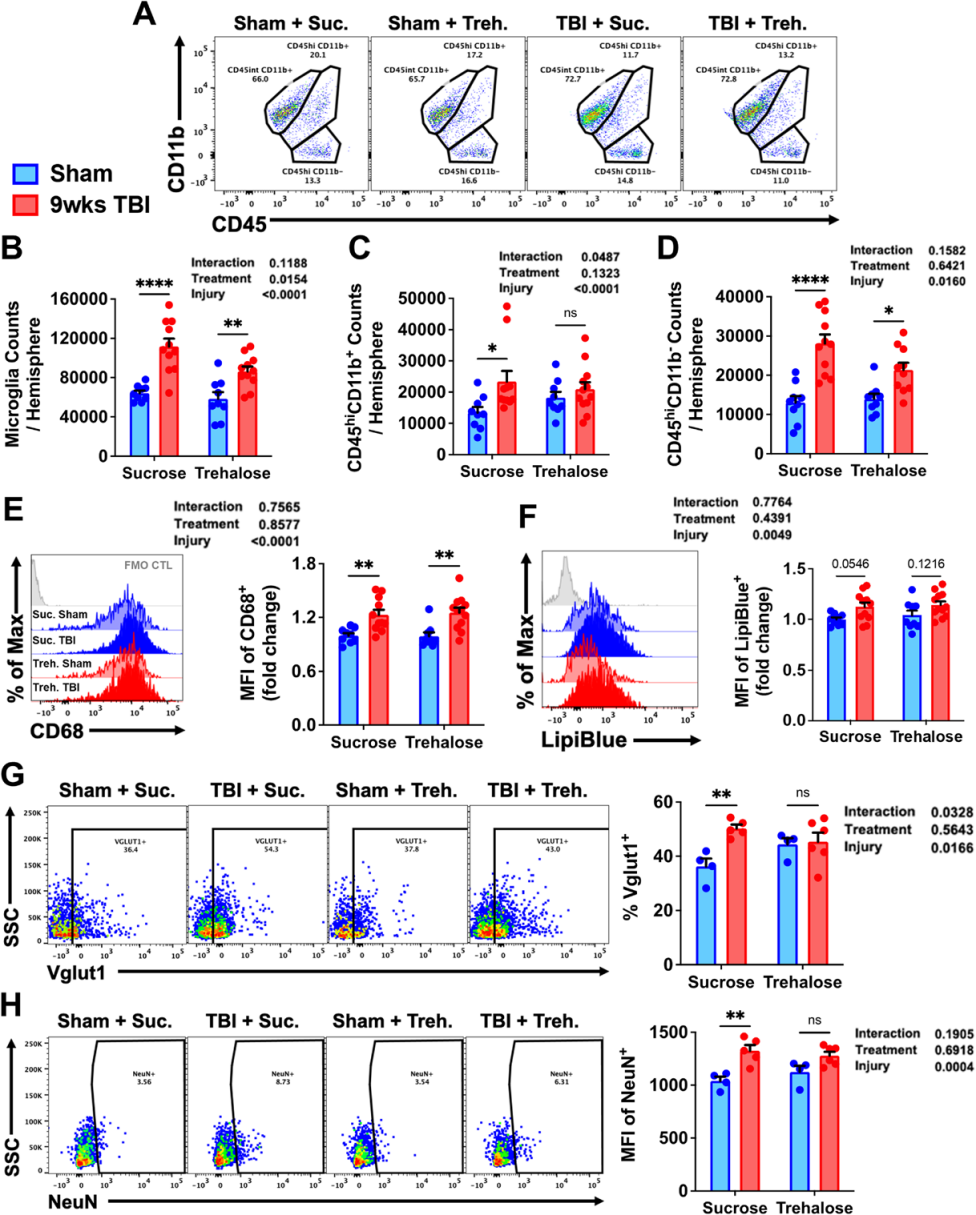
Trehalose treatment reduces long-term microgliosis, lymphocyte infiltration, and phagocytosis of neurons following TBI. **A** A representative dot plot of leukocyte populations in the brain at 9 weeks after TBI. Quantification of CD45^int^CD11b^+^ microglia **(B**), CD45^hi^CD11b^+^ myeloid cells **(C)**, and CD45^hi^CD11b^−^ putative lymphocyte **(D)** cell counts are shown. Representative histograms show the relative level of **E** CD68 protein expression and **F** LipiBlue-stained lipid bodies in microglia. **G** The percentage of Vglut1-positive microglia in each treatment group is quantified. **H** Representative dot plots illustrate the percentage of NeuN-positive microglia in the ipsilateral hemisphere at 8 weeks post-TBI. The mean fluorescence intensity of NeuN immunoreactivity is quantified. For all histograms, gray = FMO control, sucrose (vehicle) treated = blue, trehalose treated = red, sham controls = no fill, and TBI groups = bold fill. *N* = 9–10/group **(A–F)** and *N* = 4–6/group **(G–H)**. Data were analyzed using 2-way ANOVA group analysis with Tukey’s test for multiple comparisons. ****p < 0.0001, ** *p* < 0.01, * *p* < 0.05

To better understand the impact of trehalose on chronic microglia activation and neuroinflammation in old mice after TBI, we examined autophagic function at 9 weeks post-injury. Trehalose treatment caused a clear reduction in the number of proliferated microglia (CD45^int^CD11b^+^Ly6C^−^) present in the ipsilateral hemisphere and significantly prevented the delayed recruitment or accumulation of peripherally-derived myeloid (CD45^hi^CD11b^+^) and lymphocytes (CD45^hi^CD11b^−^) (Fig. 13A–D). Next, we examined the effect of trehalose on phagocytosis. A significant injury-related increase was found in microglial expression of CD68, but no effect of treatment was seen (Fig. 13E). Lipid content trended higher in sucrose-treated mice (*p* = 0.05), while trehalose treatment caused no change (Fig. 13F). The percentage of microglia that engulfed neuronal Vglut1 and the relative level of intracellular NeuN protein were both significantly lower in trehalose-treated mice (Fig. 13G–H).

**Fig. 14:**
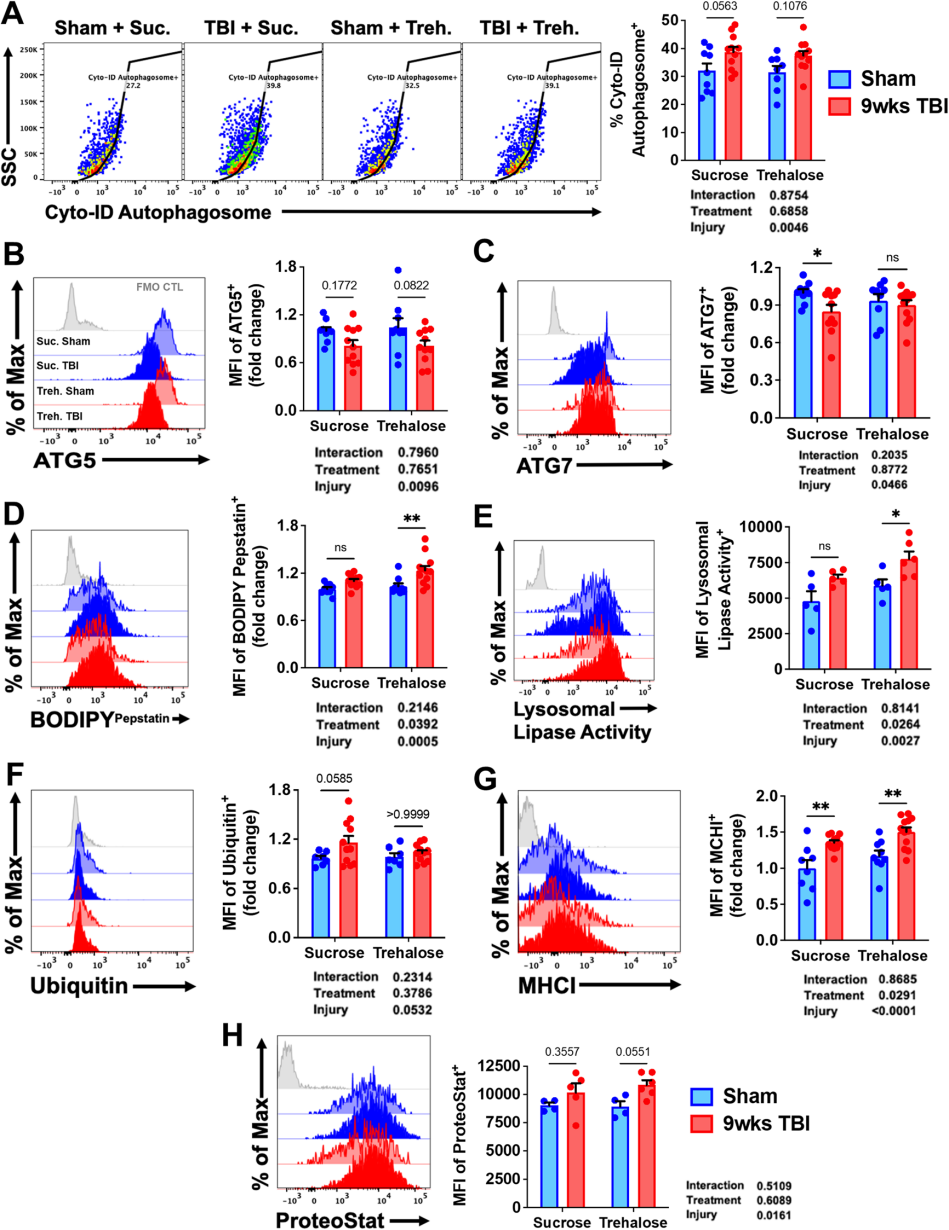
Trehalose treatment modulates multiple steps of the autophagy pathway in microglia. **A** A representative dot plot depicts the percentage of LC3II-positive autophagosomes in microglia at 9 weeks after TBI. Representative histograms show the relative protein expression level of the autophagy-related proteins **B** ATG5 and **C** ATG7 in microglia. Significant effects of trehalose treatment were seen in fluorescence intensity of **D** BODIPY^Pepstatin^ and **E** lysosomal lipase activity. Mean fluorescence intensities for **F** ubiquitin and **G** MHCI are quantified. **H** A main effect of injury was demonstrated in cytosolic protein aggregation as was measured using Proteostat dye. For all histograms, gray = FMO control, sucrose (vehicle) treated = blue, trehalose treated = red, sham controls = no fill, and TBI groups = bold fill. *N* = 9–10/group **(A**–**D** and **F**–**G)** and *N* = 4–6/group **(E** and **H)**. Data were analyzed using 2-way ANOVA group analysis with Tukey’s test for multiple comparisons. **p < 0.01, *p < 0.05

A comprehensive analysis of autophagy-related markers showed no effect of treatment or injury at 8 weeks as measured by LysoTracker dye, lamp1, lamp2, and Sqstm1/p62 (Supplementary Fig. S7A–D). Although increased formation of LC3II-positive autophagosomes were found in sucrose- but not trehalose-treated mice, this difference was not statistically significant (*p* = 0.05; Fig. 14A). A main effect of TBI was seen in ATG5 and ATG7 expression in microglia; trehalose treatment prevented a significant reduction in ATG7 (Fig. 14B–C). Examination of lysosomal enzymatic activity in microglia revealed a robust increase in cathepsin D and lysosomal lipase activity in mice treated with trehalose (Fig. 14D–E). There was a trend towards increased ubiquitin expression in microglia in sucrose-treated mice, but changes did not reach significance (*p* = 0.05; Fig. 14F). Whereas antigen presentation by MHCI was more augmented after trehalose treatment, it did not alter injury-induced increases in intracellular protein aggregation (Fig. 14G–H). Lastly, trehalose-mediated protection was associated with attenuated lysine acetylation in microglia (Supplementary Fig. S7E). Taken together, our results indicate that long-term trehalose treatment reduces chronic brain inflammation via modulation of several key proteins involved in the autophagy pathway, including the upstream regulation of phagocytosis.

## Discussion

We show that old age augments the expression of the disease-associated microglia signature during the chronic phase of TBI. This transcriptional and functional response is characterized by the downregulation of homeostatic pathways and upregulation of genes involved in lysosomal and phagocytic pathways. Neuronal/myelin engulfment, autophagic processes, and inflammatory activation were exacerbated with advanced age in microglia at both acute and chronic times after injury. Among other changes, the age- and injury-related increases in microglial lipofuscin accumulation and p62 at both the cell and tissue level implied that autophagic flux may be blocked during the acute and chronic stages of TBI. Continuous treatment with orally administered trehalose, an enhancer of autophagy, improved long-term neurological recovery in old mice, which was associated with chronically reduced phagocytic activity and increased lysosomal function. Our findings suggest that age-related dysregulation of autophagy plays a role in chronic microglial activation, neuroinflammation, and recovery following brain injury.

An extensive longitudinal assessment of functional recovery in aged mice following experimental TBI showed that older mice have similar recovery patterns for many, but not all behavioral tests compared to younger mice. Indeed, overall performance was more profoundly affected by normal aging than TBI. Thus, it can be problematic to differentiate effects of aging from those of injury due to differences in baseline performance. Thus, we have expressed the behavioral data as absolute changes rather than as percent changes from baseline to better illustrate recovery dynamics within groups. One conclusion from our findings is that older mice show some behavioral restoration late after TBI; thus, the same reparative processes that may be therapeutically modulated in young mice may also be targeted in older animals.

Using the NanoString Neuroinflammation Panel, TBI caused transcriptional activation of more than 100 additional inflammation-related genes in the cortex, and 40 fewer genes in the hippocampus of injured aged mice versus sham control as compared to changes in the injured young group. Based on the incremental increases in expression of DAM genes observed with both aging and chronic injury, these findings are consistent with the conclusion that TBI accelerates normal age-related expression of this inflammatory signature which may contribute to accelerated neurological impairment. Moreover, this work suggests that many prognostic biomarkers of TBI outcome, including the activation of specific gene networks, may need to be reassessed in older patients, given the normal age-related increases in gene expression levels and protein concentrations.

Age and injury-related increases in the expression of genes associated with the complement pathway is consistent with increased innate immune activation and supports a role for phagocytosis in the chronic brain injury setting. Complement activation products opsonize synaptic material on neurons for phagocytic removal, which during development, functions to prune excess synapses to refine neural circuitry [53]. This process is also important for synaptic plasticity throughout life. However, an increasing number of studies have recently demonstrated that excessive complement-mediated synaptic engulfment is a pathological process in the context of neurodegenerative disease, including TBI [22]. This would be consistent with our ex vivo phagocytosis data showing increased microglial engulfment of neuronal/synaptic antigens and decreased numbers of viable neurons and synaptic plasticity late after TBI [58]. Genes associated with astrocyte function were also relatively more upregulated in the aged brain late after TBI. Astrocytes can induce the expression of C1q in neurons via TGF-β [7]. C1q is involved in the pathological pathway of several neurodegenerative diseases due to its role in abnormal protein aggregate clearance, astrocyte reactivation, binding and activation of microglia, or inflammatory responses [4]. It remains to be seen whether complement gene expression is elevated in astrocytes during the chronic phase of TBI, and if C1q, C3, and C4a factors are localized at synapses and neurons in affected brain regions.

Brain aging leads to decline in autophagy efficiency, accumulation of lipofuscin, and increases in inflammatory activity including ROS production. Moreover, previous work has found that cellular and molecular markers of inflammation in the brain are acutely altered with age after TBI [6, 31, 45, 56]. The present study has now captured similar changes in aged mice during the chronic phase of TBI using a combination of ex vivo cellular assays, transcriptional gene profiling, and histological approaches. At the cellular level, the acute phase of injury was marked by an increase in infiltrating myeloid cells and microglial proliferation, whereas the chronic phase is highlighted by delayed lymphocyte recruitment. The leukocyte dynamics, however, were altered with age. Older mice showed significantly fewer microglia in general, either due cell senescence and/or death or the cell isolation procedure. Nonetheless, similar changes have been seen in other CNS cell types with age, including microglia [50]. Lymphocyte infiltration was exacerbated in old mice at all timepoints. Similar changes have been reported in aged models of ischemic stroke [8, 24], suggesting this may be a conserved age-related hallmark of brain injury. However, lymphocyte numbers were far exceeded by the resident microglial population. Although we made efforts to characterize the age-related temporal kinetics of microglial proliferation after TBI, two timepoints alone cannot accurately capture these dynamics, especially during the fast-evolving acute period. For example, in a previous study, we noted relatively greater microglial proliferation at 72-h post-injury in aged mice compared to young controls [56]. The fact that we did not observe this response at 48-h post-injury in our study might suggest that microglial proliferation is delayed with age. This would be in line with other reports, and our own data showing similar or higher expression of cell proliferation markers at that point in time for old microglia [56]. Although numbers of brain-infiltrating myeloid cells were similar between groups at 48 h, we had previously reported that the composition of these cells skewed more neutrophilic in older mice [56]. Thus, the cellular immune dynamics of the neuroinflammatory response to TBI are different with older animals. Changes in leukocyte number and composition, notwithstanding the effects of aging alone, suggest the quality and severity of immune function and inflammatory activity may be markedly different in older mice. However, the cell isolation procedure employed in this study, like most, have the potential to introduce biological artifacts [21, 42, 48]. We tried to limit these artifacts by eliminating the density gradient step, thereby shortening the duration of the procedure, and reducing toxicity due to high concentrations of, and time spent in, a sugar gradient. However, to measure coordinated protein effector responses that underlie complex cellular functions (i.e., phagocytosis, ROS generation, etc.) in the ex vivo setting requires incubation at in vivo–like temperatures (i.e., 37 °C) to promote activity. Furthermore, while we saw no evidence that microglia upregulated CD45 expression after injury using the Ly6C exclusion marker, we cannot exclude the possibility. Future studies using lineage tracing techniques are required to measure the stability of CD45 expression in microglia after CCI.

To ascertain whether microglial reactivity at acute and chronic stages of TBI was affected in old age, we performed an extensive profiling of cellular functions. In most measures, pro-inflammatory activity was relatively greater in older microglia after acute TBI. Of note, however, were the significant effects of age on phagocytosis, autophagy, and lipofuscin accumulation in microglia following TBI. In general, older microglia were more likely to engulf neurons and/or neuronal debris than their younger counterparts. Although the effects of TBI appear subtle at 16 weeks due to scaling and higher statistical stringency required for multiple comparison’s testing, we have previously reported a chronic increase in phagocytosis of apoptotic neurons and myelin debris as late as 8 months post-TBI [58]. The acute phase was also characterized by a robust age-related increase in lysosome and autophagosome formation. Lipofuscin accumulation was elevated in both age groups after injury. Interestingly, intracellular protein aggregation was reduced at 48 h in microglia; however, by 16 weeks post-injury, protein aggregation recrudesced in both age groups, implying late impairment of autophagic processes in microglia. These age-related alterations in phagocytosis, autophagy, and inflammatory activity are consistent with the age-related increase in the DAM gene signature, and its potential role in microglial activation changes during chronic brain injury.

We also identified novel age- and TBI-driven changes in epigenetic, metabolic, and senescence markers. The microglial activation, which often persists chronically following more severe head injury is consistent with an enduring change in cellular reprogramming mediated by epigenetic mechanisms, as we have suggested previously [25]. Earlier studies have reported imbalances in epigenetic marks of DNA methylation and histone acetylation after TBI [47]. Specifically, both acetylation and methylation of histones are decreased in the brain after CCI injury in rodents [2, 16, 60, 75]. TBI-induced histone acetylation is caused in part by increases in HDAC activity [20]. The functional implications of histone hypoacetylation are consistent with the neuroprotective effects shown with HDAC inhibition [59, 75]. Microglia-specific deletion of HDAC1 and HDAC2 has been shown to alter development, homeostasis, and activation [9]. Thus, it would appear histone deacetylation is a pathological feature of TBI and potential driver of microglial-mediated inflammation. The present study is among the first to examine these changes at the cellular level in microglia. We found that TBI not only reduced global lysine acetylation levels, but also histone acetylation. Moreover, these changes were lasting, exacerbated with age, and reversed with trehalose treatment. Thus, our data are consistent with the view that epigenetic changes of histones occur early after TBI and are sustained in microglia. Microglia adopted a senescence phenotype as a function of age and injury. The interaction between age and injury found in our study highlights a potential mechanism for the exaggerated inflammation and neurological dysfunction seen in older animals [1, 64, 65, 69].

In humans, men have more than a two-fold incidence of TBI than women [14] and sexually dimorphic neuropathological changes after TBI have been widely reported due to female sex hormones [44, 66]. To minimize variability of experimental outcomes and number of animals to reach statistical significance, all experiments in the present study were conducted using young adult male or aged male mice. However, sex differences in aging-mediated brain neuroinflammation following TBI have not been reported which is intriguing for future investigation.

Using aged animals, we employed a highly studied neuroprotectant and autophagic inducer as a potential therapeutic agent. Trehalose is currently in clinical trials for AD and neuronal ceroid-lipofuscinoses. Importantly, it is well-tolerated, widely available, low-cost, and can be orally administered without additives [29]. To assess longitudinal recovery in aged animals treated with trehalose, we performed a similar behavioral battery but with shorter injury duration to minimize long-term mortality. Sucrose was used as the control group because it shares a similar molecular structure with trehalose. Like many control solutions, sucrose is not an inert substance and may exert its own effects on metabolism and neurological recovery which have not been taken into consideration here. Nevertheless, trehalose was found to be more protective in aged mice after TBI. Previous work has shown that young mice treated with trehalose perform better in Morris water maze, OF testing, and Y-maze [51]. Furthermore, trehalose is known to also act as a chemical chaperone [63] and may therefore have protective actions that are independent of autophagy. For example, it has been reported that trehalose treatment is associated with upregulation of synaptophysin, doublecortin, and BDNF [51].

Given its ability to stabilize biomolecules and increase autophagy, trehalose has received growing attention as a potential neuroprotective agent [12, 29]. However, the precise mechanisms through which trehalose acts on the autophagy pathway in microglia have not been explored. Most importantly, we found that trehalose reduces overall numbers of inflammatory immune cells in the injured brain. Whether trehalose acts directly on the brain or in the periphery to achieve this effect is not clear. Because trehalose has been shown to cross the blood–brain barrier [41], concentrations are likely to be even further elevated under the compromised conditions associated with old age and TBI. The bidirectional relationship between the brain and the systemic environment suggests that the lack of precision targeting, whether possible or not, may be a moot point. Therefore, we believe the change in the functional state of microglia seen in old trehalose-treated mice underlie the associated behavioral improvements. Our results also showed unexpected changes in phagocytic activity late after TBI. Trehalose reduced continued phagocytosis of neurons and neuronal synapses, and attenuated lipid accumulation. This suggests that trehalose can act upstream of the autophagy pathway to potentially prevent the ingestion and accumulation of phagocytized material. Perhaps surprisingly, we did not see changes in lysosome formation or Sqstm1/p62 expression in microglia following trehalose treatment. Rather, the effects of trehalose on autophagy are likely more relevant during the acute phase of injury when phagocytosis and lysosomal degradation processes are sharply elevated [61]. Nonetheless, chronic increases in lysosomal enzyme activity were seen in trehalose-treated mice indicative of improved autophagy function. Further, trehalose prevented injury-induced increases in autophagosome formation and decreases in ATG7 expression late after TBI. Taken together, we identified several potential steps in the autophagy pathway where trehalose may exert important effects on microglia that are associated with better functional outcome following TBI.

Our histological, cellular, and molecular findings provided complementary evidence for age-related, chronic dysregulation of microglial function, and autophagic processes. We show that therapeutically modulating autophagy using trehalose can reduce chronic brain inflammation in aged mice and enhance long term recovery.

## Supplementary Information

Below is the link to the electronic supplementary material.

Supplementary file1 (PDF 2319 KB)

## Data Availability

All data generated or analyzed during this study are included in this article.

## Abbreviations

AD: Alzheimer’s disease
CW: Catwalk test
CCI: Controlled cortical impact
CNS: Central nervous system
DAM: Disease-associated microglia
DE: Differentially expressed
GS: Grip strength test
HP: Hot plate test
IHC: Immunohistochemistry
MS: Multiple sclerosis
NOR: Novel object recognition test
NSF: Novel suppressed feeding test
OF: Open field test
PCA: Principal component analysis
ROS: Reactive oxygen species
SR: Social recognition test
TBI: Traumatic brain injury
YM: Y-maze test
