# Supplementary material for: Functional and transcriptional profiling of microglial activation during the chronic phase of TBI identifies an age-related driver of poor outcome in old mice
Source: GeroScience. 2022 Apr 22;44(3):1407–40. doi: 10.1007/s11357-022-00562-y (PMC9213636; doi:10.1007/s11357-022-00562-y)
Supplement: Supplementary file 1 — Supplementary file1 (PDF 2319 KB) [file 11357_2022_562_MOESM1_ESM.pdf]

Functional and transcriptional profiling of microglial activation during the chronic phase of TBI identifies an age-related driver of poor outcome in old mice

Rodney M. Ritzel, Yun Li, Zhuofan Lei, Jordan Carter, Junyun He, Harry M.C. Choi, Niaz Khan, Hui Li, Samantha Allen, Marta M. Lipinski, Alan I. Faden, Junfang Wu*

Department of Anesthesiology and Shock, Trauma and Anesthesiology Research Center, University of Maryland School of Medicine, Baltimore, Maryland 21201, USA

Supplementary Material

Supplementary figures (S1-S7) and tables (S1-S3).

Supplementary Figure legends (S1-S7)

Supplementary Figure S1. Temporal gait dynamics in young and old mice after TBI. Catwalk gait analysis of right hind, left front, and left hind limbs is shown for the following parameters: **(A)** Stand (s), **(B)** Swing (s), **(C)** Swing Speed (cm/s), **(D)** Stride Length (cm), **(E)** Step Cycle (s), **(F)** Duty Cycle (%), **(G)** Body Speed (cm/s), and **(H)** Body Speed Variation (%). N=19 (Young TBI), and 17 (Old TBI). Data were analyzed using 2-way ANOVA group analysis with Tukey's test for multiple comparisons. ***p<0.001, **p< 0.01, *p<0.05.

Supplementary Figure S2. Differential expression analysis of transcription counts in cerebral cortex and hippocampus after TBI. (A-B) Pairwise group comparisons of differential gene expression were carried out with the nSolver Analysis program (p-value < 0.05). Four pairwise comparisons were performed for both cortical and hippocampus tissues: (1) Old Sham vs. Young Sham (*Set 1*); (2) Young TBI vs. Young Sham (*Set 2*); (3) Old TBI vs. Old Sham (*Set 3*); (4) Old TBI vs. Young TBI (*Set 4*). **(C-D)** Venn diagram of differential expressed genes demonstrates the separation of injury- and age-related genes based on their membership in *Set 1* and *4* or *Set 2* and *3*. N=6 mice/group.

Supplementary Figure S3. qPCR validation of Nanostring data for the cortex at 16w after TBI. Gene expression changes in the cerebral cortex were validated for NanoString target genes related to the **(A)** complement pathway, **(B)** immune pathways, **(C)** autophagy and endocytosis pathways, **(D)** epigenetic regulation, and **(E)** neuronal activity. N=6-7/group. Data were analyzed

using 2-way ANOVA group analysis with Tukey’s test for multiple comparisons. ****$p<0.0001$, ***$p<0.001$, **$p<0.01$, *$p<0.05$.

Supplementary Figure S4. qPCR validation of Nanostring data for the hippocampus at 16w after TBI. Gene expression changes in the hippocampus were validated for NanoString target genes related to the **(A)** complement pathway, **(B)** immune pathways, **(C)** autophagy and endocytosis pathways, **(D)** epigenetic regulation, **(E)** endothelial regulation, and **(F)** neuronal activity. N=6-7/group. Data were analyzed using two-way ANOVA group analysis with Tukey’s test for multiple comparisons. ****$p<0.0001$, ***$p<0.001$, **$p<0.01$, *$p<0.05$.

Supplementary Figure S5. Temporal alterations in microglial lipofuscin content after TBI. Representative histograms depict the relative **(A)** forward and **(B)** side light scatter intensities of microglia at 48h and 16w post-TBI. The mean fluorescence intensity of **(C)** BODIPY$^{493/503}$- and **(D)** FerroOrange-stained microglia is quantified. Representative histograms illustrate the relative level of **(E)** cytosolic protein aggregation and **(F)** autofluorescence in the empty PE channel. For all histograms, gray = FMO control, blue = young, red = old, Sham = no outline/no fill, 48h TBI = bold outline/no fill, and 12w TBI = bold outline/bold fill. N=5-7/group. Data were analyzed using two-way ANOVA group analysis with Tukey’s test for multiple comparisons. ***$p<0.001$, **$p<0.01$, *$p<0.05$.

Supplementary Figure S6. Effects of trehalose treatment on cognitive and social behavior. **(A)** Y-maze experiment showed a significant decrease of arm entry number at 1w post-injury in both age groups. (**B**) NOR task showed no group differences in exploration time of left versus right objects during the sample phase. (**C-D**) Graphs depicting the preference index of each sequence for the social recognition experiment. In sequence 3, The TBI sucrose group failed to show preference for the second, novel stranger mouse, which suggests neurological deficits in social interaction. ****$p<0.0001$, ***$p<0.001$, **$p<0.01$, *$p<0.05$.

Supplementary Figure S7. Effects of trehalose treatment on microglial function. Representative histograms depict the relative abundance of **(A)** lysosomal content, and protein expression of **(B)** Lamp1, **(C)** Lamp2, and **(D)** Sqstm1/p62. No differences were seen between groups. **(E)** A significant effect of Trehalose treatment was seen in the frequency of lysine acetylated microglia. For all histograms, gray = FMO control, Sucrose (vehicle) treated = Blue, Trehalose treated = Red, Sham controls = no fill, and TBI groups = bold fill. N=9-10/group **(A-C)** and N=4-6/group **(D)**. Data were analyzed using 2-way ANOVA group analysis with Tukey’s test for multiple comparisons. ***$p<0.001$.

Fig S1

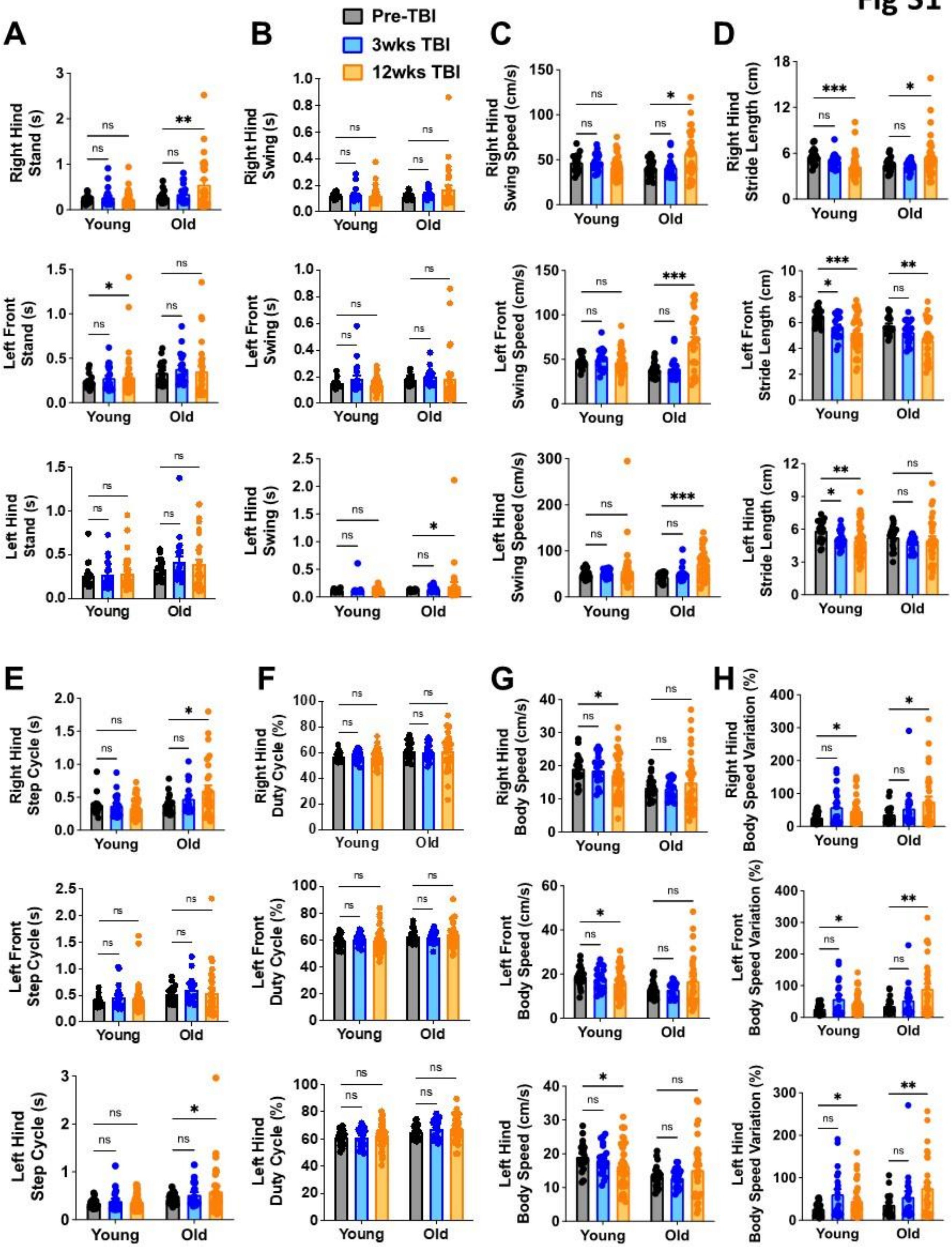

Pre-TBI
3wks TBI
12wks TBI
A
B
C
D
Right Hind Stand (s)
Right Hind Swing (s)
Right Hind Swing Speed (cm/s)
Right Hind Stride Length (cm)
Left Front Stand (s)
Left Front Swing (s)
Left Front Swing Speed (cm/s)
Left Front Stride Length (cm)
Left Hind Stand (s)
Left Hind Swing (s)
Left Hind Swing Speed (cm/s)
Left Hind Stride Length (cm)
E
F
G
H
Right Hind Step Cycle (s)
Right Hind Duty Cycle (%)
Right Hind Body Speed (cm/s)
Right Hind Body Speed Variation (%)
Left Front Step Cycle (s)
Left Front Duty Cycle (%)
Left Front Body Speed (cm/s)
Left Front Body Speed Variation (%)
Left Hind Step Cycle (s)
Left Hind Duty Cycle (%)
Left Hind Body Speed (cm/s)
Left Hind Body Speed Variation (%)
Young
Old
ns

Fig S2

A
Cortex

Differential Expression (DE) Analysis

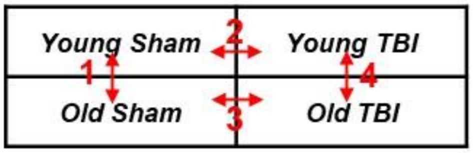

Pairwise Comparison	DE Genes ↓ Expression	DE Genes ↑ Expression
Set 1	59	71
Set 2	101	23
Set 3	225	28
Set 4	149	29

B
Hippocampus

Differential Expression (DE) Analysis

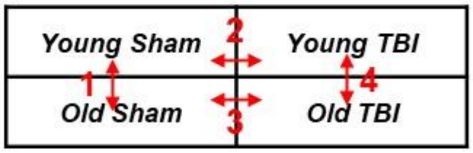

Pairwise Comparison	DE Genes ↓ Expression	DE Genes ↑ Expression
Set 1	74	47
Set 2	126	21
Set 3	83	27
Set 4	64	77

C
Cortex

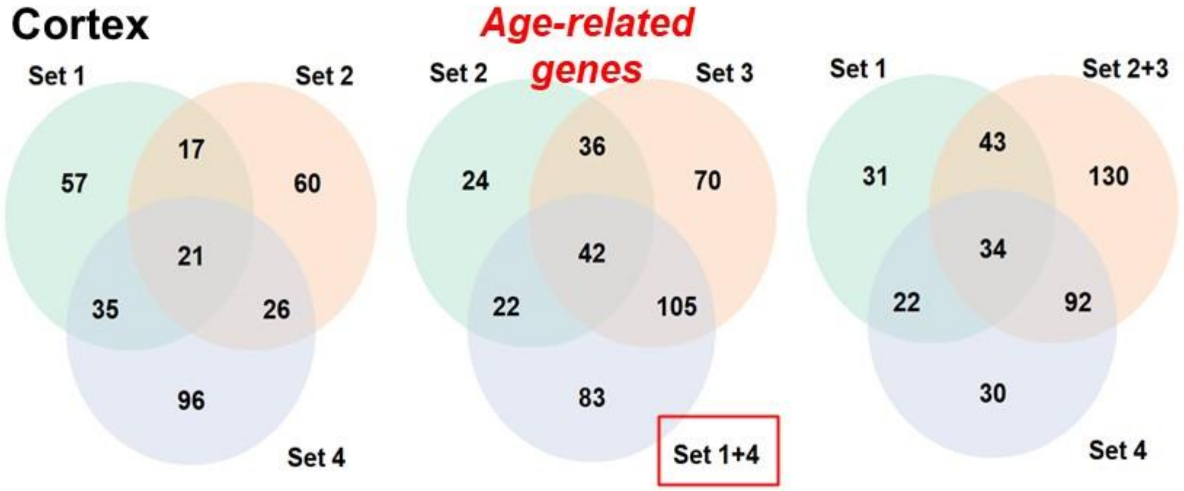

D
Hippocampus

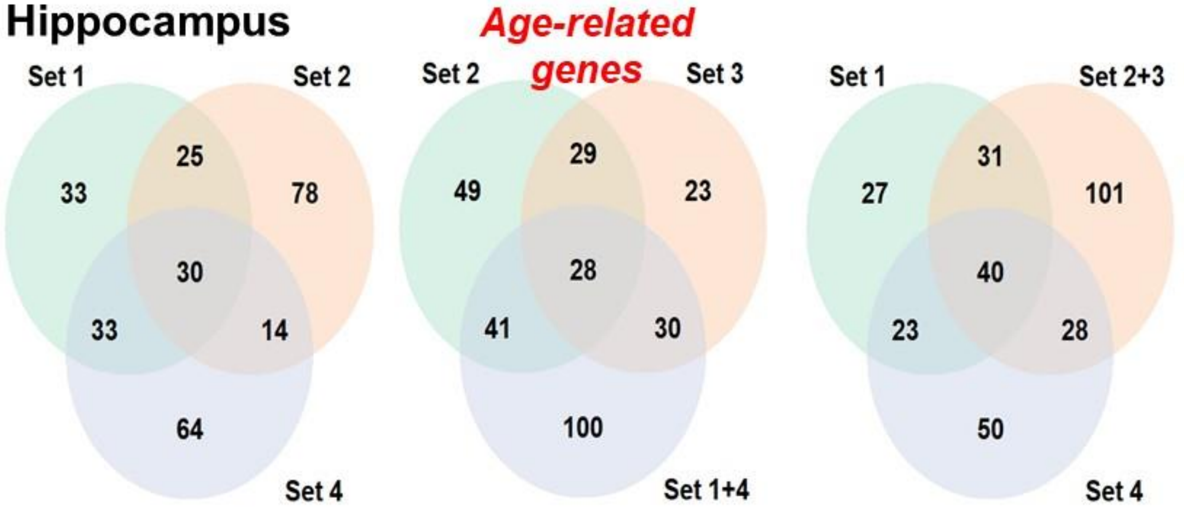

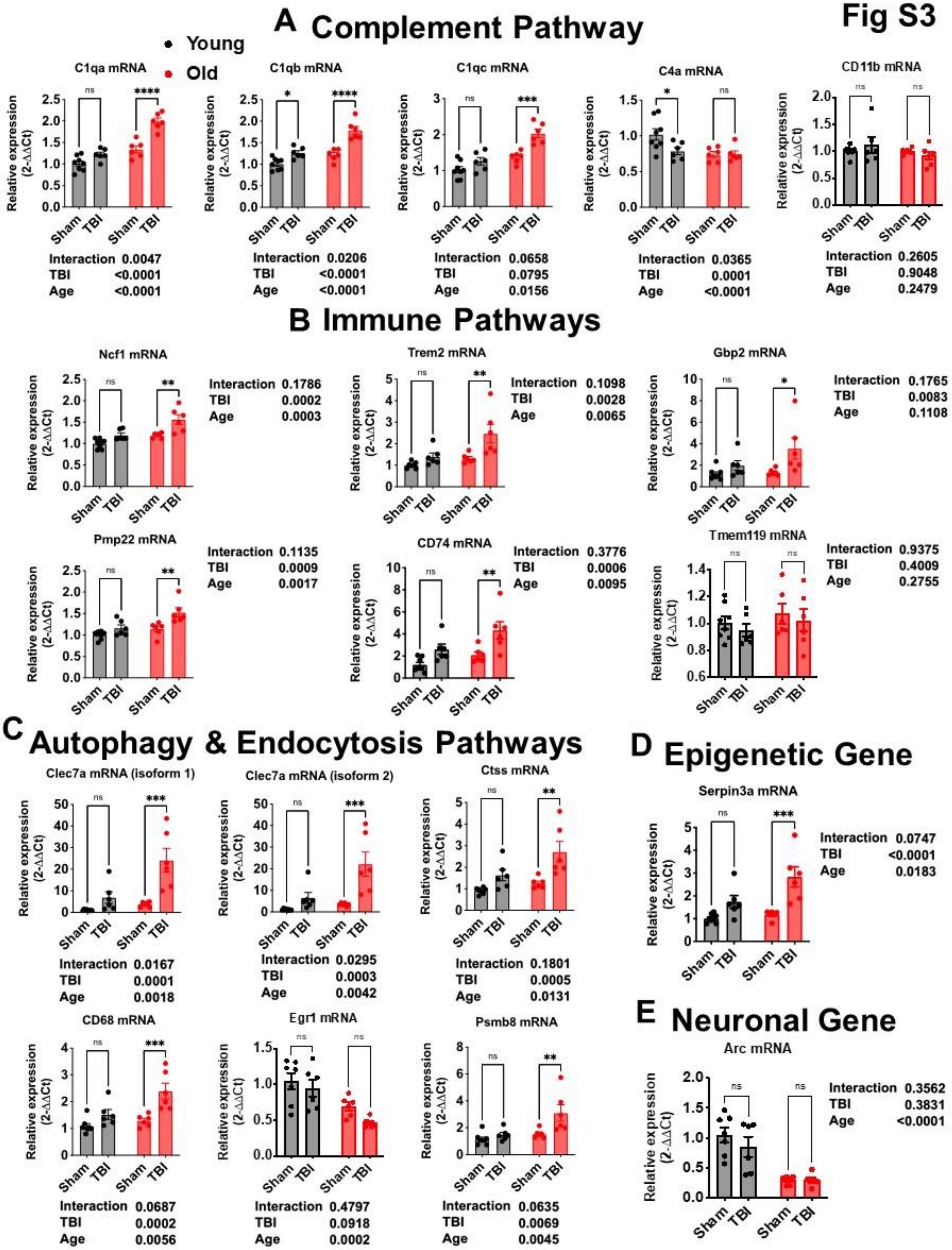
Fig S3
A Complement Pathway
Young
Old
C1qa mRNA
Interaction 0.0047
TBI <0.0001
Age <0.0001
C1qb mRNA
Interaction 0.0206
TBI <0.0001
Age <0.0001
C1qc mRNA
Interaction 0.0658
TBI 0.0795
Age 0.0156
C4a mRNA
Interaction 0.0365
TBI 0.0001
Age <0.0001
CD11b mRNA
Interaction 0.2605
TBI 0.9048
Age 0.2479
B Immune Pathways
Ncf1 mRNA
Interaction 0.1786
TBI 0.0002
Age 0.0003
Trem2 mRNA
Interaction 0.1098
TBI 0.0028
Age 0.0065
Gbp2 mRNA
Interaction 0.1765
TBI 0.0083
Age 0.1108
Pmp22 mRNA
Interaction 0.1135
TBI 0.0009
Age 0.0017
CD74 mRNA
Interaction 0.3776
TBI 0.0006
Age 0.0095
Tmem119 mRNA
Interaction 0.9375
TBI 0.4009
Age 0.2755
C Autophagy & Endocytosis Pathways
Clec7a mRNA (isoform 1)
Interaction 0.0167
TBI 0.0001
Age 0.0018
Clec7a mRNA (isoform 2)
Interaction 0.0295
TBI 0.0003
Age 0.0042
Ctss mRNA
Interaction 0.1801
TBI 0.0005
Age 0.0131
CD68 mRNA
Interaction 0.0687
TBI 0.0002
Age 0.0056
Egr1 mRNA
Interaction 0.4797
TBI 0.0918
Age 0.0002
Psmb8 mRNA
Interaction 0.0635
TBI 0.0069
Age 0.0045
D Epigenetic Gene
Serpin3a mRNA
Interaction 0.0747
TBI <0.0001
Age 0.0183
E Neuronal Gene
Arc mRNA
Interaction 0.3562
TBI 0.3831
Age <0.0001
Relative expression (2-ΔΔCt)
Sham TBI Sham TBI

Fig S4

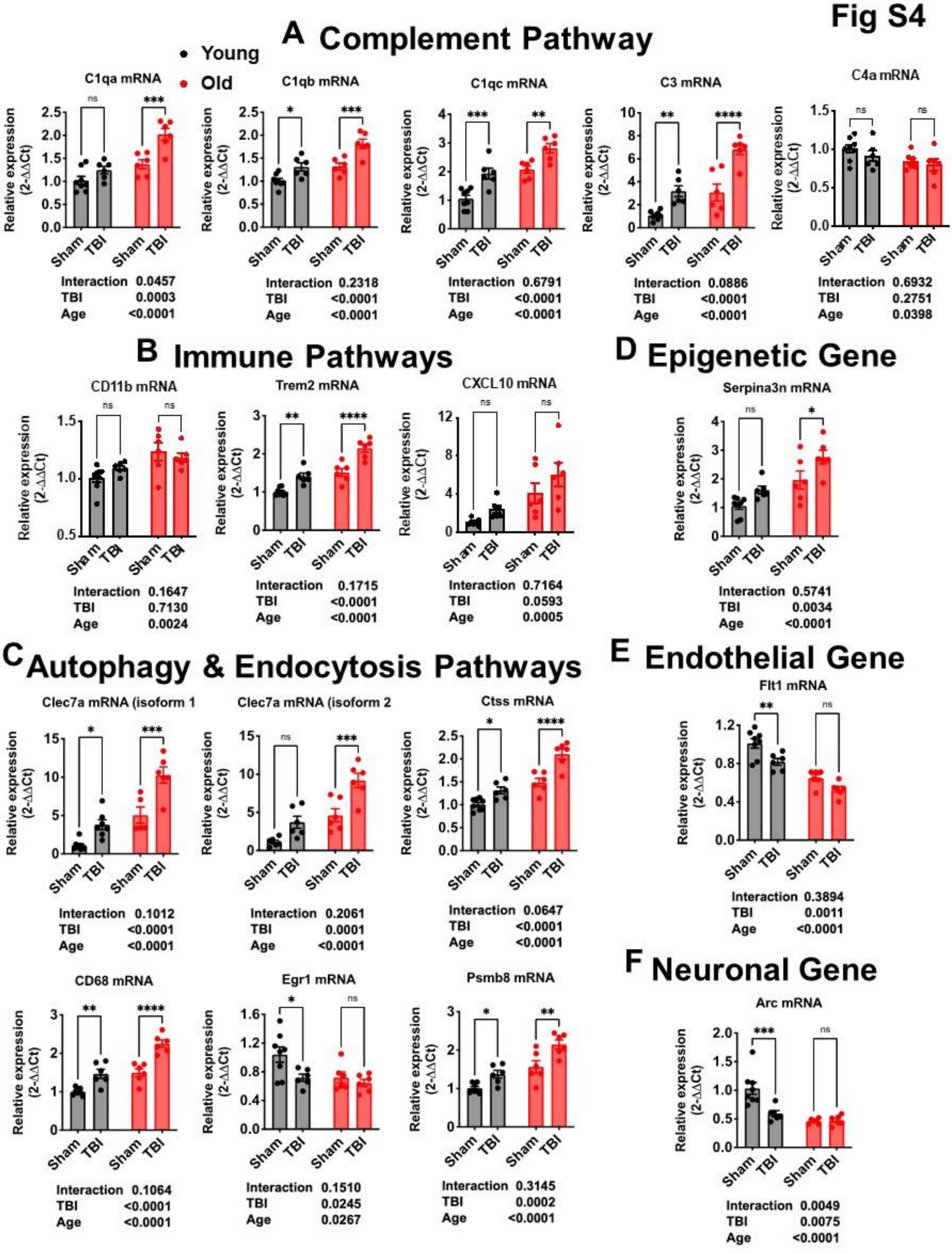
A Complement Pathway
Young
Old
C1qa mRNA
C1qb mRNA
C1qc mRNA
C3 mRNA
C4a mRNA
Relative expression (2-ΔΔCt)
Sham TBI Sham TBI
ns
*
***
**
****
C1qa mRNA: Interaction 0.0457, TBI 0.0003, Age <0.0001
C1qb mRNA: Interaction 0.2318, TBI <0.0001, Age <0.0001
C1qc mRNA: Interaction 0.6791, TBI <0.0001, Age <0.0001
C3 mRNA: Interaction 0.0886, TBI <0.0001, Age <0.0001
C4a mRNA: Interaction 0.6932, TBI 0.2751, Age 0.0398
B Immune Pathways
CD11b mRNA
Trem2 mRNA
CXCL10 mRNA
CD11b mRNA: Interaction 0.1647, TBI 0.7130, Age 0.0024
Trem2 mRNA: Interaction 0.1715, TBI <0.0001, Age <0.0001
CXCL10 mRNA: Interaction 0.7164, TBI 0.0593, Age 0.0005
D Epigenetic Gene
Serpina3n mRNA
Serpina3n mRNA: Interaction 0.5741, TBI 0.0034, Age <0.0001
C Autophagy & Endocytosis Pathways
Clec7a mRNA (isoform 1
Clec7a mRNA (isoform 2
Ctss mRNA
Clec7a mRNA (isoform 1: Interaction 0.1012, TBI <0.0001, Age <0.0001
Clec7a mRNA (isoform 2: Interaction 0.2061, TBI 0.0001, Age <0.0001
Ctss mRNA: Interaction 0.0647, TBI <0.0001, Age <0.0001
CD68 mRNA
Egr1 mRNA
Psmb8 mRNA
CD68 mRNA: Interaction 0.1064, TBI <0.0001, Age <0.0001
Egr1 mRNA: Interaction 0.1510, TBI 0.0245, Age 0.0267
Psmb8 mRNA: Interaction 0.3145, TBI 0.0002, Age <0.0001
E Endothelial Gene
Flt1 mRNA
Flt1 mRNA: Interaction 0.3894, TBI 0.0011, Age <0.0001
F Neuronal Gene
Arc mRNA
Arc mRNA: Interaction 0.0049, TBI 0.0075, Age <0.0001

Fig S5

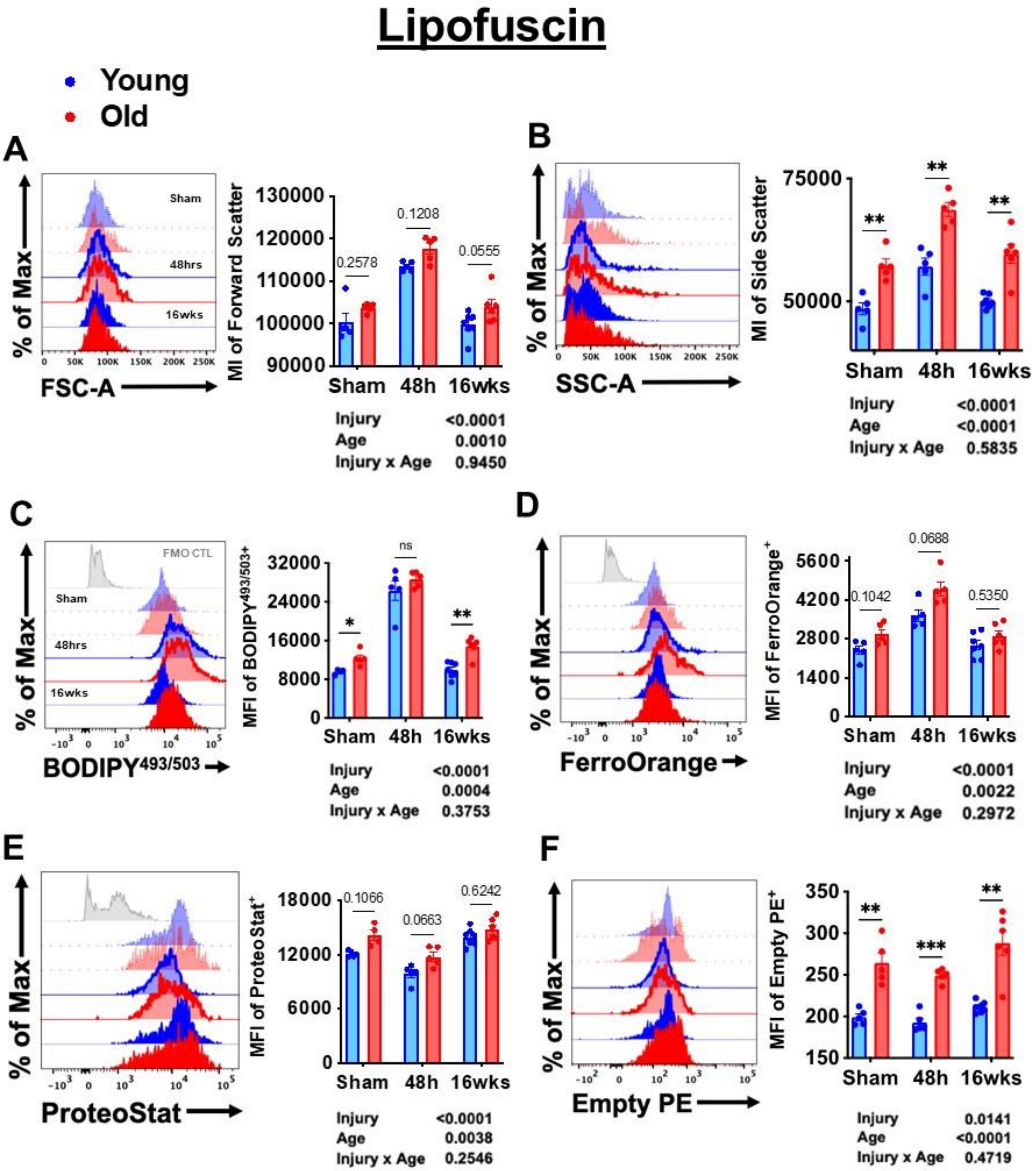
Lipofuscin
Young
Old
A
% of Max
FSC-A
Sham
48hrs
16wks
MI of Forward Scatter
0.2578
0.1208
0.0555
Injury <0.0001
Age 0.0010
Injury x Age 0.9450
B
SSC-A
MI of Side Scatter
Injury <0.0001
Age <0.0001
Injury x Age 0.5835
C
FMO CTL
BODIPY493/503
MFI of BODIPY493/503+
ns
Injury <0.0001
Age 0.0004
Injury x Age 0.3753
D
FerroOrange
MFI of FerroOrange+
0.1042
0.0688
0.5350
Injury <0.0001
Age 0.0022
Injury x Age 0.2972
E
ProteoStat
MFI of ProteoStat+
0.1066
0.0663
0.6242
Injury <0.0001
Age 0.0038
Injury x Age 0.2546
F
Empty PE
MFI of Empty PE+
Injury 0.0141
Age <0.0001
Injury x Age 0.4719

Fig S6

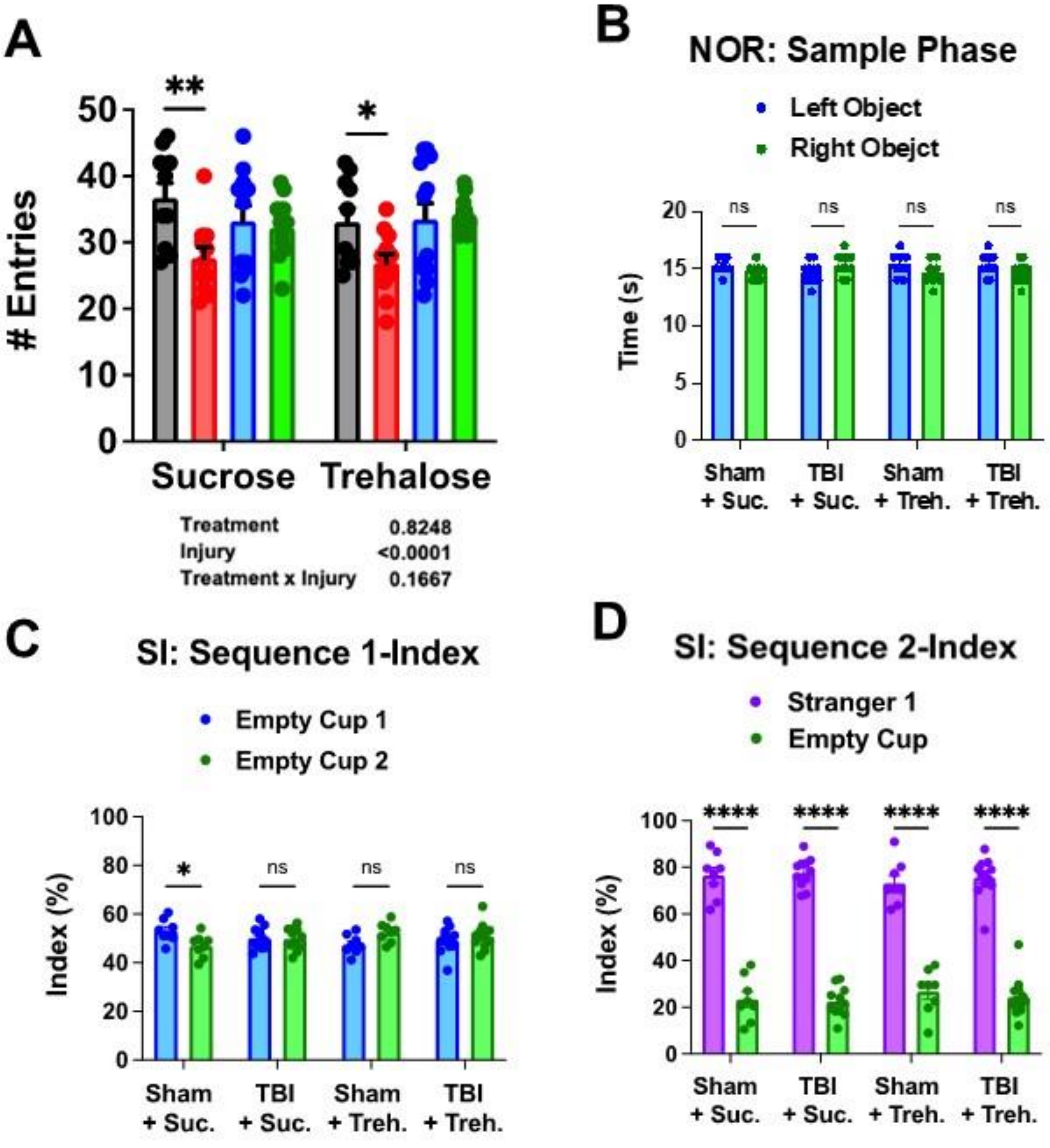
A
**
*
50
40
30
20
10
0
Entries
Sucrose
Trehalose
Treatment 0.8248
Injury <0.0001
Treatment x Injury 0.1667
B
NOR: Sample Phase
Left Object
Right Obejct
20
15
10
5
0
Time (s)
ns
Sham + Suc.
TBI + Suc.
Sham + Treh.
TBI + Treh.
C
SI: Sequence 1-Index
Empty Cup 1
Empty Cup 2
100
80
60
40
20
0
Index (%)
*
ns
Sham + Suc.
TBI + Suc.
Sham + Treh.
TBI + Treh.
D
SI: Sequence 2-Index
Stranger 1
Empty Cup
100
80
60
40
20
0
Index (%)
****
Sham + Suc.
TBI + Suc.
Sham + Treh.
TBI + Treh.

Fig S7

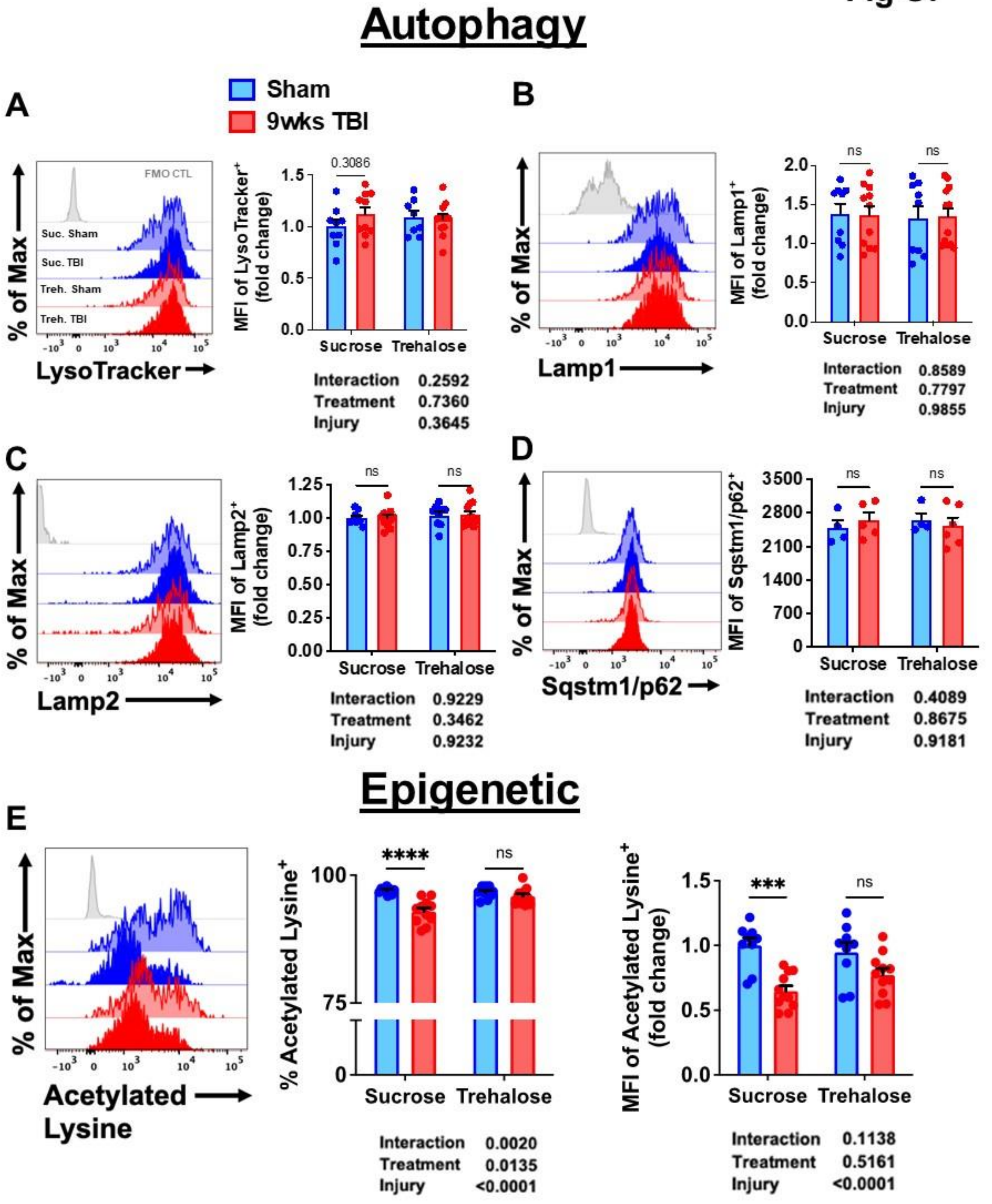
Autophagy
A
Sham
9wks TBI
% of Max
FMO CTL
Suc. Sham
Suc. TBI
Treh. Sham
Treh. TBI
LysoTracker
MFI of LysoTracker+ (fold change)
0.3086
Sucrose
Trehalose
Interaction 0.2592
Treatment 0.7360
Injury 0.3645
B
% of Max
Lamp1
MFI of Lamp1+ (fold change)
ns
Sucrose
Trehalose
Interaction 0.8589
Treatment 0.7797
Injury 0.9855
C
% of Max
Lamp2
MFI of Lamp2+ (fold change)
ns
Sucrose
Trehalose
Interaction 0.9229
Treatment 0.3462
Injury 0.9232
D
% of Max
Sqstm1/p62
MFI of Sqstm1/p62+
ns
Sucrose
Trehalose
Interaction 0.4089
Treatment 0.8675
Injury 0.9181
Epigenetic
E
% of Max
Acetylated Lysine
% Acetylated Lysine+
****
ns
Sucrose
Trehalose
Interaction 0.0020
Treatment 0.0135
Injury <0.0001
MFI of Acetylated Lysine+ (fold change)
***
ns
Sucrose
Trehalose
Interaction 0.1138
Treatment 0.5161
Injury <0.0001

Supplementary Tables (S1-S3)

Table S1. qPCR primers and immuno-labeling reagents.

Table S2. The average transcription counts for each group after normalization with housekeeping genes in the ipsilateral cortex.

Table S3. The average transcription counts for each group after normalization with housekeeping genes in the ipsilateral hippocampus.

Table S1. qPCR primers and immuno-labeling reagents.

Antigen	Host, dilution	Vendor	Catalog number
qPCR primers			
Clec7a (isoform 1)	Mouse	Applied Biosystems	Mm01183349_m1
Clec7a (isoform 2)	Mouse	Applied Biosystems	Mm01183350_m1
Ncf1	Mouse	Applied Biosystems	Mm00447921_m1
Ctss	Mouse	Applied Biosystems	Mm01255859_m1
C1qa	Mouse	Applied Biosystems	Mm00432142_m1
C1qb	Mouse	Applied Biosystems	Mm00437836_m1
C1qc	Mouse	Applied Biosystems	Mm00776126_m1
C3	Mouse	Applied Biosystems	Mm00437838_m1
Itgam (Cd11b)	Mouse	Applied Biosystems	Mm00434455_m1
Cxcl10	Mouse	Applied Biosystems	Mm00445235_m1
Pmp22	Mouse	Applied Biosystems	Mm01333393_m1
Serpin3a	Mouse	Applied Biosystems	Mm00776430_m1
Arc	Mouse	Applied Biosystems	Mm01204954_g1
Egr1	Mouse	Applied Biosystems	Mm00656724_m1
C4a	Mouse	Applied Biosystems	Mm01132415_g1
Gbp2	Mouse	Applied Biosystems	Mm00494576_g1
Trem2	Mouse	Applied Biosystems	Mm04209424_g1
Cd74	Mouse	Applied Biosystems	Mm00658576_m1
Cd68	Mouse	Applied Biosystems	Mm03047343_m1
Psmb8	Mouse	Applied Biosystems	Mm00440207_m1
Tmem119	Mouse	Applied Biosystems	Mm00525305_m1
Egfr	Mouse	Applied Biosystems	Mm01187858_m1
Flt1	Mouse	Applied Biosystems	Mm00438980_m1
Gapdh	Mouse	Applied Biosystems	Mm99999915_g1
Flow cytometry antibodies			
Surface antigens			
CD45-eF450	Mouse, 1:100	eBioscience	48-0451-82
CD11b-APCeF780	Mouse, 1:100	eBioscience	47-0112-82
Ly6C-AF700	Mouse, 1:50	Biolegend	128024

MHCI-PECy7	Mouse, 1:100	Biolegend	114616
Intracellular staining			
	Mouse, 1:100	Biolegend	652426
Ki67-PECy7	Mouse, 1:50	Biolegend	307912
PCNA-AF647	Mouse, 1:100	Biolegend	137010
CD68-PerCPCy5.5	Mouse, 1:100	Millipore Sigma	FCMAB317PE
NeuN-PE	Mouse, 1:100	StressMarq	SMC-394D-APC
Vglut1-APC	Mouse, 1:100	Biolegend	121626
Lamp1-PerCPCy5.5	Mouse, 1:100	Biolegend	108506
Lamp2-PE	Mouse, 1:20	Novus Biologicals	NBP1-42822AF647
Sqstm1/p62-AF647	Mouse, 1:50	Biolegend	847410
ATG5-AF647	Mouse, 1:50	R&D Systems	FAB6608N
ATG7-AF700	Mouse, 1:100	Biolegend	838710
Ubiquitin-AF647	Mouse, 1:100	Cell Signaling	12230S
H3-AF647	Mouse, 1:20	Technology	623408
Acetylated (Ac)		Biolegend	
Lysine (Lys)-PECy7	Mouse, 1:100		9683S
H3-Ac-Lys9-AF488	Mouse, 1:100	Cell Signaling	73508S
H3-Ac-Lys18-AF488	Mouse, 1:100	Technology	39030S
H3-Ac-Lys27-AF647	Mouse, 1:100	Cell Signaling	84061S
H3-Ac-Lys36-AF647	Mouse, 1:20	Technology	613420
Phospho(ser149)-		Cell Signaling	
H2A.X-PECy7	Mouse, 1:100	Technology	SPC-1280D-APC
p16-APC	Mouse, 1:100	Cell Signaling	NBP2-43697AF488
p21-AF488	Mouse, 1:50	Technology	506324
TNF-PECy7	Mouse, 1:50	Biolegend	46-7114-82
IL-1β-PerCPeF710	Mouse,		420601
Brefeldin A	1:1000	StressMarq	
		RND	
Ex vivo cell staining		Biolegend	
		eBioscience	
		Biolegend	
H2dcfda (DCF)	8 μM	ThermoFisher	D399
LysoTracker Deep	75 nM	ThermoFisher	L12492
Red	1:1000	Enzo Life Sciences	ENZ-51031-K200
Cyto-ID Autophagy			
Detection Kit	1:1000	Enzo Life Sciences	ENZ-51035-K100
ProteoStat			
Aggresome	2 μM	ThermoFisher	D3922
Detection Kit	0.5 μmol/L	Dojindo	LD01-10
BODIPY 493/503	1:300	ThermoFisher	F34652
Lipi-Blue	1 μmol/L	Dojindo	F374-12
FluoroMyelin Red	25 nM	Biolegend	424802

FerroOrange	200 μg/mL	Cayman Chemical	600470
MitoSpy Red		Company	
CMXRos	10 μM	Millipore Sigma	SCT045
Glucose Uptake	1 μM	ThermoFisher	P12271
Assay Kit	1:500	Abcam	ab253380
Biotracker ATP-Red			
BODIPY-Pepstatin A			
LysoLive™			
Lysosomal Acid			
Lipase Assay Kit			
IHC antibodies			
NeuN	Mouse, 1:500	Millipore	MAB377
LC3	Rabbit, 1:200	Novus Biologicals	NB100-2220
P62	Guinea Pig,	Progen	GP62-C
Iba-1	1:500	Cell Signaling	17198S
	Rabbit, 1:200	Technology	

Table S2. The average transcription counts for each group after normalization with housekeeping genes in the ipsilateral cortex.

mRNA	Accession #	Y/Sham	Y/TBI	O/Sham	O/TBI
Abcc3	NM_029600.3	29.21	34.55	35.31	33.92
Abcc8	NM_011510.3	67.74	59.94	82.42	59.93
Abl1	NM_009594.4	189.06	181.85	184.26	214.9
Adamts16	NM_172053.2	16.59	17.21	15.92	14.27
Ago4	NM_153177.3	45.72	47.56	34.33	40.47
Agt	NM_007428.3	48.92	56.5	52.55	86.5
AI464131	NM_001085515.2	196.81	206	204.3	234.09
Ak1	NM_001198790.1	1570.47	1476.82	1661.24	1564.72
Akt1	NM_001165894.1	849.55	832.86	931.71	933.48
Akt2	NM_001110208.1	351.91	311.64	333.82	338.96
Aldh1l1	NM_027406.1	555.85	547.97	607.51	571.96
Ambra1	NM_001080754.1	873.24	863.36	845.12	843.62
Amigo2	NM_178114.4	92.86	99.97	98.26	113.1
Anapc15	NM_027532.3	235.36	234.19	247.51	250.55
Anxa1	NM_010730.2	19.91	28.87	23.07	29.66
Apc	NM_007462.3	4574	4481.47	4340.03	4244.29
Apex1	NM_009687.2	233.13	274.25	261.42	273.72
Apoe	NM_001305844.1	4332.77	5219.54	4588.61	5845.57
Arc	NM_018790.2	1393.68	874.79	418.49	422.63

Arhgap24	NM_029270.2	83.93	79.64	65.04	94.47
Arid1a	NM_001080819.1	570.62	558.6	530.99	568.58
Asb2	NM_023049.1	21.29	19.61	20.06	24
Ash2l	NM_001080793.1	328.5	356.3	298.25	320.22
Asph	NM_001177849.1	2822.34	2766.23	2877.81	2873.61
Atf3	NM_007498.3	14.75	20.92	13.54	17.06
Atg14	NM_172599.4	277.61	278.53	255.74	252.56
Atg3	NM_026402.3	1423.62	1444.55	1442.03	1404.09
Atg5	NM_001314013.1	624.99	625.86	608.94	596.95
Atg7	NM_028835.1	234.84	236.94	258.46	231.88
Atg9a	NM_001003917.3	1557.32	1435.42	1542.29	1469.43
Atm	NM_007499.2	210.18	194.26	196.62	201.06
Atp6v0e	NM_025272.2	177.19	202.55	172.66	201.41
Atp6v1a	NM_007508.5	5225.19	5331.43	5760.03	5280.07
Atr	NM_019864.1	134.73	130.88	138.62	127.91
Axl	NM_009465.3	253.48	258.42	283.96	341.75
B3gnt5	NM_001159407.1	16.15	20.45	17.32	15.94
Bad	NM_007522.3	357.14	374.76	379.09	387.84
Bag3	NM_013863.4	60.55	63.27	65.43	69.74
Bag4	NM_026121.3	1056.85	1113.56	1162.08	1122.59
Bak1	NM_007523.2	201.04	191.78	185.87	208.56
Bard1	NM_007525.3	21.91	19.02	17.03	22.13
Bax	NM_007527.3	196.89	191.09	178.19	183.77
Bbc3	NM_133234.1	54.64	48.2	44.59	53.26
Bcas1	NM_029815.2	1083.68	1131.67	988.97	1368.15
Bcl10	NM_009740.1	290.57	283.22	266.91	283.72
Bcl2	NM_009741.3	109.79	105.79	108.38	103.23
Bcl2a1a	NM_009742.3	44.52	63.11	54.56	87.09
Bcl2l1	NM_009743.4	688.46	701.67	679.03	720.85
Bcl2l11	NM_001284410.1	34.83	31.48	30.07	35.19
Bcl2l2	NM_007537.1	1565.22	1477.57	1496.62	1447.83
Bdnf	NM_007540.4	344.88	338.06	268.14	239.64
Becn1	NM_019584.3	1874.02	1815.61	1790.95	1817.78
Bid	NM_007544.3	146.66	148.97	174.31	171.85
Bik	NM_007546.2	13.81	11.72	12.6	10.3
Bin1	NM_001083334.1	1444.89	1466.64	1511.76	1571.24
Birc2	NM_007465.2	472.71	499.52	510.12	489.81
Birc3	NM_007464.3	20.36	24.1	21.21	19.11
Birc5	NM_009689.2	21.44	27.42	22.3	22.23
Blk	NM_007549.2	14.56	17.14	13.7	14.6
Blm	NM_001042527.2	49.11	48.44	56.45	50.48

Blnk	NM_008528.4	112.28	121.42	118.48	116.45
Bmi1	NM_007552.4	492.33	492.6	461.23	486.02
Bnip3	NM_009760.4	2835.54	2928.67	3056.62	2851.47
Bnip3l	NM_009761.3	1368.85	1395.44	1420.86	1391.57
Bok	NM_016778.2	452.62	400.47	443.77	450.37
Bola2	NM_175103.3	1245.08	1276.11	1273.2	1395.39
Braf	NM_139294.5	1171.09	1160.13	1151.7	1144.59
Brca1	NM_009764.3	16.54	14.73	11.55	16.65
Brd2	NM_010238.3	1398.52	1323.21	1340.29	1363.86
Brd3	NM_001113573.1	410.19	407.31	388.17	404.85
Brd4	NM_001286630.1	699.81	675.39	648.3	677.31
Btk	NM_013482.2	22.05	25.6	21.47	31.98
C1qa	NM_007572.2	474.4	642.15	618.72	966.02
C1qb	NM_009777.2	800.7	1039.87	970.21	1429.34
C1qc	NM_007574.2	758.73	993.66	971.68	1410.91
C3	XM_011246258.1	14.16	30.47	13.86	53.05
C3ar1	NM_009779.2	52.07	58.95	63.6	88.71
C4a	NM_011413.2	80.28	287.5	227.82	623.91
C5ar1	NM_007577.3	21.77	28.44	25.81	29.04
C6	NM_016704.2	13.54	11.72	10.11	10.28
Cables1	NM_001146287.1	430.34	451.24	467.34	445.87
Calcoco2	NM_001271018.1	13.54	14.05	10.11	10.42
Calr	NM_007591.3	6616.55	6330.69	5941.43	5984.64
Camk4	NM_009793.3	2279.65	2042.54	2063.21	2115.39
Casp1	NM_009807.2	67.08	80.48	124.59	92.51
Casp2	NM_007610.1	156.75	144.96	135.53	135.93
Casp3	NM_009810.2	149.69	153.3	145.67	156.65
Casp4	NM_007609.2	16.09	19.59	18.91	22.62
Casp6	NM_009811.3	47.4	51.08	45.94	47.87
Casp7	NM_007611.2	52.32	45.66	48.83	57.51
Casp8	NM_009812.2	33.59	37.61	37.01	50.83
Casp9	NM_015733.4	171.15	158.44	172.97	170.49
Cass4	NM_001080820.2	13.54	14.3	12.36	12
Ccl2	NM_011333.3	14.02	18.46	12.44	18.53
Ccl3	NM_011337.1	18.17	21.8	23.75	33.52
Ccl4	NM_013652.1	14.05	14.41	11.51	12.22
Ccl5	NM_013653.1	13.54	13.78	10.27	16.44
Ccl7	NM_013654.3	13.77	12.51	10.11	10.82
Ccng2	NM_007635.4	655.91	631.55	579.12	643.73
Ccni	NM_017367.3	2706.86	2559.8	2767.14	2588.54
Ccr2	NM_009915.2	17.9	20.41	17.85	18.96

Ccr5	NM_009917.5	35.7	42.88	40.62	43.17
Cd109	NM_153098.3	19.23	33.46	22.71	32.07
Cd14	NM_009841.3	24.62	26.12	27.32	34.08
Cd163	NM_053094.2	18.38	21.33	18.94	25.33
Cd19	NM_009844.2	13.54	13.47	11.09	9.5
Cd209e	NM_130905.2	13.54	11.72	10.11	10.61
Cd244	NM_018729.2	13.54	14.36	12.24	10.93
Cd24a	NM_009846.2	73.74	74.79	55.69	66.19
Cd300lf	NM_001169153.1	15.08	13.76	17.63	17.25
Cd33	NM_001111058.1	50.94	59.4	58.91	65.24
Cd36	NM_007643.3	23.97	27.73	27.04	23.22
Cd3d	NM_013487.2	13.58	13.47	11.69	10.1
Cd3e	NM_007648.4	13.82	15.62	12.39	11.23
Cd3g	NM_009850.2	14.71	18.46	14.75	15.8
Cd40	NM_011611.2	24.49	26.99	23.42	22.81
Cd44	NM_009851.2	18.12	22.24	18.33	18.6
Cd47	NM_010581.3	2115.15	2029.7	2080.89	1997.77
Cd6	NM_001037801.2	65.65	64.11	76.69	49.71
Cd68	NM_009853.1	135.31	163.63	156.19	233.75
Cd69	NM_001033122.3	15.93	15.36	12.9	13.71
Cd70	NM_011617.1	13.91	15.52	12.29	11.96
Cd72	NM_001110320.1	33.7	35.43	23.29	41.59
Cd74	NM_001042605.1	32.46	62.61	74.18	127.86
Cd83	NM_009856.2	243.89	249.71	293.51	260.62
Cd84	NM_013489.2	56.99	67.35	68.21	91.7
Cd86	NM_019388.3	36.68	46.9	37.23	52.2
Cd8a	NM_001081110.2	13.54	11.72	11.13	9.5
Cd8b1	NM_009858.2	13.88	12.74	11.09	9.5
Cdc25a	NM_007658.3	98.45	100.39	104.43	95.64
Cdc7	NM_001271566.1	77.37	78.5	61.13	69.81
Cdk20	NM_053180.2	93.02	107.6	90	88.08
Cdkn1a	NM_007669.4	172.58	162.05	127.15	148.08
Cdkn1c	NM_009876.3	84.96	85.19	103.79	101.05
Ceacam3	NM_054059.1	13.91	12.21	12.91	11.08
Cflar	NM_207653.3	126.72	151	122.4	160.33
Ch25h	NM_009890.1	13.54	11.95	10.73	9.5
Chek1	NM_007691.5	19.7	19.24	17.97	17.78
Chek2	NM_016681.3	23.86	21.76	19.25	22.07
Chn2	NM_001163640.1	292.52	282.2	263.44	277.24
Chst8	NM_175140.4	107.61	116.52	136.04	119.29
Chuk	NM_001162410.1	585.62	558.43	473.96	501.02

Cidea	NM_007702.2	29.22	43.77	32.57	45.1
Cideb	NM_009894.3	54.91	52.5	56.59	58.64
Cks1b	NM_016904.1	37.04	42.14	43.96	45.17
Clcf1	NM_019952.3	14.05	13.13	10.98	10.17
Cldn5	NM_013805.4	354.07	227.31	200.76	256.77
Clec7a	NM_020008.2	13.58	17.46	13.84	36.12
Clic4	NM_013885.2	302.53	347.41	329.46	439.11
Cln3	NM_001146311.1	116.77	129.07	115.87	122.97
Clstn1	NM_023051.4	4653.17	4271.57	4768.08	4349.25
Cnn2	NM_007725.2	29.4	36.99	29.01	40.12
Cnp	NM_009923.2	3276.11	3679.87	3453.29	4568.59
Cntnap2	NM_001004357.2	524.83	515.08	555.17	639.79
Coa5	NM_198006.4	840.84	875.33	866.88	915.42
Col6a3	XM_897036.2	25.42	28.51	31.54	34.07
Cotl1	NM_028071.3	613.61	647.3	677.04	792.15
Cox5b	NM_009942.2	9067.72	9387.8	9331.5	9533.5
Cp	NM_001042611.1	116.67	134.11	117.86	191.25
Cpa3	NM_007753.2	13.92	12	10.27	10.9
Creb1	NM_001037726.1	386.81	387.06	392.05	374.55
Crebbp	NM_001025432.1	2122.82	2106.19	2058.26	1958.34
Crem	NM_001110853.1	204.67	211.3	187.72	168.21
Crip1	NM_007763.3	111.67	130.43	115.75	170.32
Cryba4	NM_021351.1	19.17	24.15	24.66	27.88
Csf1	NM_001113530.1	213	227.48	217.87	264.9
Csf1r	NM_001037859.1	478.29	543.08	462.81	578.58
Csf2rb	NM_007780.4	23.19	27.79	27.48	27.03
Csf3r	NM_001252651.1	35.97	46.51	34.8	50.24
Csk	NM_007783.2	284.4	303.11	251.89	306.12
Cst7	NM_009977.3	13.77	12.03	11.57	10.53
Ctse	NM_007799.3	13.54	15.27	12.52	12.29
Ctsf	NM_019861.1	798.53	851.13	841.83	901.93
Ctss	NM_021281.2	791.68	965.7	931.21	1360.67
Ctsw	NM_009985.4	13.54	12.74	11.7	9.75
Cx3cl1	NM_009142.3	1578.64	1484.06	1377.82	1302.3
Cx3cr1	NM_009987.3	556.71	596.2	617.62	745.85
Cxcl10	NM_021274.1	13.54	17.17	12.24	18.17
Cxcl9	NM_008599.2	13.89	15.35	10.61	11.57
Cycs	NM_007808.4	56.08	64.44	48.64	52.93
Cyp27a1	NM_024264.3	29.78	28.88	29.87	37.53
Cyp7b1	NM_007825.4	176.12	180.99	188.99	176.7
Cytip	NM_139200.4	14.18	13.13	12.23	11.22

Dab2	NM_023118.2	19.09	22.14	16.28	19.82
Dapk1	NM_134062.1	854.87	914.43	879.73	884.16
Ddb2	NM_028119.5	73.25	63.86	68.88	74.79
Ddx58	NM_172689.3	45.47	55.01	60.7	62.23
Dicer1	NM_148948.2	288.86	279.04	271.97	277.23
Dlg1	NM_001252433.1	1526.25	1465.72	1410.57	1415.05
Dlg4	NM_001109752.1	4310.13	4207.17	4386.71	4659.86
Dlx1	NM_010053.1	150.97	165.12	168.56	139.84
Dlx2	NM_010054.2	26.73	26.29	26.14	24.21
Dna2	NM_177372.3	25.33	26.64	21.04	21.06
Dnmt1	NM_010066.3	185.06	197.47	196.65	188.73
Dnmt3a	NM_007872.4	178.5	165.93	142.64	148.62
Dnmt3b	NM_001003960.3	13.54	13.78	10.11	9.5
Dock1	NM_001033420.2	194.62	201.32	177.66	219.4
Dock2	NM_033374.3	65.92	72.58	83.93	95.35
Dot1l	NM_199322.1	114.65	115.54	92.79	106.7
Dst	NM_010081.2	900.11	891.17	866.62	846.72
Duoxa1	NM_145395.2	13.54	12.08	10.89	10.27
Dusp7	NM_153459.4	1623.29	1476.01	1627.8	1492.37
E2f1	NM_007891.4	127.9	112.91	132.35	113.61
Eed	NM_021876.3	409.81	414.11	374.22	389.8
Eef2k	NM_007908.3	212.49	212.03	247.16	236.45
Egfr	NM_207655.2	77.6	90.13	73.04	73.45
Egr1	NM_007913.5	5401.83	4656.44	3234.23	2648.24
Ehmt2	NM_145830.1	2053.31	2076.55	2049.93	2162.57
Eif1	NM_011508.1	4870.06	4809.9	4915.41	4930.69
Emcn	NM_001163522.1	70.59	60.74	59.6	72.03
Emp1	NM_010128.4	19.71	29.97	24.04	30.9
Enpp6	NM_177304.3	44.89	49.14	34.43	49.58
Entpd2	NM_009849.2	85.08	96.36	86.77	102.15
Eomes	NM_010136.2	13.54	13.13	10.68	10.51
Ep300	NM_177821.6	930.28	880	889.36	925.17
Epcam	NM_008532.2	14.02	13.67	11.5	11.34
Epg5	NM_001195633.1	432.02	422.94	405.56	396.07
Epsti1	NM_029495.2	18.17	16.7	17.54	16.71
Erbb3	NM_010153.1	74.56	84.49	62.18	84.94
Ercc2	NM_007949.4	293.97	276.91	286.44	291.51
Esam	NM_027102.3	130.02	90.11	91.24	114.43
Ets2	NM_011809.2	976.22	1057.17	1023.93	1094.21
Exo1	NM_012012.4	13.68	15.06	13.38	11.58
Ezh1	NM_007970.1	191.79	190.48	207.82	187.69

Ezh2	NM_007971.2	231.34	240.34	213.75	223.13
F3	NM_010171.3	643.41	732.35	651.73	723.95
Fa2h	NM_178086.3	78.14	76.12	81.75	106.66
Fabp5	NM_010634.3	1568.21	1705.79	1690.57	1826.57
Fadd	NM_010175.5	27.31	30.56	29.18	33.54
Fancc	NM_007985.2	82.23	74.65	84.69	84.34
Fancd2	NM_001033244.3	13.9	14.14	13.25	13.98
Fancg	NM_053081.2	105.71	99.9	97.55	96.77
Fas	NM_007987.2	25.93	24.02	19.47	30.35
Fasl	NM_010177.3	15.12	14.14	13.33	12.32
Fbln5	NM_011812.4	34.56	32.48	30.26	45.83
Fcer1g	NM_010185.4	97.27	115.41	103.18	152.47
Fcgr1	NM_010186.5	64.24	68.95	80.28	89.98
Fcgr2b	NM_001077189.1	83.2	119.25	102.3	151.85
Fcgr3	NM_010188.5	77.8	99.73	81.59	112.2
Fcrla	NM_145141.2	15.3	18.26	12.77	12.37
Fcrlb	NM_001029984.2	13.54	11.95	10.92	10.76
Fcrls	NM_030707.3	205.83	239.89	201.08	239.73
Fdxr	NM_007997.1	16.19	17.39	14.51	19.41
Fen1	NM_001271614.1	137.76	135.53	144.25	159.88
Fgd2	NM_001159538.1	25.65	25.62	20.84	27.86
Fgf13	NM_010200.2	2357.29	2244.25	2322.97	2302.66
Fgl2	NM_008013.2	23.76	33.99	29.67	41.73
Fkbp5	NM_010220.3	124.8	134.82	136.61	167.9
Flt1	NM_010228.3	91.88	71.08	62.95	112.03
Fos	NM_010234.2	556.39	407.63	139.7	181.03
Foxp3	NM_054039.2	14.23	14.74	14.51	12.04
Fpr1	NM_013521.2	13.54	13.36	11.09	9.75
Fscn1	NM_007984.2	2095.82	1905.72	2015.76	2163.41
Fyn	NM_008054.2	1351.29	1360.77	1342.15	1348.98
Gadd45a	NM_007836.1	202.51	174.07	122.49	121.08
Gadd45g	NM_011817.2	104.43	110.81	79.89	108.3
Gal3st1	NM_001177691.1	82.57	84.41	81.01	107.44
Gba	NM_001077411.1	227.34	211.91	222.9	222.41
Gbp2	NM_010260.1	31.1	37.65	29.72	55.32
Gclc	NM_010295.2	361.6	383.35	361.69	380.85
Gdpd2	NM_023608.3	49.22	62.38	43.51	55.68
Gja1	NM_010288.3	2746.34	3223.25	2630.77	3071.13
Gjb1	NM_008124.2	116.56	124.31	124.98	194.54
Gna15	NM_010304.3	33.02	31.64	29.67	34.19
Gpr183	NM_183031.2	20.69	30.14	25.61	30.17

Gpr34	NM_011823.4	219.39	221.72	207.87	251.67
Gpr62	NM_001159652.1	103.58	105.39	121.36	115.85
Gpr84	NM_030720.1	17.92	18.69	16.11	18.03
Grap	NM_027817.3	41.85	40.5	38.12	46.97
Gria1	NM_001252403.1	2066.8	1978.71	2060.34	2304.46
Gria2	NM_001039195.1	4835.92	4383.31	4311.1	4307.72
Gria4	NM_001113180.1	1802.96	1712.12	1771.04	1553.78
Grin2a	NM_008170.2	469.54	431.13	481.66	425.74
Grin2b	NM_008171.3	3160.07	2905.88	2973.24	2900.63
Grm2	NM_001160353.1	458.67	431.31	490.95	364.41
Grm3	NM_181850.2	2368.4	2262.31	2190.83	2120.05
Grn	NM_008175.3	455.7	481.83	435.36	552.86
Gsn	NM_146120.3	369.05	410.2	358.81	480.13
Gstm1	NM_010358.5	44.96	51.49	50.1	60.42
Gzma	NM_010370.2	13.54	12.3	10.11	9.5
Gzmb	NM_013542.2	14.74	15.16	11.67	9.5
H2afx	NM_010436.2	821.02	772.1	801.98	818.21
H2-T23	NM_010398.3	95.87	82.62	99.02	102.86
Hat1	NM_026115.4	481.86	510.59	505.44	506.11
Hcar2	NM_030701.1	14.65	14.77	12.9	15.4
Hdac1	NM_008228.2	142.12	154.7	139.74	146.21
Hdac2	NM_008229.2	1084.02	1129.29	1027.21	1005.93
Hdac4	NM_207225.1	286.6	292.09	284.03	274.33
Hdac6	NM_010413.3	200.05	185.78	170.73	190.11
Hdc	NM_008230.4	15.96	19.13	13.58	13.47
Hells	NM_008234.3	33.2	39.63	34.52	35.86
Hif1a	NM_010431.2	1799.2	1824.45	1961.56	1894.82
Hilpda	NM_023516.5	60.25	55.8	51.43	54.58
Hira	NM_010435.2	475.51	428.17	466.15	435.62
Hist1h1d	NM_145713.3	13.54	12.1	11.42	11.56
Hmgb1	NM_010439.3	1858.29	1931.69	1970.23	1828.63
Hmox1	NM_010442.2	41.42	45.94	46.31	51.67
Homer1	NM_147176.2	4382.34	4053.56	3583.55	3145.37
Hpgds	NM_019455.4	79.2	82.22	90.22	89.71
Hprt	NM_013556.2	1015.19	1002.81	1126.25	1108.7
Hps4	NM_138646.3	89.16	102.95	95.29	91.2
Hrk	NM_007545.2	153.9	200.87	142.57	204.39
Hsd11b1	NM_008288.2	413.87	400.82	475.43	333.68
Hspb1	NM_013560.2	51.02	51.45	36.34	65.36
Hus1	NM_008316.2	163.61	168.14	182.37	177.11
Icam2	NM_010494.1	46.92	33.4	31.8	32.16

Ifi30	NM_023065.3	41.83	50.54	47.48	73.19
Ifih1	NM_027835.2	120.07	128.78	150.4	136.12
Ifitm2	NM_030694.1	194.65	186.63	226.68	248.06
Ifitm3	NM_025378.2	108.29	161.05	153.58	284.74
Ifnar1	NM_010508.1	526.89	498.46	470.16	495.54
Ifnar2	NM_001110498.1	169.35	168.86	173.66	193.33
Igf1	NM_001111274.1	20.63	24.89	26.05	23.54
Igf1r	NM_010513.2	387.56	365.25	394.36	396.58
Igf2r	NM_010515.1	308.3	299.42	296.86	312.75
Igsf10	NM_001162884.1	34.44	43.53	36.9	37.12
Igsf6	NM_030691.1	16.13	18.62	17.74	23.43
Ikbkb	NM_010546.2	327.73	299.2	301.98	288.92
Ikbke	NM_019777.3	16.06	16.88	15.42	17.64
Ikbkg	NM_178590.2	217.09	222.28	226.15	232.76
Il10rb	NM_008349.5	147.42	144.81	143.09	154.13
Il15ra	NM_008358.2	35.09	40.82	36.86	29.4
Il1a	NM_010554.4	15.82	17.41	15.85	16.91
Il1b	NM_008361.3	15.5	13.59	12.09	13.86
Il1r1	NM_001123382.1	66.77	70.89	71.5	83.32
Il1r2	NM_010555.4	13.54	15.36	12.48	9.5
Il1rap	NM_134103.2	325.19	305.45	321.34	292.1
Il1rl2	NM_133193.3	19.58	27.73	25.02	28.28
Il1rn	NM_031167.5	13.54	15.25	11.53	10.32
Il21r	NM_021887.1	15.13	18.38	15.65	17.22
Il2rg	NM_013563.3	26.04	27	23.68	29.57
Il3	NM_010556.4	17.44	16.05	18.29	14.96
Il3ra	NM_008369.1	13.78	14.63	10.12	10.45
Il6ra	NM_010559.2	52.39	53.8	49.32	52.19
Inpp5d	NM_001110192.1	39.78	41.39	43.24	56.47
Iqsec1	NM_001134383.1	2617.36	2409.5	2608.88	2348.75
Irak1	NM_008363.2	166.62	176.71	165.16	171.76
Irak2	NM_001113553.1	209.99	208.29	217.53	235.41
Irak3	NM_028679.3	25.4	31.39	33.43	37.99
Irak4	NM_029926.5	33.84	36.35	31.21	39.22
Irf1	NM_008390.1	78.36	74.85	69.03	79.04
Irf2	NM_008391.2	515.12	501.69	503.95	464.46
Irf3	NM_016849.4	126.72	131.54	111.42	126.21
Irf4	NM_013674.1	17.5	18.57	18.13	14.84
Irf6	NM_016851.2	16.27	15.91	17.53	17.41
Irf7	NM_016850.2	27.35	23.95	28.7	42.25
Irf8	NM_008320.3	44.47	51.09	40.8	59.31

Islr2	NM_001161538.1	444.43	550.27	406.64	484.71
Itga6	NM_008397.3	252.53	241.86	225.16	258.78
Itga7	NM_008398.2	57.25	55.23	58.8	62.53
Itgam	NM_001082960.1	93.42	117.73	103.27	137.32
Itgav	NM_008402.2	796.98	690.06	787.72	820.53
Itgax	NM_021334.2	13.54	14.74	12.08	11.32
Itgb5	NM_001145884.1	445.69	527.54	487.45	593.96
Jag1	NM_013822.2	59.61	48.88	51.1	59.05
Jam2	NM_023844.4	319.9	319.82	320.26	340.13
Jarid2	NM_021878.2	258.94	257.09	238.34	265.56
Jun	NM_010591.2	463.49	512.65	392.34	487.03
Kat2a	NM_020004.5	578.81	542.4	570.55	597.21
Kat2b	NM_020005.3	260.24	257.04	255.09	293.99
Kcnd1	NM_008423.1	53.56	52.29	46.57	57.08
Kcnj10	NM_001039484.1	746.82	707.39	720.75	779.53
Kcnk13	NM_146037.1	52.18	59.18	60.01	62.61
Kdm1a	NM_133872.1	863.88	879.11	834.85	815.71
Kdm1b	NM_172262.3	91.64	100.23	84.81	87.87
Kdm2a	NM_001001984.2	588.94	552	521.06	568.33
Kdm2b	NM_001003953.1	136.74	128.67	129.4	136.67
Kdm3a	NM_001038695.2	428.18	421.79	347.18	403.56
Kdm3b	NM_001081256.1	16.64	18.2	18.92	18.69
Kdm4a	NM_172382.2	210.31	212.01	223.18	250.56
Kdm4b	NM_172132.1	471.28	462.48	449.62	503.53
Kdm4c	NM_144787.1	328.01	299.68	278.94	313.23
Kdm4d	NM_173433.2	30.97	22.78	35.96	30.49
Kdm5a	XR_377436.1	414.37	418.28	405.03	426.1
Kdm5b	NM_152895.2	748.89	749.24	684.49	682.62
Kdm5c	NM_013668.3	482.07	456.73	448	446.27
Kdm5d	NM_011419.3	213.85	208.36	194.69	201.06
Kdm6a	NM_009483.1	167.89	149.48	166.47	175.39
Kif2c	NM_134471.3	14.24	15.45	10.92	12.59
Kir3dl1	NM_177749.3	13.93	11.95	11.51	10.14
Kir3dl2	NM_177748.2	24.07	26.22	19.17	21.15
Kit	NM_001122733.1	134.28	149.55	139.72	141.18
Klrb1	NM_001099918.1	14.61	11.8	13.82	10.75
Klrd1	NM_010654.2	13.54	13.78	10.91	9.5
Klrk1	NM_001083322.1	19.18	23.88	17.59	18.97
Kmt2a	NM_001081049.1	548.01	540.13	521.24	535.66
Kmt2c	NM_001081383.1	407.69	402.3	392.48	425.16
Lacc1	NM_172488.2	66.29	74.89	62.08	76.94

Lag3	NM_008479.1	52.82	61.14	78.21	94.35
Lair1	NM_001113474.1	94.29	98.26	115.95	126.39
Lamp1	NM_010684.2	3703.94	3906.66	4050.46	4448.25
Lamp2	NM_001017959.1	1649.47	1762.26	1791.25	1882.01
Lcn2	NM_008491.1	13.79	13.58	20.74	45.62
Ldha	NM_010699.2	3937.37	3827.11	3852.18	4093.87
Ldlrad3	NM_178886.2	116.76	116.66	105.44	138.42
Lfng	NM_008494.3	233.23	250.73	222.67	277.92
Lgmn	NM_011175.3	1309.64	1402.09	1347.28	1461.72
Lig1	NM_001083188.1	102.58	91.36	105.34	102.36
Lilrb4a	NM_013532.3	19.55	33.62	25.31	43.27
Lingo1	NM_181074.4	2845.89	2610.47	2817.72	2451.33
Lmna	NM_001002011.2	523.89	483.6	549.59	505.74
Lmnb1	NM_010721.2	109.2	98.2	108.12	95.02
Lrg1	NM_029796.2	17.57	22.16	17.19	22.35
Lrrc25	NM_153074.3	14.46	15.91	13.19	16.44
Lrrc3	NM_145152.4	155.64	165.78	154.83	187.2
Lsr	NM_001164184.1	69.52	59.83	44.52	72.89
Lst1	NM_010734.2	19.98	20.26	15.17	16.14
Lta	NM_010735.2	16.13	16.64	13.21	14.28
Ltb	NM_008518.2	19.49	16.26	15.7	18.06
Ltbr	NM_010736.3	41.64	50.33	45.04	56.22
Ltc4s	NM_008521.1	15.13	18.52	14.13	18.29
Ly6a	NM_010738.2	201.29	175.15	235.27	354.93
Ly6g	XM_909927.2	14.77	13.78	10.11	10.31
Ly9	NM_008534.2	21.52	29.47	29.52	34.81
Lyn	NM_010747.1	59.53	62.77	64.78	79.61
Mafb	NM_010658.2	472.36	444.68	504.69	475.86
Maff	NM_010755.3	16.1	17.7	14	20.36
Mag	NM_010758.2	208	221.52	202.26	285.93
Mal	NM_001171187.1	1067.76	1199.27	1099.91	1450.87
Man2b1	NM_010764.2	376.92	395.91	425.99	475.9
Map1lc3a	NM_025735.1	1868.37	1735.39	1963.4	1931.08
Map2k1	NM_008927.3	4402.86	4530.37	4435.94	4831.98
Map2k4	NM_009157.4	2123.63	2061.47	2055.93	1934.66
Map3k1	NM_011945.2	99.96	90.5	90.14	100.47
Map3k14	NM_016896.3	30.74	26.39	25.1	20.54
Mapk10	NM_001081567.1	4939.55	4987.86	4983.97	4402.97
Mapk12	NM_013871.3	21.83	21.37	17.09	26.69
Mapk14	NM_011951.2	671.1	699.74	689.96	643.81
Mapt	NM_001038609.2	1824.49	1738.87	1803.18	1766.24

Marco	NM_010766.2	17.4	17.17	19.38	17.6
Mavs	NM_144888.2	78.23	71.66	77.05	90.24
Mb21d1	NM_173386.4	18.2	17.57	20.89	19.12
Mbd2	NM_010773.2	1383.46	1405.18	1318.22	1507.84
Mbd3	NM_013595.2	1013.55	976.34	953.63	1042.24
Mcm2	NM_008564.2	36.62	33.52	35.85	36.24
Mcm5	NM_008566.2	77.28	72.91	79.37	70
Mcm6	NM_008567.1	55.64	62.61	64.98	64.17
Mdc1	NM_001010833.2	133.43	124.95	128.04	133.13
Mdm2	NM_010786.4	688.25	634.98	613.78	642.59
Mef2c	NM_001170537.1	10330.65	9099.02	10933.65	8358.61
Mertk	NM_008587.1	354.15	382.35	385.12	432.05
Mfge8	NM_008594.2	266.55	266.76	254.1	276.19
Mgmt	NM_008598.2	25.4	28.08	31.54	28.94
Mmp12	NM_008605.3	13.54	16.69	10.66	22.31
Mmp14	NM_008608.3	47.8	63.94	39.83	61.67
Mobp	NM_001039364.2	2487.35	2644.86	2428.7	3467.76
Mog	NM_010814.2	438.35	489.11	420.31	597.49
Mpeg1	NM_010821.1	230.72	276.61	310.42	406.28
Mpg	NM_010822.3	44.31	43.73	51.45	49.79
Mr1	NM_008209.4	76.15	90.04	100.99	94.26
Mre11a	NM_018736.2	213.54	213.05	205.38	217.25
Ms4a1	NM_007641.5	13.54	11.72	10.11	9.5
Ms4a2	NM_001276330.1	13.54	11.72	10.61	9.5
Ms4a4a	XM_003086124.1	15.08	18.48	19.69	19.81
Msh2	NM_008628.2	435.51	434.42	453.53	420.66
Msn	NM_010833.2	60.04	72.68	68.41	98.92
Msr1	NM_001113326.1	15.84	12.86	13.84	13.39
Mvp	NM_080638.2	49.04	61.65	56.39	73.95
Myc	NM_010849.4	49.63	55.92	44.33	50.79
Myct1	NM_026793.2	26.35	25.69	29.5	23.34
Myd88	NM_010851.2	49.7	48.56	47.54	53.72
Myrf	NM_001033481.1	366.28	363.06	338.6	501.38
Nbn	NM_013752.3	166.15	185.63	169.43	160.59
Ncaph	NM_144818.3	21.75	20.06	17.64	23.02
Ncf1	NM_001286037.1	38.04	43.71	48.18	68.44
Ncor1	NM_011308.2	1160.11	1090.89	1153.98	1110.88
Ncor2	NM_011424.2	695.86	660.7	684.33	687.62
Ncr1	NM_010746.3	13.54	11.72	10.11	9.5
Nefl	NM_010910.1	1428.04	1231.8	1694.67	1475.51
Nfe2l2	NR_132727.1	40.02	43.44	44.26	52.95

Nfkb1	NM_008689.2	198.23	198.38	174.36	200.83
Nfkb2	NM_019408.2	20.14	22.7	14.73	25.79
Nfkbia	NM_010907.2	67.12	67.61	60.28	87.36
Nfkbie	NM_008690.3	28.74	27.72	32.6	23.41
Ngf	NM_001112698.1	65.61	65.4	61.96	52.24
Ngfr	NM_033217.3	15.7	16.89	19.6	24.08
Ninj2	NM_016718.2	23.14	21.25	17.81	24.04
Nkg7	NM_024253.4	15.58	17.13	12.85	10.88
Nlgn1	NM_138666.3	1476.89	1575.7	1404.88	1523.83
Nlgn2	NM_198862.2	2938.49	2700.43	3018.83	2977.05
Nlrp3	NM_145827.3	15.92	14.15	16.42	16.77
Nod1	NM_172729.2	39.16	36.59	40.42	44.96
Nostrin	NM_181547.3	43.97	45.49	39.15	55.79
Npl	NM_028749.1	75.18	91.83	81.89	96.09
Npnt	NM_001029836.1	368.37	291.24	341.96	259.85
Nptx1	NM_008730.2	5130.26	4887.52	5171.53	4534.26
Nqo1	NM_008706.5	56.91	77.51	74.15	80.2
Nrgn	NM_022029.2	9898.27	8158.18	9702.72	8588.96
Nrm	NM_134122.2	41.84	36.31	34.9	37.52
Nrp2	NM_001077403.1	127.16	165.64	105.13	188.12
Nthl1	NM_008743.2	39.56	39.49	37.5	33.71
Nwd1	NM_176940.5	370.17	365.01	377.5	388.04
Oas1g	NM_011852.2	19.84	22.65	17.22	24.02
Ogg1	NM_010957.4	48.7	48.88	52.25	60.39
Olfml3	NM_133859.2	155.84	170.5	158.97	186.77
Opalin	NM_153520.1	422.01	460.53	449.09	546.95
Optn	NM_181848.4	192.56	213.57	226.87	217.07
Osgin1	NM_027950.1	16.28	17.13	18.02	17.36
Osmr	NM_011019.3	34.13	48.87	43.16	55.86
P2rx7	NM_001038839.2	108.81	116.57	109.6	139.76
P2ry12	NM_027571.3	388.12	393.18	422.99	474.12
Pacsin1	NM_011861.3	3200.6	2872.64	3206.53	3063.52
Padi2	NM_008812.2	130.04	180.48	131.61	197.14
Pak1	NM_011035.2	7898.9	7616.25	8131.77	7067.56
Parp1	NM_007415.2	514.86	492.02	497.16	493.38
Parp2	NM_009632.2	259.71	251.85	261.43	252.28
Pcna	NM_011045.2	764.08	812.13	794.75	752.91
Pdpn	NM_010329.2	175.26	226.57	197.31	258.31
Pecam1	NM_008816.2	185.33	139.64	143.29	172.49
Pex14	NM_019781.2	478.16	423.28	480.73	441.57
Pik3ca	NM_008839.1	821.46	840.23	787.76	729.55

Pik3cb	NM_029094.3	589.56	568.96	582.42	556.24
Pik3cd	XM_003945690.1	134.22	128.47	120.1	117.33
Pik3cg	NM_020272.2	31.1	36.67	34.19	42.32
Pik3r1	NM_001024955.1	1027.54	1017.44	1064.38	1052.05
Pik3r2	NM_008841.2	1239.67	1134.85	1186.02	1158.77
Pik3r5	NM_177320.2	18.28	15.45	15.59	15.74
Pilra	NM_153510.3	14.37	15.25	12.59	11.61
Pilrb1	NM_133209.2	13.54	12.74	10.11	9.83
Pink1	NM_026880.2	4125.89	4106.35	4402.44	4333.32
Pla2g4a	NM_008869.2	30.64	28.92	30.4	35.69
Pla2g5	NM_001122954.1	36.93	42.9	37.4	39.55
Plcg2	NM_172285.1	58.2	64.74	61.4	80.19
Pld1	NM_001164056.1	49.45	51.22	47.12	51.22
Pld2	NM_008876.2	51.79	52.43	50.76	62.66
Plekhb1	NM_001163184.1	2950.54	3272.23	2858.53	3767.51
Plekhm1	NM_183034.1	179.23	186.19	180.81	183.99
Pllp	NM_026385.3	104.01	108.26	109.22	136.64
Plp1	NM_011123.2	7510.37	8361.27	7048.78	10492.31
Plxdc2	NM_026162.5	571.32	628.11	558.18	643.22
Plxnb3	NM_019587.2	93.62	95.74	88.18	125.89
Pmp22	NM_008885.2	137.03	135.23	161.62	237.62
Pms2	NM_008886.2	346.44	314.49	361.27	341.14
Pnoc	NM_001205075.1	30.31	34.84	40.43	34.04
Pole	NM_011132.2	22.76	20.29	26.06	22.78
Ppfia4	NM_001144855.1	180.27	186.92	184.78	211.55
Ppp3ca	NM_008913.4	17398.1	16471.4	16999.08	16581.47
Ppp3cb	NM_008914.2	4673.98	4437.67	4583.92	4400.05
Ppp3r1	NM_024459.2	6799.72	6423.3	7007.16	6316.67
Ppp3r2	NM_001004025.4	21.56	21.89	14.98	17.55
Prdx1	NM_011034.4	211.15	216.15	225	230.63
Prf1	NM_011073.2	13.54	14.74	11.64	9.75
Prkaca	NM_008854.3	3602.36	3436.49	3621.93	3513.93
Prkacb	NM_011100.3	4937.63	4768.31	4958.53	4829.6
Prkar1a	NM_021880.2	6831.16	6731.84	7325.2	7356.16
Prkar2a	NM_008924.2	755.7	737.63	743.6	759.83
Prkar2b	NM_011158.3	1130.97	1160.6	978.58	1144.46
Prkce	NM_011104.2	4482.92	4382.63	4559.37	4585.63
Prkcq	NM_008859.2	73.62	81.38	64.99	90.3
Prkdc	NM_011159.2	195.65	203.19	179.27	188.08
Pros1	NM_011173.2	85.57	90.79	81.67	106.99
Psen2	NM_001128605.1	121.93	128.46	126.28	126.78

Psmb8	NM_010724.2	65.68	82.3	88.69	123.81
Pten	NM_008960.2	3112.44	3046.99	3164.15	2991.36
Ptger3	NM_011196.2	19.33	24.18	23.83	22.42
Ptger4	NM_008965.1	17.27	19.36	13.93	18.2
Ptgs2	NM_011198.3	173.67	198.02	144.62	145.89
Ptms	NM_026988.2	6290.75	5937.82	6199.23	6746.9
Ptpn6	NM_013545.2	41.9	45.46	49.14	61.79
Ptprc	NM_011210.3	38.38	45.33	48.03	66.07
Pttg1	NM_001131054.1	708.24	707.43	776.35	688.9
Ptx3	NM_008987.3	15.95	17.58	11.37	18.37
Pycard	NM_023258.4	15.14	13.2	10.11	11.88
Rab6b	NM_173781.4	5463.71	5235.25	5374.02	5148.05
Rab7	NM_009005.2	4293.44	4053.05	4359.43	4221.46
Rac1	NM_009007.2	3440.64	3631.58	3579.85	3584.02
Rac2	NM_009008.3	24.48	27.66	27.27	21.02
Rad1	NM_011232.2	201.63	195.92	196.41	185.44
Rad17	NM_001044371.1	138.93	135.84	136.89	147.66
Rad50	NM_009012.2	140.77	140.13	149.26	139.61
Rad51	NM_011234.4	23.26	27.68	28.54	22
Rad51b	NM_009014.3	14.34	15.38	15.49	11.72
Rad51c	NM_053269.3	43.79	47.34	49.29	48.63
Rad9a	NM_011237.2	51.36	55.03	47.52	61.44
Rag1	NM_009019.2	13.54	14.03	12	11.29
Rala	NM_019491.5	789.9	839.03	754.82	754.51
Ralb	NM_022327.5	291.79	293.58	282.27	301.04
Rapgef3	NM_001177810.1	190.38	190.42	185.91	230.35
Rb1cc1	NM_009826.4	1256.03	1233.83	1272.04	1174.33
Rbfox3	NM_001024931.2	3679.49	3259.85	3592.79	3360.14
Rela	NM_009045.4	122.7	127.45	121.26	136.79
Relb	NM_009046.2	102.82	106.83	108.78	110.16
Reln	NM_011261.2	309.68	333.69	319.12	355.53
Rgl1	NM_016846.3	372.86	406.11	370.92	428.53
Rhoa	NM_016802.4	1565.41	1579.89	1718.84	1845.69
Ripk1	NM_009068.3	28.99	33.4	28.91	29.24
Ripk2	NM_138952.3	90.81	80.15	82.68	90.48
Rnf8	NM_021419.2	387.92	358.52	371.07	382.46
Rpa1	NM_026653.2	512.98	514.28	527.36	529.3
Rpl28	NM_009081.2	3841.13	4031.6	3891.92	4171.54
Rpl29	NM_009082.2	2441.82	2599.74	2516.35	2514.67
Rpl36al	NM_025589.4	898.16	972.39	946.04	931.67
Rpl9	NM_011292.2	3730.32	3894.23	3781.84	3883.62

Rps10	NM_025963.3	850.86	880.73	853.78	1007.31
Rps2	NM_008503.5	3279.01	3281.32	3228.29	3552.23
Rps21	NM_025587.2	10013	10259.26	10572.64	11294.43
Rps3	NM_012052.2	226.79	220.89	232.11	207.35
Rps9	NM_029767.2	3237.48	3600.83	3327.53	3808
Rrm2	NM_009104.1	16.81	20.44	17.03	17.58
Rsad2	NM_021384.2	45.91	55.39	55.45	64.17
Rtn4rl1	NM_177708.4	516.72	508.92	572.75	528.69
S100a10	NM_009112.2	918.47	951.44	929.57	975.03
S100b	NM_009115.3	1023.53	1086.7	1174.11	1270.53
S1pr3	NM_010101.3	117.88	124.87	104.33	136.08
S1pr4	NM_010102.2	13.54	13.13	10.11	10.93
S1pr5	NM_053190.2	59.7	64.79	68.13	81.53
Sall1	NM_021390.3	276.63	311.92	282.23	326.98
Sell	XM_006496716.1	13.93	13.47	13.49	11.03
Serpina3n	NM_009252.2	47.78	74.55	60.01	120.37
Serpine1	NM_008871.2	15.53	20.88	17.1	22.56
Serpinf1	NM_011340.3	86.6	116.3	86.49	122.52
Serping1	NM_009776.3	26.88	48.68	35.61	56.39
Sesn1	NM_001013370.2	556.95	599.04	627.18	572.99
Sesn2	NM_144907.1	79.18	70.77	84.3	82.14
Setd1a	NM_178029.3	165.64	151.58	149.95	167.5
Setd1b	NM_001040398.1	516.95	501.95	498.65	512.54
Setd2	NM_001081340.2	1196.43	1137.27	1129.16	1142.33
Setd7	NM_080793.5	517.79	475.85	488.66	501
Setdb1	NM_018877.2	133.61	133.3	108.39	139.91
Sftpd	NM_009160.2	19.83	22.87	19.72	16.69
Sh2d1a	NR_132588.1	13.54	13.13	10.11	9.6
Shank3	NM_021423.3	854.66	792.53	779.73	787.31
Siglec1	NM_011426.3	14.15	11.72	12.53	9.5
Siglecf	NM_145581.1	14.2	13.69	16.75	17.28
Sin3a	NM_001110350.1	453.04	452.34	434.12	434.64
Sirt1	NM_019812.2	263.06	264.15	228.86	250.22
Slamf8	NM_029084.3	14.23	13.84	13.46	14.94
Slamf9	NM_029612.4	26.9	32.84	41.05	48.63
Slc10a6	NM_029415.2	13.72	16.91	13.24	15.88
Slc17a6	NM_080853.3	478.16	506.79	469.1	482.84
Slc17a7	NM_182993.2	7911.62	7519.13	8764.67	8176.93
Slc1a3	NM_148938.3	4378.56	4941.89	4611.41	4943.55
Slc2a1	NM_011400.3	190.14	177.68	185.32	234.37
Slc2a5	NM_019741.3	57.42	62.67	64.93	78.72

Slc44a1	NM_001159633.1	888.75	917.44	905.94	1037.87
Slc6a1	NM_178703.4	2300.28	2153.25	2530.72	2473.91
Slco2b1	NM_175316.3	95.07	98.43	93.79	112.24
Slfn8	NM_181545.4	27.98	28.44	24.25	31.82
Smarca4	NM_011417.2	1113.47	1046.24	1009.02	978.63
Smarca5	NM_053124.2	1612.17	1684.2	1627.02	1608.68
Smarcd1	NM_031842.1	570.5	553.1	566.35	638
Smc1a	NM_019710.2	608.84	607.21	590.61	593.54
Snca	NM_009221.2	2112.43	2282.76	2154.9	2110.5
Socs3	NM_007707.2	36.55	32.54	32.05	37.07
Sod2	NM_013671.3	3044.73	3008.13	3167.05	2918.12
Sox10	XM_128139.6	137.19	150.27	133.36	183.1
Sox4	NM_009238.2	162.8	173.29	126.02	139.85
Sox9	NM_011448.4	414.83	429.66	400.97	474.38
Sphk1	NM_011451.3	19.03	18.61	18.33	15.39
Spib	NM_019866.1	15.49	16.34	13.78	11.4
Spint1	NM_016907.3	22.66	17.2	14.4	26.53
Spp1	NM_009263.3	111.42	211.54	135.96	275.84
Sqstm1	NM_011018.2	2381.07	2345.26	2552.25	2557.76
Srgn	NM_011157.2	93.79	109.64	101.49	139.91
Srxn1	NM_029688.4	858.6	790.02	850.69	901.27
St3gal6	NM_018784.2	207.26	207.38	202.58	206.38
St8sia6	NM_145838.1	26.18	31.84	30.46	43.22
Stat1	NM_009283.3	156.79	162.27	185.36	207.89
Steap4	NM_054098.3	13.89	14	11.42	12.29
Stmn1	NM_019641.3	14621.46	15052.89	14651.65	13419.06
Stx18	NM_026959.2	113.84	114.18	115.75	110.14
Sumo1	NM_009460.1	3484.11	3401.54	3626.05	3447.94
Suv39h1	NM_011514.2	370.25	386.75	390.52	385.74
Suv39h2	NM_022724.4	96.72	91.57	77.35	82.52
Suz12	NM_199196.1	818.98	826.96	817.36	845.63
Syk	NM_001198977.1	39.18	45.66	41.71	41.48
Syn2	NM_013681.1	4525.48	4703.75	4757.62	4899.56
Syp	NM_009305.2	8499.62	8396.7	9485.9	9104.95
Tarbp2	NM_001253795.1	144.8	138.76	127.61	145.85
Tbc1d4	NM_001081278.2	59.12	64.94	54.95	63.46
Tbr1	NM_009322.3	738.14	644.06	712.24	635.27
Tbx21	NM_019507.2	13.54	11.72	10.11	9.5
Tcirg1	NM_001136091.1	52.29	64.08	58.15	73.08
Tcl1	NM_009337.3	13.54	11.72	10.11	9.5
Tet1	NM_027384.1	215.66	216.58	190.19	193.14

Tfg	NM_001252443.1	1272.49	1257.72	1238.05	1211.43
Tgfa	NM_031199.2	259.77	278.5	207.22	359.65
Tgfb1	NM_011577.1	60.02	68.43	59.91	78.25
Tgfbr1	NM_009370.2	387.22	429.17	401.83	448.48
Tgm1	NM_001161714.1	13.54	14.64	10.49	11.75
Tgm2	NM_009373.3	48.85	48.63	52.11	64.8
Tie1	NM_011587.2	58.19	45.08	40.59	49.58
Timeless	NM_011589.1	55.42	51.56	53.84	53.62
Timp1	NM_011593.2	13.54	13.13	10.11	10.56
Tle3	NM_009389.2	285.77	298.06	315.52	318.42
Tlr2	NM_011905.2	13.54	15.67	12.83	16.21
Tlr4	NM_021297.2	32.32	36.43	30.42	37.15
Tlr7	NM_133211.3	21.61	26.3	23.58	28.53
Tm4sf1	NM_008536.3	120.86	132.7	111.45	150.34
Tmc7	NM_172476.4	102.3	91.29	91.11	94.01
Tmcc3	NM_172051.2	324.7	370.26	334.38	414.27
Tmem100	NM_026433.2	167.56	174.31	149.87	172.66
Tmem119	NM_146162.2	259.47	287.56	289.35	333.88
Tmem144	NM_027495.4	117.16	117.64	120.4	134.19
Tmem173	NM_028261.1	21.77	25.34	17.01	23.74
Tmem204	NM_001001183.1	38.07	31.59	33.42	35.95
Tmem206	NM_025864.3	172.67	185.22	170.48	174.36
Tmem37	NM_019432.2	24.29	24.68	31.33	27.65
Tmem64	NM_181401.3	1126.74	1159.56	1036.2	1009.95
Tmem88b	NM_001033394.3	220.27	228.39	213.31	281.15
Tnf	NM_013693.2	13.54	13.78	10.11	9.63
Tnfrsf10b	NM_020275.3	16.48	15.18	15.8	15.25
Tnfrsf11b	NM_008764.3	23.07	26.85	22.41	20.84
Tnfrsf12a	NM_001161746.1	95.6	86.23	83.11	77.84
Tnfrsf13c	NM_028075.2	15.31	18.57	13.54	14.52
Tnfrsf17	NM_011608.1	15.81	17.59	20.68	18.56
Tnfrsf1a	NM_011609.2	88.4	103.41	97.04	123.97
Tnfrsf1b	NM_011610.3	22.76	32.04	25.17	30.02
Tnfrsf25	NM_033042.3	36.7	26.99	16.87	19.66
Tnfrsf4	NM_011659.2	13.54	11.95	10.11	9.94
Tnfsf10	NM_009425.2	32.25	23.15	27.75	33.24
Tnfsf12	NM_011614.3	76.36	83.37	79.78	92.42
Tnfsf13b	NM_033622.1	17.37	16.97	16.57	22.42
Tnfsf4	NM_009452.2	13.54	12.74	10.11	9.83
Tnfsf8	NM_009403.2	14.02	13.13	11.69	10.27
Top2a	NM_011623.2	21.75	23.73	19.83	18.69

Topbp1	NM_176979.5	93.07	100.67	100.65	97.34
Tpd52	NM_001025262.1	1731.27	1669.28	1845.51	1753.17
Tpsb2	NM_010781.3	13.93	11.72	10.12	10.45
Tradd	NM_001033161.2	36.18	34.22	32.01	37.29
Traf1	NM_009421.3	20.35	26.24	17.91	21.86
Traf2	NM_009422.2	165.25	165.51	152.87	163.19
Traf3	NM_011632.3	254.66	273.74	237.69	229.93
Traf6	NM_009424.2	178.39	177.15	175.81	188.53
Trat1	NM_198297.3	18.14	19.13	14.69	19.88
Trem1	NM_021406.5	13.54	13.87	11.09	10.76
Trem2	NM_031254.2	172.62	200.45	224.29	306.97
Trem3	NM_021407.3	14.26	12.43	12.52	10.46
Trim47	NM_001205081.1	67.84	61.7	56.97	71.95
Trp53	NM_011640.1	102.63	105.41	95.63	109.45
Trp53bp2	NM_173378.2	147.53	153.66	150.55	175.27
Trp73	NM_011642.3	14.21	20.14	13.5	16.09
Trpa1	NM_177781.4	14.09	13.64	11.52	11.78
Trpm4	NM_175130.4	61.1	45.06	56.31	53.35
Tspan18	NM_183180.2	16.99	22.97	18.21	25.55
Ttr	NM_013697.4	69.97	75.27	46.74	134.56
Tubb3	NM_023279.2	4087.71	4112.69	4188.44	4194.37
Tubb4a	NM_009451.3	1453.82	1415.55	1491.16	1612.84
Txnrd1	NM_015762.2	770.43	757.16	740.56	805.28
Tyrobp	NM_011662.2	295.77	376.82	381.28	523.14
Ugt8a	NM_011674.4	346.68	355.05	290.23	371.58
Ulk1	NM_009469.3	910.9	864.2	925.31	866.58
Ung	NM_001040691.1	37.72	46.56	39.17	35.95
Uty	NM_009484.2	262.41	264.43	240.95	235.48
Vamp7	NM_011515.4	1082.67	1101.49	1167.18	1091.3
Vav1	NM_011691.4	67.03	72.56	69.62	86.71
Vegfa	NM_001025250.3	478.51	405.28	387.67	459.74
Vim	NM_011701.4	141.27	284.92	161.69	436.52
Vps4a	NM_126165.1	979.32	1001.43	980.28	1003.67
Vps4b	NM_009190.2	230.35	227.19	247.55	233.97
Was	NM_009515.2	18.85	15.06	16.12	19.39
Wdr5	NM_080848.2	150.12	162.47	148.21	152.14
Xcl1	NM_008510.1	14.18	12.74	14.94	14.99
Xiap	NM_009688.2	2154.33	2173.37	2106.69	2178.4
Xrcc6	NM_010247.2	132.73	133.61	123.05	129.28
Zbp1	NM_021394.2	13.84	15.89	14.36	15.61

Table S3. The average transcription counts for each group after normalization with housekeeping genes in the ipsilateral hippocampus.

mRNA	Accession #	Y/Sham	Y/TBI	O/Sham	O/TBI
Abcc3	NM_029600.3	26.72	35.02	34.68	43.59
Abcc8	NM_011510.3	58.96	52.73	56.68	49.13
Abl1	NM_009594.4	248.38	263.55	235.67	236.33
Adamts16	NM_172053.2	20.69	25.76	16.07	16.38
Ago4	NM_153177.3	52.88	54.55	38.99	35.12
Agt	NM_007428.3	217.62	282.92	136.36	184.04
AI464131	NM_001085515.2	231.81	259.86	231.57	229.88
Ak1	NM_001198790.1	1330.95	1360.14	1387.72	1265.06
Akt1	NM_001165894.1	904.8	892.77	907.68	840.72
Akt2	NM_001110208.1	292.23	276.22	300.29	266.4
Aldh1l1	NM_027406.1	463.07	570.52	493.72	513.22
Ambra1	NM_001080754.1	878.68	859.92	815.37	789.33
Amigo2	NM_178114.4	444.59	424.66	430.66	484.01
Anapc15	NM_027532.3	205.26	226.94	210.5	216.54
Anxa1	NM_010730.2	42.25	44.85	35.53	40.91
Apc	NM_007462.3	3818.28	4120.46	3850.84	3716.37
Apex1	NM_009687.2	266.02	300.91	288.69	292.68
Apoe	NM_001305844.1	6600.8	8254.36	7177.29	8853.14
Arc	NM_018790.2	737.9	463.96	359.1	371.26
Arhgap24	NM_029270.2	98.73	91.02	69.74	79.14
Arid1a	NM_001080819.1	514.1	500.65	496.89	480.4
Asb2	NM_023049.1	18.9	15.71	16.33	18.2
Ash2l	NM_001080793.1	404.36	384.93	391.12	384.46
Asph	NM_001177849.1	2872.55	2970.81	3031.95	2983.54
Atf3	NM_007498.3	16.44	19.74	17.77	22.11
Atg14	NM_172599.4	266.91	264.56	259.64	253.96
Atg3	NM_026402.3	1511.13	1537.08	1514.41	1466.5
Atg5	NM_001314013.1	583.26	648.3	570.09	559.16
Atg7	NM_028835.1	229.25	225.33	231.94	239.47
Atg9a	NM_001003917.3	1201.13	1197.31	1213.14	1114.7
Atm	NM_007499.2	250.61	259.32	263.09	260.3
Atp6v0e	NM_025272.2	212.32	280.06	232.97	252.62
Atp6v1a	NM_007508.5	5887.13	6281.17	6539.3	6185.58
Atr	NM_019864.1	123.58	145.79	133.61	124.68
Axl	NM_009465.3	319.72	362.83	325.75	354.58
B3gnt5	NM_001159407.1	15.18	20.87	18.56	18.99
Bad	NM_007522.3	388.12	413.25	394.62	368.74
Bag3	NM_013863.4	93.97	100.03	87.2	100.18

Bag4	NM_026121.3	1207.69	1193.73	1227.31	1120.57
Bak1	NM_007523.2	203.38	199.45	198.35	209.55
Bard1	NM_007525.3	25.65	20.24	16.11	20.62
Bax	NM_007527.3	198.23	206.43	206.5	190.1
Bbc3	NM_133234.1	58.72	47.69	44.19	50.4
Bcas1	NM_029815.2	1543.93	1795.6	1510.41	1628.77
Bcl10	NM_009740.1	281.23	299.09	286.6	282.57
Bcl2	NM_009741.3	90.65	100.11	77.56	81.17
Bcl2a1a	NM_009742.3	52.12	77.89	84.73	128.03
Bcl2l1	NM_009743.4	710.19	733.02	713.52	710.96
Bcl2l11	NM_001284410.1	34	32.97	32.14	35.28
Bcl2l2	NM_007537.1	1429.72	1441.53	1421.08	1347.61
Bdnf	NM_007540.4	401.36	329.23	414.64	357.59
Becn1	NM_019584.3	1761.66	1733.19	1741.41	1709.54
Bid	NM_007544.3	176.63	186.31	172.11	176.12
Bik	NM_007546.2	11.49	11.17	10.99	12.77
Bin1	NM_001083334.1	1676.69	1784.47	1714.78	1737.76
Birc2	NM_007465.2	473.08	516.67	478.91	447.63
Birc3	NM_007464.3	16.41	20.73	17.72	19.48
Birc5	NM_009689.2	27.52	22.21	20.11	22.35
Blk	NM_007549.2	16.13	11.52	15.98	16.41
Blm	NM_001042527.2	44.39	40.8	47.39	42.74
Blnk	NM_008528.4	59.45	69.33	82.15	84.57
Bmi1	NM_007552.4	565.53	573.64	585.21	570.17
Bnip3	NM_009760.4	1989.96	2103.78	2097.21	2079.87
Bnip3l	NM_009761.3	1344.09	1508.44	1372.86	1370.46
Bok	NM_016778.2	593.66	536.6	660.12	584.95
Bola2	NM_175103.3	1520.33	1521.88	1483.14	1522.58
Braf	NM_139294.5	1164.63	1186.35	1260.98	1209.44
Brca1	NM_009764.3	11.69	12.68	10.08	13.03
Brd2	NM_010238.3	1407.08	1393.29	1414.94	1413.66
Brd3	NM_001113573.1	393.83	414.46	381.47	373.45
Brd4	NM_001286630.1	659.4	614.26	626.14	582.18
Btk	NM_013482.2	34.65	29.29	33.28	37.41
C1qa	NM_007572.2	593.23	845.73	912.75	1253.57
C1qb	NM_009777.2	958.01	1325.67	1381.69	1816.46
C1qc	NM_007574.2	896.28	1288.49	1357.16	1707.8
C3	XM_011246258.1	41.69	83.12	81.33	174.8
C3ar1	NM_009779.2	51.37	79.16	78.39	107.9
C4a	NM_011413.2	332.93	969.43	1135.46	1833.68
C5ar1	NM_007577.3	26.46	34.11	29.24	37.11

C6	NM_016704.2	9.92	9.98	9.25	11.98
Cables1	NM_001146287.1	358.16	446.04	406.47	392.41
Calcoco2	NM_001271018.1	12.11	10.02	12.28	12.93
Calr	NM_007591.3	6949.05	6919.78	6293.44	6626.16
Camk4	NM_009793.3	981.82	849.82	1002.03	876.73
Casp1	NM_009807.2	49.19	56.86	71.47	74.14
Casp2	NM_007610.1	163.35	163	145.15	137.35
Casp3	NM_009810.2	123.87	133.07	111.19	120.65
Casp4	NM_007609.2	17.54	23.55	21.81	31.1
Casp6	NM_009811.3	67.5	73.56	66.46	73.29
Casp7	NM_007611.2	48.21	44.51	47.85	47.36
Casp8	NM_009812.2	53.92	57.81	51.4	60.45
Casp9	NM_015733.4	146.58	157.36	168.62	157.38
Cass4	NM_001080820.2	11.94	12.83	12.81	12.07
Ccl2	NM_011333.3	14	26.89	24.12	34.51
Ccl3	NM_011337.1	16.7	26.55	27.32	35.39
Ccl4	NM_013652.1	13.41	14.1	14.11	15.19
Ccl5	NM_013653.1	10.25	15.02	16.2	23.74
Ccl7	NM_013654.3	12.21	14.24	11.8	13.3
Ccng2	NM_007635.4	636.8	560.96	610.2	530.45
Ccni	NM_017367.3	2315.19	2351.17	2231	2156.66
Ccr2	NM_009915.2	21.68	19.42	16.79	16.8
Ccr5	NM_009917.5	50.69	51.64	48.07	51.87
Cd109	NM_153098.3	94.3	109.03	104.34	110.14
Cd14	NM_009841.3	24.51	30.47	36.57	45.46
Cd163	NM_053094.2	26.03	34.73	30.85	31.29
Cd19	NM_009844.2	11.51	10.77	9.25	11.98
Cd209e	NM_130905.2	11.54	12.4	10.05	12.88
Cd244	NM_018729.2	11.78	14.54	11.78	16.07
Cd24a	NM_009846.2	162.8	159.38	123.23	143.51
Cd300lf	NM_001169153.1	14.12	15.16	12.29	14.92
Cd33	NM_001111058.1	59.06	64.22	73.27	80.19
Cd36	NM_007643.3	37.1	45.55	39.46	42.92
Cd3d	NM_013487.2	10.58	10.73	11.42	13.05
Cd3e	NM_007648.4	13.67	16.16	16.44	15.87
Cd3g	NM_009850.2	12.21	14.54	16.67	16.9
Cd40	NM_011611.2	21.25	19.75	21.28	22.1
Cd44	NM_009851.2	27.12	20.38	22.7	20.48
Cd47	NM_010581.3	1933.92	1805.07	1891.64	1752.69
Cd6	NM_001037801.2	15.02	17.1	18.07	18.03
Cd68	NM_009853.1	143.79	194.51	207.37	273.77

Cd69	NM_001033122.3	14.54	11.97	15.57	16.84
Cd70	NM_011617.1	12.95	13.7	11.76	17.86
Cd72	NM_001110320.1	22.21	18.12	21.49	27.34
Cd74	NM_001042605.1	106.16	158.7	103.4	205.68
Cd83	NM_009856.2	288.64	270.16	294.6	248.53
Cd84	NM_013489.2	72.04	84.17	104.14	138.13
Cd86	NM_019388.3	41.21	44.37	46.75	56.31
Cd8a	NM_001081110.2	9.92	10.12	9.69	12.26
Cd8b1	NM_009858.2	13.45	11.51	10.84	13.67
Cdc25a	NM_007658.3	118.28	125.41	106.25	106.93
Cdc7	NM_001271566.1	99.01	101.21	89.55	80.91
Cdk20	NM_053180.2	92.25	99.84	72.35	71.17
Cdkn1a	NM_007669.4	68.95	62.87	67.95	66.68
Cdkn1c	NM_009876.3	89.48	121.82	74	142.87
Ceacam3	NM_054059.1	11.38	12.63	12.12	13.7
Cflar	NM_207653.3	169.55	226.37	179.59	218
Ch25h	NM_009890.1	9.92	10.16	10.15	12.07
Chek1	NM_007691.5	23.95	19.64	22.84	23.49
Chek2	NM_016681.3	26.79	23.6	22.03	27.43
Chn2	NM_001163640.1	225.42	249.46	213.9	206.98
Chst8	NM_175140.4	191.32	222.03	215.37	227.25
Chuk	NM_001162410.1	562.96	621.95	535.78	513.63
Cidea	NM_007702.2	110.54	136.35	114.14	128.56
Cideb	NM_009894.3	60.6	62.75	59.76	54.99
Cks1b	NM_016904.1	48.2	48.16	46.95	50.44
Clcf1	NM_019952.3	11.97	10.88	10.92	13.61
Cldn5	NM_013805.4	364.95	316.66	196.08	174.25
Clec7a	NM_020008.2	12.89	26.41	34.06	54.95
Clic4	NM_013885.2	537.09	603.64	516.07	548.46
Cln3	NM_001146311.1	143.74	168.89	144.53	142.8
Clstn1	NM_023051.4	2559.4	2589.02	2767.99	2597.13
Cnn2	NM_007725.2	53.99	57.89	49.31	52.4
Cnp	NM_009923.2	5058.39	5887.03	5225.35	5677.81
Cntnap2	NM_001004357.2	532.66	530.39	528.59	486.73
Coa5	NM_198006.4	810.34	814.34	810.02	770.91
Col6a3	XM_897036.2	43.49	32.3	49.62	61.46
Cotl1	NM_028071.3	1069.75	1070.55	1089.27	1210.71
Cox5b	NM_009942.2	9654.64	10255.82	9929.24	9661.41
Cp	NM_001042611.1	201.43	292.48	191.08	272.24
Cpa3	NM_007753.2	17	13.35	13.07	13.38
Creb1	NM_001037726.1	352.67	371.7	342.04	339.74

Crebbp	NM_001025432.1	2022.14	2047.43	1971.95	1832.93
Crem	NM_001110853.1	176.08	165.79	141.51	143.64
Crip1	NM_007763.3	185.1	231.55	171.15	178.21
Cryba4	NM_021351.1	25.41	21.93	26.36	28.76
Csf1	NM_001113530.1	338.27	398.36	345.89	411.29
Csf1r	NM_001037859.1	579.7	698.41	682.38	728.29
Csf2rb	NM_007780.4	32.6	31.27	36.92	31.15
Csf3r	NM_001252651.1	46.07	51.42	49.35	58.37
Csk	NM_007783.2	298.35	346.12	267.18	276.22
Cst7	NM_009977.3	12.05	12.88	11.3	13.67
Ctse	NM_007799.3	14.83	15.48	14.6	15.75
Ctsf	NM_019861.1	853.76	984.94	854.87	848.06
Ctss	NM_021281.2	934.74	1296.43	1465.18	1852.17
Ctsw	NM_009985.4	11.91	11.37	10.97	16.2
Cx3cl1	NM_009142.3	1006.6	850	971	874.16
Cx3cr1	NM_009987.3	655.23	761.75	812.79	905.25
Cxcl10	NM_021274.1	15.35	19.68	24.77	33.58
Cxcl9	NM_008599.2	11.59	15.6	10.44	17.11
Cycs	NM_007808.4	61.31	55.38	50.35	50.62
Cyp27a1	NM_024264.3	40.53	40.28	35.59	39.45
Cyp7b1	NM_007825.4	340.59	335.82	331.41	320.13
Cytip	NM_139200.4	18.87	12.68	12.41	16.35
Dab2	NM_023118.2	26.72	42.59	20.09	35.22
Dapk1	NM_134062.1	930.1	914.38	990.14	919.4
Ddb2	NM_028119.5	69.31	67.12	71.13	65.23
Ddx58	NM_172689.3	67.87	81.01	74.12	106.42
Dicer1	NM_148948.2	311.9	325.07	308.76	309.99
Dlg1	NM_001252433.1	878.54	887.38	862.41	818.06
Dlg4	NM_001109752.1	4577.58	4464.42	4534.01	4157.89
Dlx1	NM_010053.1	185.83	146.52	174.6	175.46
Dlx2	NM_010054.2	30.92	21.57	24.08	29.95
Dna2	NM_177372.3	32.29	19.8	26.85	25.81
Dnmt1	NM_010066.3	160.01	162.77	149.45	146.44
Dnmt3a	NM_007872.4	149.89	143.9	127.65	129.74
Dnmt3b	NM_001003960.3	11.57	12.04	10.31	12.87
Dock1	NM_001033420.2	265.17	296.21	253.45	278.69
Dock2	NM_033374.3	94.17	103.55	110.72	128.15
Dot1l	NM_199322.1	122.25	116.25	116.37	105.34
Dst	NM_010081.2	759.9	806.21	736.77	731.47
Duoxa1	NM_145395.2	11.26	11.38	9.96	12.46
Dusp7	NM_153459.4	1277.4	1198.62	1255.16	1166.11

E2f1	NM_007891.4	49.86	44.83	48.81	48.33
Eed	NM_021876.3	422.7	454.4	386.36	407.49
Eef2k	NM_007908.3	220.45	226.06	226.32	212.32
Egfr	NM_207655.2	92.33	117.75	80.31	90.81
Egr1	NM_007913.5	2111.03	1442.54	1404.7	1255.99
Ehmt2	NM_145830.1	2123.2	2110.19	2095.27	1919.78
Eif1	NM_011508.1	4718.43	4663.89	4592.14	4352.89
Emcn	NM_001163522.1	82.48	73.57	62.46	65
Emp1	NM_010128.4	37.41	45.88	40.19	54.88
Enpp6	NM_177304.3	68.57	74.82	58.13	83.56
Entpd2	NM_009849.2	123.34	138.63	100.42	114.04
Eomes	NM_010136.2	14.72	15.55	11.01	20.44
Ep300	NM_177821.6	937.91	936.26	879.9	853.67
Epcam	NM_008532.2	17.03	17.55	16.1	22.21
Epg5	NM_001195633.1	361.05	375.88	358.87	366.51
Epsti1	NM_029495.2	17.01	16.61	13.69	19.16
Erbb3	NM_010153.1	109.42	134.4	92.94	106.96
Ercc2	NM_007949.4	309.38	322.23	296.34	277.43
Esam	NM_027102.3	90.46	85.49	81.87	66.52
Ets2	NM_011809.2	934.1	872.33	907.19	845.98
Exo1	NM_012012.4	10.94	9.98	13.56	13.23
Ezh1	NM_007970.1	213.27	216.26	247.61	224.55
Ezh2	NM_007971.2	251.41	249.63	197.87	179.96
F3	NM_010171.3	804.04	986.24	782.92	880.62
Fa2h	NM_178086.3	120.55	125.5	118.37	142.29
Fabp5	NM_010634.3	1925.78	2150.34	1933.31	1950.77
Fadd	NM_010175.5	30.68	33.26	30.52	30.71
Fancc	NM_007985.2	72.7	71.57	60.58	65.48
Fancd2	NM_001033244.3	16.23	14.59	15.97	14.68
Fancg	NM_053081.2	104.32	106.69	96.6	88.22
Fas	NM_007987.2	28.5	31.48	23.98	30.75
Fasl	NM_010177.3	13.61	10.8	10.66	14.69
Fbln5	NM_011812.4	47.66	62.14	39.67	56.29
Fcer1g	NM_010185.4	121.53	147.74	154.34	190.85
Fcgr1	NM_010186.5	79.28	93.37	98.18	116.67
Fcgr2b	NM_001077189.1	90.38	160.8	134.43	194.27
Fcgr3	NM_010188.5	77.27	110.67	106.09	134.04
Fcrla	NM_145141.2	14.49	13.06	12	14.73
Fcrlb	NM_001029984.2	11.38	9.98	9.25	12.78
Fcrls	NM_030707.3	239.31	298.53	234.74	265.38
Fdxr	NM_007997.1	16.61	20.8	16.83	18.67

Fen1	NM_001271614.1	124.66	110.14	128.49	121.22
Fgd2	NM_001159538.1	25.51	24.3	31.34	31.97
Fgf13	NM_010200.2	3407.77	3276.57	3576.39	3336.2
Fgl2	NM_008013.2	40.77	66.24	47.76	59.41
Fkbp5	NM_010220.3	200.09	209.27	246.45	242.62
Flt1	NM_010228.3	101.14	88.14	61.07	56.92
Fos	NM_010234.2	273.49	184.22	85.25	136.83
Foxp3	NM_054039.2	10.91	11.12	11.14	12.37
Fpr1	NM_013521.2	11.54	10.12	9.7	12.11
Fscn1	NM_007984.2	1364.65	1336.75	1344.7	1260.78
Fyn	NM_008054.2	1458	1480.32	1452.17	1404.16
Gadd45a	NM_007836.1	115.27	103.79	105.74	110.62
Gadd45g	NM_011817.2	152.93	152.06	115.59	147.23
Gal3st1	NM_001177691.1	90.14	116.13	96.1	99.64
Gba	NM_001077411.1	217.57	225.64	206.96	218.38
Gbp2	NM_010260.1	41.68	73.78	60.05	79.07
Gclc	NM_010295.2	335.96	354.39	370.68	356.32
Gdpd2	NM_023608.3	87.94	99.56	68.38	82.85
Gja1	NM_010288.3	4382.05	5593.84	3906.38	4591.54
Gjb1	NM_008124.2	179.18	202	222.52	250.85
Gna15	NM_010304.3	34.69	39.62	36.04	45.26
Gpr183	NM_183031.2	27.24	34.52	34.87	35.23
Gpr34	NM_011823.4	234.96	278.89	289.19	297.7
Gpr62	NM_001159652.1	121.88	122.21	118.37	116.78
Gpr84	NM_030720.1	17.68	18.58	25.36	24.44
Grap	NM_027817.3	50.95	50.21	48.88	51.68
Gria1	NM_001252403.1	5470.21	5097.54	5674.94	5538.88
Gria2	NM_001039195.1	5572.96	5218.06	5794.14	5407.86
Gria4	NM_001113180.1	1087.94	1190.96	996.22	985.94
Grin2a	NM_008170.2	530.66	548.23	629.33	626.11
Grin2b	NM_008171.3	3268.71	3070.69	3411.32	3291.66
Grm2	NM_001160353.1	412.58	379.79	402.19	297.88
Grm3	NM_181850.2	1297.32	1300.66	1233.34	1123.27
Grn	NM_008175.3	502.43	550.15	549.64	637.26
Gsn	NM_146120.3	552.29	755.75	631.14	781.74
Gstm1	NM_010358.5	70.7	81.16	70.02	72.91
Gzma	NM_010370.2	10.02	10.12	10.96	13.32
Gzmb	NM_013542.2	11.5	11.07	10.26	12.79
H2afx	NM_010436.2	751.81	717.18	745.73	656.07
H2-T23	NM_010398.3	51.09	55.68	57.18	74.49
Hat1	NM_026115.4	476.02	511.89	471.82	474.32

Hcar2	NM_030701.1	14.65	13.18	18.49	18.72
Hdac1	NM_008228.2	242.56	268.63	219.34	236.58
Hdac2	NM_008229.2	1092.97	1070.58	1044.17	987.43
Hdac4	NM_207225.1	252	272.76	234.77	255.49
Hdac6	NM_010413.3	167.65	182.9	178.59	180
Hdc	NM_008230.4	21.93	15.41	12.56	19.37
Hells	NM_008234.3	42.59	49.98	42.9	42.45
Hif1a	NM_010431.2	1962.44	2108.09	1938.9	1952.45
Hilpda	NM_023516.5	52.84	55.31	51.27	49.23
Hira	NM_010435.2	445.51	442.87	448.25	403.21
Hist1h1d	NM_145713.3	11.48	12.9	11.46	13.3
Hmgb1	NM_010439.3	2000.76	2146.64	2103.92	1951.81
Hmox1	NM_010442.2	51.28	55.5	67.07	65.91
Homer1	NM_147176.2	2091.51	1967.79	1978.05	1877.46
Hpgds	NM_019455.4	77.47	93.94	100.02	125.57
Hprt	NM_013556.2	964.95	988.48	957.61	935.74
Hps4	NM_138646.3	97.5	104.87	101.82	103.38
Hrk	NM_007545.2	183.72	190.61	173.42	160.22
Hsd11b1	NM_008288.2	717.26	742.3	759.01	708.12
Hspb1	NM_013560.2	83.12	91.25	54.57	72.05
Hus1	NM_008316.2	204.67	230.91	199.9	200.85
Icam2	NM_010494.1	50.57	41.43	36.22	29.4
Ifi30	NM_023065.3	70.44	80.11	70.61	90.55
Ifih1	NM_027835.2	85.33	115.32	113.89	138.83
Ifitm2	NM_030694.1	260.52	355.66	257.07	325.37
Ifitm3	NM_025378.2	200.71	368.52	321.28	564.38
Ifnar1	NM_010508.1	500.33	515.73	466.42	443.87
Ifnar2	NM_001110498.1	212.55	252.81	222.8	239.96
Igf1	NM_001111274.1	18.85	21.14	21.29	22.18
Igf1r	NM_010513.2	342.81	339.94	335.12	350.51
Igf2r	NM_010515.1	318.52	325.56	305.53	309.62
Igsf10	NM_001162884.1	47.11	46.56	39.95	44.01
Igsf6	NM_030691.1	16.31	20.77	21.36	24.13
Ikbkb	NM_010546.2	330.22	323.25	305.39	291.96
Ikbke	NM_019777.3	19.52	24.02	20.66	24.02
Ikbkg	NM_178590.2	223.83	225.1	229.84	222.04
Il10rb	NM_008349.5	184.1	202.51	173.22	181.77
Il15ra	NM_008358.2	31.97	33.22	26.45	29.55
Il1a	NM_010554.4	12.12	15.46	18.96	21.84
Il1b	NM_008361.3	14.02	10.45	12.16	14.81
Il1r1	NM_001123382.1	188.18	196.62	181.7	174.67

Il1r2	NM_010555.4	12.93	11.79	10.29	13.43
Il1rap	NM_134103.2	554.32	541.12	581.32	520.05
Il1rl2	NM_133193.3	18.78	18.48	23.36	28.54
Il1rn	NM_031167.5	13.77	11.3	10.47	15.45
Il21r	NM_021887.1	16.16	20.79	21.47	20.89
Il2rg	NM_013563.3	27.33	23.01	25.93	33.33
Il3	NM_010556.4	15.33	16.15	14.25	14.66
Il3ra	NM_008369.1	11.08	11.14	13.67	15.96
Il6ra	NM_010559.2	77.72	90.82	82.88	97.94
Inpp5d	NM_001110192.1	41.39	53.92	51.03	61.7
Iqsec1	NM_001134383.1	2048.65	2059.24	2083.2	2002.78
Irak1	NM_008363.2	186.85	233.88	189.69	211.31
Irak2	NM_001113553.1	220.32	211.33	206.41	215.67
Irak3	NM_028679.3	31.63	26.44	32.01	31.71
Irak4	NM_029926.5	44.55	58.16	54.02	58.83
Irf1	NM_008390.1	95.86	109.42	88.68	109.92
Irf2	NM_008391.2	428.27	428.32	419.46	419.06
Irf3	NM_016849.4	148.59	173.19	132.5	142.08
Irf4	NM_013674.1	16.57	12.6	14.45	16.76
Irf6	NM_016851.2	18.5	18.47	15.86	16.92
Irf7	NM_016850.2	23.33	29.14	32.57	47.39
Irf8	NM_008320.3	52.22	53.23	53.3	73.97
Islr2	NM_001161538.1	518.51	541.65	534.41	502.27
Itga6	NM_008397.3	298.16	332.36	257.75	281.93
Itga7	NM_008398.2	101.04	99.79	119.73	103.91
Itgam	NM_001082960.1	122.3	129.19	142.59	164.51
Itgav	NM_008402.2	444.79	450.04	458.56	468.16
Itgax	NM_021334.2	10.25	13.53	14.28	16.07
Itgb5	NM_001145884.1	632.88	776.15	666.16	791.56
Jag1	NM_013822.2	82.26	85.89	62.37	63.79
Jam2	NM_023844.4	353.57	380.91	350.95	338.95
Jarid2	NM_021878.2	240.5	247.47	228.9	217.24
Jun	NM_010591.2	931.23	1009.79	918.85	883.8
Kat2a	NM_020004.5	506.4	478.3	494.55	470.32
Kat2b	NM_020005.3	299.08	374.67	310.56	337.4
Kcnd1	NM_008423.1	41.81	47.5	45.02	44.1
Kcnj10	NM_001039484.1	916.59	992.25	727.79	833.62
Kcnk13	NM_146037.1	69.63	69.01	64.99	69.5
Kdm1a	NM_133872.1	893.13	943.97	895.34	856.04
Kdm1b	NM_172262.3	102.92	102.41	98.01	99.38
Kdm2a	NM_001001984.2	605.86	557.83	536.72	521.6

Kdm2b	NM_001003953.1	167.93	167.17	162.28	179.48
Kdm3a	NM_001038695.2	389.14	382.65	334.14	331.18
Kdm3b	NM_001081256.1	17.32	14.4	19.31	19.77
Kdm4a	NM_172382.2	246.35	245.47	240.43	248
Kdm4b	NM_172132.1	492.81	490.54	523.84	494.31
Kdm4c	NM_144787.1	356.22	353.96	361.36	334.25
Kdm4d	NM_173433.2	25.85	23.01	25.54	26.85
Kdm5a	XR_377436.1	455.84	458.12	436.22	431.94
Kdm5b	NM_152895.2	748.32	757.17	710.81	714.24
Kdm5c	NM_013668.3	508.62	535.09	477.46	466.65
Kdm5d	NM_011419.3	226.88	244.36	215.63	210.22
Kdm6a	NM_009483.1	173.39	161.94	167.16	170.75
Kif2c	NM_134471.3	15.23	10.87	12.75	14.05
Kir3dl1	NM_177749.3	12.21	10.75	12.08	12.77
Kir3dl2	NM_177748.2	21.54	23.13	19.31	21.13
Kit	NM_001122733.1	259.77	330.65	315.38	314.02
Klrb1	NM_001099918.1	11.56	10.75	12.05	12.66
Klrd1	NM_010654.2	11.08	12.21	9.94	13.99
Klrk1	NM_001083322.1	18.32	15.74	16.52	19.39
Kmt2a	NM_001081049.1	577.05	564.39	553.07	511.91
Kmt2c	NM_001081383.1	520.07	535.91	492.55	456.14
Lacc1	NM_172488.2	79.26	90.26	93.16	95.85
Lag3	NM_008479.1	54.43	74.12	88.88	103.21
Lair1	NM_001113474.1	98.26	115.33	128.42	133.74
Lamp1	NM_010684.2	4161.4	4749.62	4317.45	4600.74
Lamp2	NM_001017959.1	1751.75	2229.7	1925.79	2189.3
Lcn2	NM_008491.1	23.37	22.54	70.59	79.33
Ldha	NM_010699.2	3267.85	3229.14	3360.13	3350.58
Ldlrad3	NM_178886.2	140.79	164.24	137.52	133.14
Lfng	NM_008494.3	294.24	346.64	271.02	336.23
Lgmn	NM_011175.3	1255.94	1429.95	1355.14	1522.34
Lig1	NM_001083188.1	86.05	80.4	77.71	70.73
Lilrb4a	NM_013532.3	24.68	35.84	37	62.54
Lingo1	NM_181074.4	1377.21	1465.87	1689.85	1607.14
Lmna	NM_001002011.2	363.49	366.43	385.23	380.61
Lmnb1	NM_010721.2	121.71	115.27	101.72	89.26
Lrg1	NM_029796.2	18.47	17.65	15.66	17.65
Lrrc25	NM_153074.3	13.75	16.7	18.47	19.63
Lrrc3	NM_145152.4	95.96	91.55	97.52	95.61
Lsr	NM_001164184.1	94.36	95.01	67.27	64.13
Lst1	NM_010734.2	20.68	19.7	17.14	21.76

Lta	NM_010735.2	15	15.39	14.86	14.21
Ltb	NM_008518.2	18.44	12.41	17.37	17.74
Ltbr	NM_010736.3	58.51	76.21	57.86	71.28
Ltc4s	NM_008521.1	21.05	20.84	22.12	22.25
Ly6a	NM_010738.2	223.84	226.58	248.83	217.88
Ly6g	XM_909927.2	15.21	11.67	11.8	14.24
Ly9	NM_008534.2	14.32	26.03	26.64	40.51
Lyn	NM_010747.1	74.71	86.1	92.04	115.65
Mafb	NM_010658.2	295.8	346.53	357.63	367.53
Maff	NM_010755.3	17.69	21.32	19.44	23.63
Mag	NM_010758.2	320.65	359.89	329.97	397.59
Mal	NM_001171187.1	1914.51	2093.86	1737.8	1760.25
Man2b1	NM_010764.2	387.08	463.19	456.09	516.18
Map1lc3a	NM_025735.1	1388.21	1340.94	1365.55	1386.3
Map2k1	NM_008927.3	4395.88	4272.32	4786.14	4388.72
Map2k4	NM_009157.4	2105.43	2140.37	2148.73	2088.26
Map3k1	NM_011945.2	123.88	122.05	99.79	111.14
Map3k14	NM_016896.3	23.21	23.22	22.8	23.49
Mapk10	NM_001081567.1	3744.73	3989.33	4076.29	3844.69
Mapk12	NM_013871.3	18.27	18.71	18.34	16.66
Mapk14	NM_011951.2	607.36	625.2	605.39	582.21
Mapt	NM_001038609.2	1534.29	1531.39	1508.01	1496.6
Marco	NM_010766.2	17.76	13.79	17.09	16.19
Mavs	NM_144888.2	121.27	132.76	112.02	120.24
Mb21d1	NM_173386.4	23.73	27.15	22.31	26.04
Mbd2	NM_010773.2	1467.61	1473.98	1476.17	1433.82
Mbd3	NM_013595.2	1070.08	1015.55	1024.12	977.39
Mcm2	NM_008564.2	41.25	40.02	35.18	35.79
Mcm5	NM_008566.2	60.24	59.26	62.08	57.46
Mcm6	NM_008567.1	135.84	115.08	120.83	108.8
Mdc1	NM_001010833.2	124.89	126.73	119.09	118.62
Mdm2	NM_010786.4	729.91	734.73	671.1	665.91
Mef2c	NM_001170537.1	1895.83	1723.13	2084.69	1774.92
Mertk	NM_008587.1	347.74	423.26	411	429.77
Mfge8	NM_008594.2	286.94	295.5	256.22	282.99
Mgmt	NM_008598.2	31.45	39.25	30.24	38.48
Mmp12	NM_008605.3	11.87	15.19	17.52	27.75
Mmp14	NM_008608.3	96.6	111.7	70.52	86.28
Mobp	NM_001039364.2	4026.8	4489.38	4410.36	4623.73
Mog	NM_010814.2	642.9	796.82	687.1	778.47
Mpeg1	NM_010821.1	270.99	359.28	393.35	539.81

Mpg	NM_010822.3	44.22	42.35	51.23	51.4
Mr1	NM_008209.4	108.18	143.08	119.96	147.97
Mre11a	NM_018736.2	196.46	194.12	192.4	178.03
Ms4a1	NM_007641.5	9.92	10.3	9.25	11.98
Ms4a2	NM_001276330.1	9.92	9.98	9.25	11.98
Ms4a4a	XM_003086124.1	17.6	26.6	19.24	23.69
Msh2	NM_008628.2	398.24	421.47	404.55	367.76
Msn	NM_010833.2	101.31	109	102.65	123.75
Msr1	NM_001113326.1	15.96	15.31	15.78	18
Mvp	NM_080638.2	63.09	94.43	78.58	107.63
Myc	NM_010849.4	73.69	84.24	70.46	74.35
Myct1	NM_026793.2	27.68	25.53	23.92	22.61
Myd88	NM_010851.2	58.49	56.29	55.35	55.77
Myrf	NM_001033481.1	577.52	687.21	617.09	718.22
Nbn	NM_013752.3	127.95	160.84	132.83	134.29
Ncaph	NM_144818.3	22.84	18.21	20.69	17.94
Ncf1	NM_001286037.1	51.3	63.01	66.68	76.46
Ncor1	NM_011308.2	1219.66	1179.09	1214.72	1138.18
Ncor2	NM_011424.2	695.6	659.24	635.87	603.35
Ncr1	NM_010746.3	9.92	9.98	9.25	11.98
Nefl	NM_010910.1	1360.35	1326.12	1399.62	1380.79
Nfe2l2	NR_132727.1	58.13	74.74	57.53	66.75
Nfkb1	NM_008689.2	241.46	254.27	222.04	223.48
Nfkb2	NM_019408.2	19.76	27.79	26.37	26.23
Nfkbia	NM_010907.2	71.7	103.23	76.14	94.8
Nfkbie	NM_008690.3	32.07	31.54	30.34	32.49
Ngf	NM_001112698.1	99.74	101.43	99.8	106.68
Ngfr	NM_033217.3	20.92	22.28	15.88	19.59
Ninj2	NM_016718.2	34.08	29.07	32.23	31.86
Nkg7	NM_024253.4	15.67	11.89	15.21	17.79
Nlgn1	NM_138666.3	1466.36	1520.26	1466.71	1488.72
Nlgn2	NM_198862.2	2478.15	2409.12	2494.09	2344.94
Nlrp3	NM_145827.3	15.28	19.01	15.65	21.25
Nod1	NM_172729.2	52.53	54.38	47.38	50.18
Nostrin	NM_181547.3	44.58	39.96	41.58	36.99
Npl	NM_028749.1	67.25	82.33	64.08	78.26
Npnt	NM_001029836.1	278.51	277.32	256.56	211.44
Nptx1	NM_008730.2	5924.76	5728.21	6903.89	6089.09
Nqo1	NM_008706.5	103.87	137.8	106.12	137.73
Nrgn	NM_022029.2	6196.66	5857.58	6937.61	6053.84
Nrm	NM_134122.2	25.69	26.37	23.67	27.13

Nrp2	NM_001077403.1	282.72	306.56	256.56	297.09
Nthl1	NM_008743.2	48.86	54.89	46.8	51.34
Nwd1	NM_176940.5	465.64	511.59	465.84	464.94
Oas1g	NM_011852.2	16.62	18.22	20.27	24.2
Ogg1	NM_010957.4	51.15	55.01	57.69	59.07
Olfml3	NM_133859.2	212.2	260.56	234.95	243.88
Opalin	NM_153520.1	676.26	728.94	714.93	724.6
Optn	NM_181848.4	222.66	247.27	227.98	256.36
Osgin1	NM_027950.1	16.33	14.06	18.18	21.13
Osmr	NM_011019.3	57.48	85.65	87.62	119.84
P2rx7	NM_001038839.2	161.3	189.03	180.72	201.01
P2ry12	NM_027571.3	497.89	508.2	537.33	514.96
Pacsin1	NM_011861.3	2421.95	2284.92	2466.61	2278.27
Padi2	NM_008812.2	242	348.43	252.94	313.98
Pak1	NM_011035.2	4282.77	4648.35	4331.65	3985.16
Parp1	NM_007415.2	672.49	667.49	687.66	687.86
Parp2	NM_009632.2	265.01	288.07	269.25	253.3
Pcna	NM_011045.2	747.06	799.52	764.74	751.03
Pdpn	NM_010329.2	306.46	414.52	308.73	410.44
Pecam1	NM_008816.2	194.55	172.93	153.08	118.09
Pex14	NM_019781.2	406.89	405.04	437.21	406.9
Pik3ca	NM_008839.1	679.72	763.79	657.23	633.09
Pik3cb	NM_029094.3	649.4	669.53	631.7	564.46
Pik3cd	XM_003945690.1	122.05	128.47	125.93	109.41
Pik3cg	NM_020272.2	30.89	41.05	42.07	45.54
Pik3r1	NM_001024955.1	740.61	870.3	764.68	818.74
Pik3r2	NM_008841.2	1112.56	1107.59	1170.01	1088.59
Pik3r5	NM_177320.2	17.82	16.43	13.01	20.48
Pilra	NM_153510.3	12.2	12.93	15.05	13.72
Pilrb1	NM_133209.2	11.04	9.98	9.81	12.44
Pink1	NM_026880.2	3634.29	3867.96	3918.17	3637.18
Pla2g4a	NM_008869.2	37.34	43.08	42.09	47.48
Pla2g5	NM_001122954.1	35.09	43.86	28.36	52.42
Plcg2	NM_172285.1	88.45	93.26	104.83	108.83
Pld1	NM_001164056.1	62.78	74.68	66.16	68.9
Pld2	NM_008876.2	65.82	72.88	57.56	67.89
Plekhb1	NM_001163184.1	4428.82	5205.42	4363.31	4831.17
Plekhm1	NM_183034.1	199.69	207.98	195.97	179.5
Pllp	NM_026385.3	148.18	168.66	160.28	168.56
Plp1	NM_011123.2	12449.08	15338.39	12677.84	14762.99
Plxdc2	NM_026162.5	696.49	740.6	645.76	710.98

Plxnb3	NM_019587.2	125.92	168.45	136.53	165.55
Pmp22	NM_008885.2	174.66	216.78	202.65	242.05
Pms2	NM_008886.2	331.19	338.42	346.93	331.63
Pnoc	NM_001205075.1	47.75	41.99	37.99	45.51
Pole	NM_011132.2	23.96	23.21	25.19	24.73
Ppfia4	NM_001144855.1	318.42	275.18	319.06	277.55
Ppp3ca	NM_008913.4	18273.11	15385.3	19444.64	16578.58
Ppp3cb	NM_008914.2	4175.49	4146.22	4514.75	4187.42
Ppp3r1	NM_024459.2	6432.52	5682.03	6943.94	6215.81
Ppp3r2	NM_001004025.4	27.32	29.02	25.95	21.08
Prdx1	NM_011034.4	228.48	239.28	217.55	242.38
Prf1	NM_011073.2	9.92	10.12	9.38	12.57
Prkaca	NM_008854.3	3676.5	3591.17	3668.59	3502.61
Prkacb	NM_011100.3	3000.86	2763.18	2820.88	2658.42
Prkar1a	NM_021880.2	6877.89	6816.97	6821.16	6654.55
Prkar2a	NM_008924.2	762.86	756.49	733.24	701.66
Prkar2b	NM_011158.3	614.82	523.62	508.24	458.62
Prkce	NM_011104.2	5249.66	5032.4	5769.75	5312.1
Prkcq	NM_008859.2	106.51	111.9	82.71	95.53
Prkdc	NM_011159.2	219.29	237.33	246.6	234.29
Pros1	NM_011173.2	110.2	140.75	131.69	151.1
Psen2	NM_001128605.1	141.73	144.37	138.96	136.97
Psmb8	NM_010724.2	94.57	115.51	121.26	158.25
Pten	NM_008960.2	2376.66	2398.69	2513.49	2354.12
Ptger3	NM_011196.2	19.15	18.16	19.24	18.45
Ptger4	NM_008965.1	18.21	13.54	13.59	15.27
Ptgs2	NM_011198.3	225	232.54	314.76	281.4
Ptms	NM_026988.2	6160.18	5960.72	6433.71	6132.83
Ptpn6	NM_013545.2	56.17	59.03	58.09	77.92
Ptprc	NM_011210.3	50.55	53.42	57.22	82.29
Pttg1	NM_001131054.1	663.64	764.78	715.45	693.78
Ptx3	NM_008987.3	23.61	20.43	19.78	27.39
Pycard	NM_023258.4	10.85	11.88	10.61	14.16
Rab6b	NM_173781.4	4113.76	4016.74	4139.78	3799.49
Rab7	NM_009005.2	4000.11	3959.54	4221.03	4099.56
Rac1	NM_009007.2	3535.67	3761.38	3726.41	3720.36
Rac2	NM_009008.3	23.08	24.79	24.54	26.94
Rad1	NM_011232.2	158.9	189.01	166.57	154.91
Rad17	NM_001044371.1	152.4	152.6	137.21	147.71
Rad50	NM_009012.2	139.95	145.73	152.1	151.41
Rad51	NM_011234.4	23.39	28.65	22.35	24.69

Rad51b	NM_009014.3	15.02	13.2	13.27	15.72
Rad51c	NM_053269.3	44.84	48.41	49.37	45.49
Rad9a	NM_011237.2	53.05	53.11	45.89	42.29
Rag1	NM_009019.2	11.75	12.04	11.92	12.55
Rala	NM_019491.5	696.48	758.33	681.42	660.17
Ralb	NM_022327.5	284.46	322.97	288.69	281.65
Rapgef3	NM_001177810.1	238.02	291.51	221.35	264.5
Rb1cc1	NM_009826.4	1113.25	1168.27	1119.74	1041.16
Rbfox3	NM_001024931.2	2501.55	2194.62	2697.37	2263.55
Rela	NM_009045.4	146.59	145.97	132.65	134.53
Relb	NM_009046.2	142.18	157.1	149.95	174.97
Reln	NM_011261.2	291.3	275	237.29	233.46
Rgl1	NM_016846.3	360.15	374.01	381.81	383.7
Rhoa	NM_016802.4	1809.43	1832.44	1791.61	1874.93
Ripk1	NM_009068.3	34.46	42.18	33.76	41.83
Ripk2	NM_138952.3	107.87	101.7	105.25	89.36
Rnf8	NM_021419.2	322.85	334.94	312.48	296.57
Rpa1	NM_026653.2	688.52	756.37	718.52	756.33
Rpl28	NM_009081.2	4273.11	4647.92	4195.58	4174.36
Rpl29	NM_009082.2	2313.87	2423.42	2411.04	2392.7
Rpl36al	NM_025589.4	1063.34	1126.19	1006.64	1008.61
Rpl9	NM_011292.2	4218.41	4246.96	3940.07	3934.36
Rps10	NM_025963.3	901.91	970.96	958.87	1032.08
Rps2	NM_008503.5	3938.33	3855.87	3899.85	3867.71
Rps21	NM_025587.2	11812.07	12045.04	11961.6	11894.85
Rps3	NM_012052.2	224.51	239.24	220.57	199.47
Rps9	NM_029767.2	4035.87	4260.72	3850	4132.79
Rrm2	NM_009104.1	19.73	18.52	15.59	14.97
Rsad2	NM_021384.2	54.62	45.73	56.6	68.66
Rtn4rl1	NM_177708.4	559.97	642.58	657.28	638.43
S100a10	NM_009112.2	640.61	890.82	698.75	839.71
S100b	NM_009115.3	1068.09	1400.04	1296.86	1457.12
S1pr3	NM_010101.3	105.24	146.98	112.96	176.86
S1pr4	NM_010102.2	16.26	12.02	10.43	13.83
S1pr5	NM_053190.2	87.41	88.33	92.2	96.02
Sall1	NM_021390.3	326.55	373.15	347.45	377.73
Sell	XM_006496716.1	18.18	12.32	11.75	12.59
Serpina3n	NM_009252.2	168.51	218.1	239.05	380.52
Serpine1	NM_008871.2	15	16.42	18.47	24.1
Serpinf1	NM_011340.3	239.81	288.38	236.25	232.6
Serping1	NM_009776.3	64.87	112.74	77.45	100.36

Sesn1	NM_001013370.2	575.28	586.03	585.72	516.6
Sesn2	NM_144907.1	89.8	93.08	79.9	88.38
Setd1a	NM_178029.3	157.62	152.76	155.88	152.35
Setd1b	NM_001040398.1	509.99	543.1	485.45	472.9
Setd2	NM_001081340.2	1159.46	1163.67	1096.79	1033.81
Setd7	NM_080793.5	530.56	517.77	488.01	444.61
Setdb1	NM_018877.2	143.01	135.27	124.72	132.31
Sftpd	NM_009160.2	15.25	15.72	11.86	13.57
Sh2d1a	NR_132588.1	10.68	10.27	9.25	12.51
Shank3	NM_021423.3	975.2	902.98	952.87	875
Siglec1	NM_011426.3	12.65	11.41	13.11	14.97
Siglecf	NM_145581.1	16.22	14.98	21.1	22.89
Sin3a	NM_001110350.1	473.13	476.22	441.85	421.15
Sirt1	NM_019812.2	245.01	251.18	237.11	220.74
Slamf8	NM_029084.3	11.36	14.95	14.79	17.23
Slamf9	NM_029612.4	26.98	37.04	45.67	58.24
Slc10a6	NM_029415.2	13.61	14.93	13.36	17.79
Slc17a6	NM_080853.3	508.43	561.68	253.49	236.13
Slc17a7	NM_182993.2	5631.11	6060.48	7397.46	6734.04
Slc1a3	NM_148938.3	5728.18	6532.98	6136.7	6433.87
Slc2a1	NM_011400.3	205.03	236.52	212.45	220.57
Slc2a5	NM_019741.3	76.48	71.74	76.63	73.2
Slc44a1	NM_001159633.1	1164.71	1352.93	1267.47	1369.33
Slc6a1	NM_178703.4	2474.4	2614.64	2429.83	2414.58
Slco2b1	NM_175316.3	119.75	136.03	121.98	136.08
Slfn8	NM_181545.4	27.27	33.4	35.9	50.58
Smarca4	NM_011417.2	1027.42	1068.03	965.54	950.27
Smarca5	NM_053124.2	1687.97	1745	1620	1635.29
Smarcd1	NM_031842.1	366.35	361.78	391.52	363.49
Smc1a	NM_019710.2	598.41	633.82	571.83	548.67
Snca	NM_009221.2	2647.78	2831.95	2877	2747.03
Socs3	NM_007707.2	26.94	29.02	26.1	34.36
Sod2	NM_013671.3	2790.88	2827.06	2739.6	2599.32
Sox10	XM_128139.6	212.13	243.99	217.18	229.24
Sox4	NM_009238.2	168.32	168.75	136.21	141.48
Sox9	NM_011448.4	601.33	719.76	558.74	671.04
Sphk1	NM_011451.3	19.7	21.8	15.5	15.71
Spib	NM_019866.1	13.2	10.75	12.86	13.07
Spint1	NM_016907.3	17.98	20.51	22.32	23.39
Spp1	NM_009263.3	213.14	424.9	217.39	296.35
Sqstm1	NM_011018.2	2255.11	2199.3	2370.89	2346.89

Srgn	NM_011157.2	137.28	158.28	144.18	143.14
Srxn1	NM_029688.4	665.67	628.7	655.2	618.49
St3gal6	NM_018784.2	211.1	227.21	194.13	199.12
St8sia6	NM_145838.1	83.86	69.38	83.49	89.31
Stat1	NM_009283.3	190.59	218.19	217.85	244.42
Steap4	NM_054098.3	15.11	15.57	13.84	21.91
Stmn1	NM_019641.3	5876.9	6672.46	5546.85	5184.21
Stx18	NM_026959.2	100.69	120.45	106.13	110.27
Sumo1	NM_009460.1	3438.04	3450.2	3343.97	3242.54
Suv39h1	NM_011514.2	354	354.75	356.3	348.47
Suv39h2	NM_022724.4	87.57	84.21	78.49	77.07
Suz12	NM_199196.1	857.41	871.1	839.96	826.02
Syk	NM_001198977.1	45.22	47.78	47.62	46.91
Syn2	NM_013681.1	5243.03	5260.56	5769.78	5566.28
Syp	NM_009305.2	7607.04	7558.78	7895.15	7676.73
Tarbp2	NM_001253795.1	141.37	147.65	138.65	122.01
Tbc1d4	NM_001081278.2	54.67	51.57	45.79	46.96
Tbr1	NM_009322.3	196.73	202.85	235.83	176.85
Tbx21	NM_019507.2	10.92	9.98	9.85	12.71
Tcirg1	NM_001136091.1	80.44	94.07	71.61	95.83
Tcl1	NM_009337.3	10.26	10	9.25	11.98
Tet1	NM_027384.1	241.94	220.4	199.06	183.49
Tfg	NM_001252443.1	1340.35	1355.93	1315.53	1324.32
Tgfa	NM_031199.2	257.15	238.55	240.6	242.97
Tgfb1	NM_011577.1	78.43	74.84	75.93	90.52
Tgfbr1	NM_009370.2	495.48	537.74	519.55	516.89
Tgm1	NM_001161714.1	9.92	12.2	12.56	17.78
Tgm2	NM_009373.3	59.74	58.18	60.72	58.75
Tie1	NM_011587.2	74.12	55.57	44.43	34.99
Timeless	NM_011589.1	38.67	43.55	33.46	36.74
Timp1	NM_011593.2	9.92	11.12	10.27	13.7
Tle3	NM_009389.2	319.25	411.47	339.15	389.43
Tlr2	NM_011905.2	14.83	15.73	14.74	26.23
Tlr4	NM_021297.2	33.91	55.12	36.53	53.88
Tlr7	NM_133211.3	22.83	28.98	29.56	35.93
Tm4sf1	NM_008536.3	220.99	310.51	226.65	243.34
Tmc7	NM_172476.4	119.52	118.65	100.84	104.34
Tmcc3	NM_172051.2	611.14	657.89	623.19	660.78
Tmem100	NM_026433.2	217.82	263.61	209.98	229.92
Tmem119	NM_146162.2	325.96	366.17	410.08	398.66
Tmem144	NM_027495.4	167.98	167.91	168.64	158.64

Tmem173	NM_028261.1	23.02	24.29	28.72	32.82
Tmem204	NM_001001183.1	32.03	43.55	34.03	28.72
Tmem206	NM_025864.3	212.19	203.12	179.66	192.19
Tmem37	NM_019432.2	22.76	31.08	27.29	31.18
Tmem64	NM_181401.3	996.99	1035.96	895.14	855.72
Tmem88b	NM_001033394.3	311.82	359.07	342.58	386.69
Tnf	NM_013693.2	12.12	11.89	11.23	12.33
Tnfrsf10b	NM_020275.3	20.78	18.58	16.92	18.26
Tnfrsf11b	NM_008764.3	15.58	19.49	17.74	20.43
Tnfrsf12a	NM_001161746.1	71.97	73.55	61.87	71.03
Tnfrsf13c	NM_028075.2	14.71	13.16	15.49	16.5
Tnfrsf17	NM_011608.1	17.52	18.71	22.25	17.81
Tnfrsf1a	NM_011609.2	129.21	170.09	131.15	166.1
Tnfrsf1b	NM_011610.3	32.1	29.64	28.26	30.49
Tnfrsf25	NM_033042.3	71.94	65.82	54.08	49.71
Tnfrsf4	NM_011659.2	10.18	11.64	10.87	12.41
Tnfsf10	NM_009425.2	27.27	29.33	29.09	28.6
Tnfsf12	NM_011614.3	95.57	99.97	94.17	92.2
Tnfsf13b	NM_033622.1	19.51	16.14	21.07	21.91
Tnfsf4	NM_009452.2	9.92	10.13	9.25	12.26
Tnfsf8	NM_009403.2	11.31	10.3	10.51	13.46
Top2a	NM_011623.2	34.12	25.32	21.47	25
Topbp1	NM_176979.5	90.34	103.02	100.92	99.8
Tpd52	NM_001025262.1	1218.75	1179.86	1213.76	1151.52
Tpsb2	NM_010781.3	9.92	10.96	9.25	12.93
Tradd	NM_001033161.2	37.53	37.58	35.29	34
Traf1	NM_009421.3	21.99	28.82	22.53	32.56
Traf2	NM_009422.2	179.97	184.7	150.66	159.63
Traf3	NM_011632.3	400.34	428.53	379.13	396.09
Traf6	NM_009424.2	223.91	241.17	210.53	217.24
Trat1	NM_198297.3	11.88	13.34	12.37	13.6
Trem1	NM_021406.5	10.73	9.98	9.7	12.39
Trem2	NM_031254.2	190.69	268.77	283.08	367.69
Trem3	NM_021407.3	13.89	12.72	11.42	12.26
Trim47	NM_001205081.1	67.39	71.62	61.73	64.15
Trp53	NM_011640.1	114.29	116.75	107.44	115.34
Trp53bp2	NM_173378.2	229.94	262.63	243.23	240.63
Trp73	NM_011642.3	51.87	59.13	42.29	51.21
Trpa1	NM_177781.4	13.7	13.07	11.47	15.12
Trpm4	NM_175130.4	31.36	32.29	35.04	34.69
Tspan18	NM_183180.2	126.09	169.33	154.96	156.89

Ttr	NM_013697.4	647.54	708.58	309.8	2289.91
Tubb3	NM_023279.2	3034.09	2922.86	2820.36	2672
Tubb4a	NM_009451.3	1284.55	1376.01	1291.85	1228.83
Txnrd1	NM_015762.2	875.7	839.22	806.73	777.02
Tyrobp	NM_011662.2	380.98	494.03	505.17	672.92
Ugt8a	NM_011674.4	504.03	563.34	468.53	525.24
Ulk1	NM_009469.3	833.56	852.07	780.43	771.5
Ung	NM_001040691.1	36.66	33.02	32.64	30.41
Uty	NM_009484.2	243.55	284.64	247.79	239.07
Vamp7	NM_011515.4	1132.25	1198.68	1168.01	1105.45
Vav1	NM_011691.4	75.25	91.33	88.14	102.05
Vegfa	NM_001025250.3	450.09	427.13	361.55	397.23
Vim	NM_011701.4	397.74	803.04	512.89	955.62
Vps4a	NM_126165.1	870.26	889.94	869.14	896.47
Vps4b	NM_009190.2	237.47	238.21	237.35	237.72
Was	NM_009515.2	20.59	20.82	19.68	26.29
Wdr5	NM_080848.2	147.1	147.92	149.38	136.98
Xcl1	NM_008510.1	12.5	15.94	18.09	16.63
Xiap	NM_009688.2	1952.93	2017.95	1872.62	1812.19
Xrcc6	NM_010247.2	156.17	159.73	156.13	142.89
Zbp1	NM_021394.2	12.24	11.43	15.62	21.7
Zfp367	NM_175494.4	280.76	312.77	310.17	306.06
